# The performance of the jet trigger for the ATLAS detector during 2011 data taking

**DOI:** 10.1140/epjc/s10052-016-4325-0

**Published:** 2016-09-27

**Authors:** G. Aad, B. Abbott, J. Abdallah, O. Abdinov, B. Abeloos, R. Aben, M. Abolins, O. S. AbouZeid, N. L. Abraham, H. Abramowicz, H. Abreu, R. Abreu, Y. Abulaiti, B. S. Acharya, L. Adamczyk, D. L. Adams, J. Adelman, S. Adomeit, T. Adye, A. A. Affolder, T. Agatonovic-Jovin, J. Agricola, J. A. Aguilar-Saavedra, S. P. Ahlen, F. Ahmadov, G. Aielli, H. Akerstedt, T. P. A. Åkesson, A. V. Akimov, G. L. Alberghi, J. Albert, S. Albrand, M. J. Alconada Verzini, M. Aleksa, I. N. Aleksandrov, C. Alexa, G. Alexander, T. Alexopoulos, M. Alhroob, M. Aliev, G. Alimonti, J. Alison, S. P. Alkire, B. M. M. Allbrooke, B. W. Allen, P. P. Allport, A. Aloisio, A. Alonso, F. Alonso, C. Alpigiani, B. Alvarez Gonzalez, D. Álvarez Piqueras, M. G. Alviggi, B. T. Amadio, K. Amako, Y. Amaral Coutinho, C. Amelung, D. Amidei, S. P. Amor Dos Santos, A. Amorim, S. Amoroso, N. Amram, G. Amundsen, C. Anastopoulos, L. S. Ancu, N. Andari, T. Andeen, C. F. Anders, G. Anders, J. K. Anders, K. J. Anderson, A. Andreazza, V. Andrei, S. Angelidakis, I. Angelozzi, P. Anger, A. Angerami, F. Anghinolfi, A. V. Anisenkov, N. Anjos, A. Annovi, M. Antonelli, A. Antonov, J. Antos, F. Anulli, M. Aoki, L. Aperio Bella, G. Arabidze, Y. Arai, J. P. Araque, A. T. H. Arce, F. A. Arduh, J-F. Arguin, S. Argyropoulos, M. Arik, A. J. Armbruster, L. J. Armitage, O. Arnaez, H. Arnold, M. Arratia, O. Arslan, A. Artamonov, G. Artoni, S. Artz, S. Asai, N. Asbah, A. Ashkenazi, B. Åsman, L. Asquith, K. Assamagan, R. Astalos, M. Atkinson, N. B. Atlay, K. Augsten, G. Avolio, B. Axen, M. K. Ayoub, G. Azuelos, M. A. Baak, A. E. Baas, M. J. Baca, H. Bachacou, K. Bachas, M. Backes, M. Backhaus, P. Bagiacchi, P. Bagnaia, Y. Bai, J. T. Baines, O. K. Baker, E. M. Baldin, P. Balek, T. Balestri, F. Balli, W. K. Balunas, E. Banas, Sw. Banerjee, A. A. E. Bannoura, L. Barak, E. L. Barberio, D. Barberis, M. Barbero, T. Barillari, T. Barklow, N. Barlow, S. L. Barnes, B. M. Barnett, R. M. Barnett, Z. Barnovska, A. Baroncelli, G. Barone, A. J. Barr, L. Barranco Navarro, F. Barreiro, J. Barreiro Guimarães da Costa, R. Bartoldus, A. E. Barton, P. Bartos, A. Basalaev, A. Bassalat, A. Basye, R. L. Bates, S. J. Batista, J. R. Batley, M. Battaglia, M. Bauce, F. Bauer, H. S. Bawa, J. B. Beacham, M. D. Beattie, T. Beau, P. H. Beauchemin, P. Bechtle, H. P. Beck, K. Becker, M. Becker, M. Beckingham, C. Becot, A. J. Beddall, A. Beddall, V. A. Bednyakov, M. Bedognetti, C. P. Bee, L. J. Beemster, T. A. Beermann, M. Begel, J. K. Behr, C. Belanger-Champagne, A. S. Bell, G. Bella, L. Bellagamba, A. Bellerive, M. Bellomo, K. Belotskiy, O. Beltramello, N. L. Belyaev, O. Benary, D. Benchekroun, M. Bender, K. Bendtz, N. Benekos, Y. Benhammou, E. Benhar Noccioli, J. Benitez, J. A. Benitez Garcia, D. P. Benjamin, J. R. Bensinger, S. Bentvelsen, L. Beresford, M. Beretta, D. Berge, E. Bergeaas Kuutmann, N. Berger, F. Berghaus, J. Beringer, S. Berlendis, N. R. Bernard, C. Bernius, F. U. Bernlochner, T. Berry, P. Berta, C. Bertella, G. Bertoli, F. Bertolucci, I. A. Bertram, C. Bertsche, D. Bertsche, G. J. Besjes, O. Bessidskaia Bylund, M. Bessner, N. Besson, C. Betancourt, S. Bethke, A. J. Bevan, W. Bhimji, R. M. Bianchi, L. Bianchini, M. Bianco, O. Biebel, D. Biedermann, R. Bielski, N. V. Biesuz, M. Biglietti, J. Bilbao De Mendizabal, H. Bilokon, M. Bindi, S. Binet, A. Bingul, C. Bini, S. Biondi, D. M. Bjergaard, C. W. Black, J. E. Black, K. M. Black, D. Blackburn, R. E. Blair, J. -B. Blanchard, J. E. Blanco, T. Blazek, I. Bloch, C. Blocker, W. Blum, U. Blumenschein, S. Blunier, G. J. Bobbink, V. S. Bobrovnikov, S. S. Bocchetta, A. Bocci, C. Bock, M. Boehler, D. Boerner, J. A. Bogaerts, D. Bogavac, A. G. Bogdanchikov, C. Bohm, V. Boisvert, T. Bold, V. Boldea, A. S. Boldyrev, M. Bomben, M. Bona, M. Boonekamp, A. Borisov, G. Borissov, J. Bortfeldt, D. Bortoletto, V. Bortolotto, K. Bos, D. Boscherini, M. Bosman, J. D. Bossio Sola, J. Boudreau, J. Bouffard, E. V. Bouhova-Thacker, D. Boumediene, C. Bourdarios, S. K. Boutle, A. Boveia, J. Boyd, I. R. Boyko, J. Bracinik, A. Brandt, G. Brandt, O. Brandt, U. Bratzler, B. Brau, J. E. Brau, H. M. Braun, W. D. Breaden Madden, K. Brendlinger, A. J. Brennan, L. Brenner, R. Brenner, S. Bressler, T. M. Bristow, D. Britton, D. Britzger, F. M. Brochu, I. Brock, R. Brock, G. Brooijmans, T. Brooks, W. K. Brooks, J. Brosamer, E. Brost, J. H Broughton, P. A. Bruckman de Renstrom, D. Bruncko, R. Bruneliere, A. Bruni, G. Bruni, BH Brunt, M. Bruschi, N. Bruscino, P. Bryant, L. Bryngemark, T. Buanes, Q. Buat, P. Buchholz, A. G. Buckley, I. A. Budagov, F. Buehrer, M. K. Bugge, O. Bulekov, D. Bullock, H. Burckhart, S. Burdin, C. D. Burgard, B. Burghgrave, K. Burka, S. Burke, I. Burmeister, E. Busato, D. Büscher, V. Büscher, P. Bussey, J. M. Butler, A. I. Butt, C. M. Buttar, J. M. Butterworth, P. Butti, W. Buttinger, A. Buzatu, A. R. Buzykaev, S. Cabrera Urbán, D. Caforio, V. M. Cairo, O. Cakir, N. Calace, P. Calafiura, A. Calandri, G. Calderini, P. Calfayan, L. P. Caloba, D. Calvet, S. Calvet, T. P. Calvet, R. Camacho Toro, S. Camarda, P. Camarri, D. Cameron, R. Caminal Armadans, C. Camincher, S. Campana, M. Campanelli, A. Campoverde, V. Canale, A. Canepa, M. Cano Bret, J. Cantero, R. Cantrill, T. Cao, M. D. M. Capeans Garrido, I. Caprini, M. Caprini, M. Capua, R. Caputo, R. M. Carbone, R. Cardarelli, F. Cardillo, I. Carli, T. Carli, G. Carlino, L. Carminati, S. Caron, E. Carquin, G. D. Carrillo-Montoya, J. R. Carter, J. Carvalho, D. Casadei, M. P. Casado, M. Casolino, D. W. Casper, E. Castaneda-Miranda, A. Castelli, V. Castillo Gimenez, N. F. Castro, A. Catinaccio, J. R. Catmore, A. Cattai, J. Caudron, V. Cavaliere, E. Cavallaro, D. Cavalli, M. Cavalli-Sforza, V. Cavasinni, F. Ceradini, L. Cerda Alberich, B. C. Cerio, A. S. Cerqueira, A. Cerri, L. Cerrito, F. Cerutti, M. Cerv, A. Cervelli, S. A. Cetin, A. Chafaq, D. Chakraborty, S. K. Chan, Y. L. Chan, P. Chang, J. D. Chapman, D. G. Charlton, A. Chatterjee, C. C. Chau, C. A. Chavez Barajas, S. Che, S. Cheatham, A. Chegwidden, S. Chekanov, S. V. Chekulaev, G. A. Chelkov, M. A. Chelstowska, C. Chen, H. Chen, K. Chen, S. Chen, S. Chen, X. Chen, Y. Chen, H. C. Cheng, H. J Cheng, Y. Cheng, A. Cheplakov, E. Cheremushkina, R. Cherkaoui El Moursli, V. Chernyatin, E. Cheu, L. Chevalier, V. Chiarella, G. Chiarelli, G. Chiodini, A. S. Chisholm, A. Chitan, M. V. Chizhov, K. Choi, A. R. Chomont, S. Chouridou, B. K. B. Chow, V. Christodoulou, D. Chromek-Burckhart, J. Chudoba, A. J. Chuinard, J. J. Chwastowski, L. Chytka, G. Ciapetti, A. K. Ciftci, D. Cinca, V. Cindro, I. A. Cioara, A. Ciocio, F. Cirotto, Z. H. Citron, M. Ciubancan, A. Clark, B. L. Clark, M. R. Clark, P. J. Clark, R. N. Clarke, C. Clement, Y. Coadou, M. Cobal, A. Coccaro, J. Cochran, L. Coffey, L. Colasurdo, B. Cole, S. Cole, A. P. Colijn, J. Collot, T. Colombo, G. Compostella, P. Conde Muiño, E. Coniavitis, S. H. Connell, I. A. Connelly, V. Consorti, S. Constantinescu, C. Conta, G. Conti, F. Conventi, M. Cooke, B. D. Cooper, A. M. Cooper-Sarkar, T. Cornelissen, M. Corradi, F. Corriveau, A. Corso-Radu, A. Cortes-Gonzalez, G. Cortiana, G. Costa, M. J. Costa, D. Costanzo, G. Cottin, G. Cowan, B. E. Cox, K. Cranmer, S. J. Crawley, G. Cree, S. Crépé-Renaudin, F. Crescioli, W. A. Cribbs, M. Crispin Ortuzar, M. Cristinziani, V. Croft, G. Crosetti, T. Cuhadar Donszelmann, J. Cummings, M. Curatolo, J. Cúth, C. Cuthbert, H. Czirr, P. Czodrowski, S. D’Auria, M. D’Onofrio, M. J. Da Cunha Sargedas De Sousa, C. Da Via, W. Dabrowski, T. Dai, O. Dale, F. Dallaire, C. Dallapiccola, M. Dam, J. R. Dandoy, N. P. Dang, A. C. Daniells, N. S. Dann, M. Danninger, M. Dano Hoffmann, V. Dao, G. Darbo, S. Darmora, J. Dassoulas, A. Dattagupta, W. Davey, C. David, T. Davidek, M. Davies, P. Davison, Y. Davygora, E. Dawe, I. Dawson, R. K. Daya-Ishmukhametova, K. De, R. de Asmundis, A. De Benedetti, S. De Castro, S. De Cecco, N. De Groot, P. de Jong, H. De la Torre, F. De Lorenzi, D. De Pedis, A. De Salvo, U. De Sanctis, A. De Santo, J. B. De Vivie De Regie, W. J. Dearnaley, R. Debbe, C. Debenedetti, D. V. Dedovich, I. Deigaard, J. Del Peso, T. Del Prete, D. Delgove, F. Deliot, C. M. Delitzsch, M. Deliyergiyev, A. Dell’Acqua, L. Dell’Asta, M. Dell’Orso, M. Della Pietra, D. della Volpe, M. Delmastro, P. A. Delsart, C. Deluca, D. A. DeMarco, S. Demers, M. Demichev, A. Demilly, S. P. Denisov, D. Denysiuk, D. Derendarz, J. E. Derkaoui, F. Derue, P. Dervan, K. Desch, C. Deterre, K. Dette, P. O. Deviveiros, A. Dewhurst, S. Dhaliwal, A. Di Ciaccio, L. Di Ciaccio, W. K. Di Clemente, C. Di Donato, A. Di Girolamo, B. Di Girolamo, B. Di Micco, R. Di Nardo, A. Di Simone, R. Di Sipio, D. Di Valentino, C. Diaconu, M. Diamond, F. A. Dias, M. A. Diaz, E. B. Diehl, J. Dietrich, S. Diglio, A. Dimitrievska, J. Dingfelder, P. Dita, S. Dita, F. Dittus, F. Djama, T. Djobava, J. I. Djuvsland, M. A. B. do Vale, D. Dobos, M. Dobre, C. Doglioni, T. Dohmae, J. Dolejsi, Z. Dolezal, B. A. Dolgoshein, M. Donadelli, S. Donati, P. Dondero, J. Donini, J. Dopke, A. Doria, M. T. Dova, A. T. Doyle, E. Drechsler, M. Dris, Y. Du, J. Duarte-Campderros, E. Duchovni, G. Duckeck, O. A. Ducu, D. Duda, A. Dudarev, L. Duflot, L. Duguid, M. Dührssen, M. Dunford, H. Duran Yildiz, M. Düren, A. Durglishvili, D. Duschinger, B. Dutta, M. Dyndal, C. Eckardt, K. M. Ecker, R. C. Edgar, W. Edson, N. C. Edwards, T. Eifert, G. Eigen, K. Einsweiler, T. Ekelof, M. El Kacimi, V. Ellajosyula, M. Ellert, S. Elles, F. Ellinghaus, A. A. Elliot, N. Ellis, J. Elmsheuser, M. Elsing, D. Emeliyanov, Y. Enari, O. C. Endner, M. Endo, J. S. Ennis, J. Erdmann, A. Ereditato, G. Ernis, J. Ernst, M. Ernst, S. Errede, E. Ertel, M. Escalier, H. Esch, C. Escobar, B. Esposito, A. I. Etienvre, E. Etzion, H. Evans, A. Ezhilov, F. Fabbri, L. Fabbri, G. Facini, R. M. Fakhrutdinov, S. Falciano, R. J. Falla, J. Faltova, Y. Fang, M. Fanti, A. Farbin, A. Farilla, C. Farina, T. Farooque, S. Farrell, S. M. Farrington, P. Farthouat, F. Fassi, P. Fassnacht, D. Fassouliotis, M. Faucci Giannelli, A. Favareto, W. J. Fawcett, L. Fayard, O. L. Fedin, W. Fedorko, S. Feigl, L. Feligioni, C. Feng, E. J. Feng, H. Feng, A. B. Fenyuk, L. Feremenga, P. Fernandez Martinez, S. Fernandez Perez, J. Ferrando, A. Ferrari, P. Ferrari, R. Ferrari, D. E. Ferreira de Lima, A. Ferrer, D. Ferrere, C. Ferretti, A. Ferretto Parodi, F. Fiedler, A. Filipčič, M. Filipuzzi, F. Filthaut, M. Fincke-Keeler, K. D. Finelli, M. C. N. Fiolhais, L. Fiorini, A. Firan, A. Fischer, C. Fischer, J. Fischer, W. C. Fisher, N. Flaschel, I. Fleck, P. Fleischmann, G. T. Fletcher, G. Fletcher, R. R. M. Fletcher, T. Flick, A. Floderus, L. R. Flores Castillo, M. J. Flowerdew, G. T. Forcolin, A. Formica, A. Forti, A. G. Foster, D. Fournier, H. Fox, S. Fracchia, P. Francavilla, M. Franchini, D. Francis, L. Franconi, M. Franklin, M. Frate, M. Fraternali, D. Freeborn, S. M. Fressard-Batraneanu, F. Friedrich, D. Froidevaux, J. A. Frost, C. Fukunaga, E. Fullana Torregrosa, T. Fusayasu, J. Fuster, C. Gabaldon, O. Gabizon, A. Gabrielli, A. Gabrielli, G. P. Gach, S. Gadatsch, S. Gadomski, G. Gagliardi, L. G. Gagnon, P. Gagnon, C. Galea, B. Galhardo, E. J. Gallas, B. J. Gallop, P. Gallus, G. Galster, K. K. Gan, J. Gao, Y. Gao, Y. S. Gao, F. M. Garay Walls, C. García, J. E. García Navarro, M. Garcia-Sciveres, R. W. Gardner, N. Garelli, V. Garonne, A. Gascon Bravo, C. Gatti, A. Gaudiello, G. Gaudio, B. Gaur, L. Gauthier, I. L. Gavrilenko, C. Gay, G. Gaycken, E. N. Gazis, Z. Gecse, C. N. P. Gee, Ch. Geich-Gimbel, M. P. Geisler, C. Gemme, M. H. Genest, C. Geng, S. Gentile, S. George, D. Gerbaudo, A. Gershon, S. Ghasemi, H. Ghazlane, M. Ghneimat, B. Giacobbe, S. Giagu, P. Giannetti, B. Gibbard, S. M. Gibson, M. Gignac, M. Gilchriese, T. P. S. Gillam, D. Gillberg, G. Gilles, D. M. Gingrich, N. Giokaris, M. P. Giordani, F. M. Giorgi, F. M. Giorgi, P. F. Giraud, P. Giromini, D. Giugni, F. Giuli, C. Giuliani, M. Giulini, B. K. Gjelsten, S. Gkaitatzis, I. Gkialas, E. L. Gkougkousis, L. K. Gladilin, C. Glasman, J. Glatzer, P. C. F. Glaysher, A. Glazov, M. Goblirsch-Kolb, J. Godlewski, S. Goldfarb, T. Golling, D. Golubkov, A. Gomes, R. Gonçalo, J. Goncalves Pinto Firmino Da Costa, L. Gonella, A. Gongadze, S. González de la Hoz, G. Gonzalez Parra, S. Gonzalez-Sevilla, L. Goossens, P. A. Gorbounov, H. A. Gordon, I. Gorelov, B. Gorini, E. Gorini, A. Gorišek, E. Gornicki, A. T. Goshaw, C. Gössling, M. I. Gostkin, C. R. Goudet, D. Goujdami, A. G. Goussiou, N. Govender, E. Gozani, L. Graber, I. Grabowska-Bold, P. O. J. Gradin, P. Grafström, J. Gramling, E. Gramstad, S. Grancagnolo, V. Gratchev, H. M. Gray, E. Graziani, Z. D. Greenwood, C. Grefe, K. Gregersen, I. M. Gregor, P. Grenier, K. Grevtsov, J. Griffiths, A. A. Grillo, K. Grimm, S. Grinstein, Ph. Gris, J. -F. Grivaz, S. Groh, J. P. Grohs, E. Gross, J. Grosse-Knetter, G. C. Grossi, Z. J. Grout, L. Guan, W. Guan, J. Guenther, F. Guescini, D. Guest, O. Gueta, E. Guido, T. Guillemin, S. Guindon, U. Gul, C. Gumpert, J. Guo, Y. Guo, S. Gupta, G. Gustavino, P. Gutierrez, N. G. Gutierrez Ortiz, C. Gutschow, C. Guyot, C. Gwenlan, C. B. Gwilliam, A. Haas, C. Haber, H. K. Hadavand, N. Haddad, A. Hadef, P. Haefner, S. Hageböck, Z. Hajduk, H. Hakobyan, M. Haleem, J. Haley, D. Hall, G. Halladjian, G. D. Hallewell, K. Hamacher, P. Hamal, K. Hamano, A. Hamilton, G. N. Hamity, P. G. Hamnett, L. Han, K. Hanagaki, K. Hanawa, M. Hance, B. Haney, P. Hanke, R. Hanna, J. B. Hansen, J. D. Hansen, M. C. Hansen, P. H. Hansen, K. Hara, A. S. Hard, T. Harenberg, F. Hariri, S. Harkusha, R. D. Harrington, P. F. Harrison, F. Hartjes, M. Hasegawa, Y. Hasegawa, A. Hasib, S. Hassani, S. Haug, R. Hauser, L. Hauswald, M. Havranek, C. M. Hawkes, R. J. Hawkings, A. D. Hawkins, D. Hayden, C. P. Hays, J. M. Hays, H. S. Hayward, S. J. Haywood, S. J. Head, T. Heck, V. Hedberg, L. Heelan, S. Heim, T. Heim, B. Heinemann, J. J. Heinrich, L. Heinrich, C. Heinz, J. Hejbal, L. Helary, S. Hellman, C. Helsens, J. Henderson, R. C. W. Henderson, Y. Heng, S. Henkelmann, A. M. Henriques Correia, S. Henrot-Versille, G. H. Herbert, Y. Hernández Jiménez, G. Herten, R. Hertenberger, L. Hervas, G. G. Hesketh, N. P. Hessey, J. W. Hetherly, R. Hickling, E. Higón-Rodriguez, E. Hill, J. C. Hill, K. H. Hiller, S. J. Hillier, I. Hinchliffe, E. Hines, R. R. Hinman, M. Hirose, D. Hirschbuehl, J. Hobbs, N. Hod, M. C. Hodgkinson, P. Hodgson, A. Hoecker, M. R. Hoeferkamp, F. Hoenig, M. Hohlfeld, D. Hohn, T. R. Holmes, M. Homann, T. M. Hong, B. H. Hooberman, W. H. Hopkins, Y. Horii, A. J. Horton, J-Y. Hostachy, S. Hou, A. Hoummada, J. Howard, J. Howarth, M. Hrabovsky, I. Hristova, J. Hrivnac, T. Hryn’ova, A. Hrynevich, C. Hsu, P. J. Hsu, S. -C. Hsu, D. Hu, Q. Hu, Y. Huang, Z. Hubacek, F. Hubaut, F. Huegging, T. B. Huffman, E. W. Hughes, G. Hughes, M. Huhtinen, T. A. Hülsing, N. Huseynov, J. Huston, J. Huth, G. Iacobucci, G. Iakovidis, I. Ibragimov, L. Iconomidou-Fayard, E. Ideal, Z. Idrissi, P. Iengo, O. Igonkina, T. Iizawa, Y. Ikegami, M. Ikeno, Y. Ilchenko, D. Iliadis, N. Ilic, T. Ince, G. Introzzi, P. Ioannou, M. Iodice, K. Iordanidou, V. Ippolito, A. Irles Quiles, C. Isaksson, M. Ishino, M. Ishitsuka, R. Ishmukhametov, C. Issever, S. Istin, F. Ito, J. M. Iturbe Ponce, R. Iuppa, J. Ivarsson, W. Iwanski, H. Iwasaki, J. M. Izen, V. Izzo, S. Jabbar, B. Jackson, M. Jackson, P. Jackson, V. Jain, K. B. Jakobi, K. Jakobs, S. Jakobsen, T. Jakoubek, D. O. Jamin, D. K. Jana, E. Jansen, R. Jansky, J. Janssen, M. Janus, G. Jarlskog, N. Javadov, T. Javůrek, F. Jeanneau, L. Jeanty, J. Jejelava, G. -Y. Jeng, D. Jennens, P. Jenni, J. Jentzsch, C. Jeske, S. Jézéquel, H. Ji, J. Jia, H. Jiang, Y. Jiang, S. Jiggins, J. Jimenez Pena, S. Jin, A. Jinaru, O. Jinnouchi, P. Johansson, K. A. Johns, W. J. Johnson, K. Jon-And, G. Jones, R. W. L. Jones, S. Jones, T. J. Jones, J. Jongmanns, P. M. Jorge, J. Jovicevic, X. Ju, A. Juste Rozas, M. K. Köhler, A. Kaczmarska, M. Kado, H. Kagan, M. Kagan, S. J. Kahn, E. Kajomovitz, C. W. Kalderon, A. Kaluza, S. Kama, A. Kamenshchikov, N. Kanaya, S. Kaneti, V. A. Kantserov, J. Kanzaki, B. Kaplan, L. S. Kaplan, A. Kapliy, D. Kar, K. Karakostas, A. Karamaoun, N. Karastathis, M. J. Kareem, E. Karentzos, M. Karnevskiy, S. N. Karpov, Z. M. Karpova, K. Karthik, V. Kartvelishvili, A. N. Karyukhin, K. Kasahara, L. Kashif, R. D. Kass, A. Kastanas, Y. Kataoka, C. Kato, A. Katre, J. Katzy, K. Kawagoe, T. Kawamoto, G. Kawamura, S. Kazama, V. F. Kazanin, R. Keeler, R. Kehoe, J. S. Keller, J. J. Kempster, K Kentaro, H. Keoshkerian, O. Kepka, B. P. Kerševan, S. Kersten, R. A. Keyes, F. Khalil-zada, H. Khandanyan, A. Khanov, A. G. Kharlamov, T. J. Khoo, V. Khovanskiy, E. Khramov, J. Khubua, S. Kido, H. Y. Kim, S. H. Kim, Y. K. Kim, N. Kimura, O. M. Kind, B. T. King, M. King, S. B. King, J. Kirk, A. E. Kiryunin, T. Kishimoto, D. Kisielewska, F. Kiss, K. Kiuchi, O. Kivernyk, E. Kladiva, M. H. Klein, M. Klein, U. Klein, K. Kleinknecht, P. Klimek, A. Klimentov, R. Klingenberg, J. A. Klinger, T. Klioutchnikova, E. -E. Kluge, P. Kluit, S. Kluth, J. Knapik, E. Kneringer, E. B. F. G. Knoops, A. Knue, A. Kobayashi, D. Kobayashi, T. Kobayashi, M. Kobel, M. Kocian, P. Kodys, T. Koffas, E. Koffeman, L. A. Kogan, T. Koi, H. Kolanoski, M. Kolb, I. Koletsou, A. A. Komar, Y. Komori, T. Kondo, N. Kondrashova, K. Köneke, A. C. König, T. Kono, R. Konoplich, N. Konstantinidis, R. Kopeliansky, S. Koperny, L. Köpke, A. K. Kopp, K. Korcyl, K. Kordas, A. Korn, A. A. Korol, I. Korolkov, E. V. Korolkova, O. Kortner, S. Kortner, T. Kosek, V. V. Kostyukhin, A. Kotwal, A. Kourkoumeli-Charalampidi, C. Kourkoumelis, V. Kouskoura, A. Koutsman, A. B. Kowalewska, R. Kowalewski, T. Z. Kowalski, W. Kozanecki, A. S. Kozhin, V. A. Kramarenko, G. Kramberger, D. Krasnopevtsev, M. W. Krasny, A. Krasznahorkay, J. K. Kraus, A. Kravchenko, M. Kretz, J. Kretzschmar, K. Kreutzfeldt, P. Krieger, K. Krizka, K. Kroeninger, H. Kroha, J. Kroll, J. Kroseberg, J. Krstic, U. Kruchonak, H. Krüger, N. Krumnack, A. Kruse, M. C. Kruse, M. Kruskal, T. Kubota, H. Kucuk, S. Kuday, J. T. Kuechler, S. Kuehn, A. Kugel, F. Kuger, A. Kuhl, T. Kuhl, V. Kukhtin, R. Kukla, Y. Kulchitsky, S. Kuleshov, M. Kuna, T. Kunigo, A. Kupco, H. Kurashige, Y. A. Kurochkin, V. Kus, E. S. Kuwertz, M. Kuze, J. Kvita, T. Kwan, D. Kyriazopoulos, A. La Rosa, J. L. La Rosa Navarro, L. La Rotonda, C. Lacasta, F. Lacava, J. Lacey, H. Lacker, D. Lacour, V. R. Lacuesta, E. Ladygin, R. Lafaye, B. Laforge, T. Lagouri, S. Lai, S. Lammers, W. Lampl, E. Lançon, U. Landgraf, M. P. J. Landon, V. S. Lang, J. C. Lange, A. J. Lankford, F. Lanni, K. Lantzsch, A. Lanza, S. Laplace, C. Lapoire, J. F. Laporte, T. Lari, F. Lasagni Manghi, M. Lassnig, P. Laurelli, W. Lavrijsen, A. T. Law, P. Laycock, T. Lazovich, M. Lazzaroni, O. Le Dortz, E. Le Guirriec, E. Le Menedeu, E. P. Le Quilleuc, M. LeBlanc, T. LeCompte, F. Ledroit-Guillon, C. A. Lee, S. C. Lee, L. Lee, G. Lefebvre, M. Lefebvre, F. Legger, C. Leggett, A. Lehan, G. Lehmann Miotto, X. Lei, W. A. Leight, A. Leisos, A. G. Leister, M. A. L. Leite, R. Leitner, D. Lellouch, B. Lemmer, K. J. C. Leney, T. Lenz, B. Lenzi, R. Leone, S. Leone, C. Leonidopoulos, S. Leontsinis, G. Lerner, C. Leroy, A. A. J. Lesage, C. G. Lester, M. Levchenko, J. Levêque, D. Levin, L. J. Levinson, M. Levy, A. M. Leyko, M. Leyton, B. Li, H. Li, H. L. Li, L. Li, L. Li, Q. Li, S. Li, X. Li, Y. Li, Z. Liang, H. Liao, B. Liberti, A. Liblong, P. Lichard, K. Lie, J. Liebal, W. Liebig, C. Limbach, A. Limosani, S. C. Lin, T. H. Lin, B. E. Lindquist, E. Lipeles, A. Lipniacka, M. Lisovyi, T. M. Liss, D. Lissauer, A. Lister, A. M. Litke, B. Liu, D. Liu, H. Liu, H. Liu, J. Liu, J. B. Liu, K. Liu, L. Liu, M. Liu, M. Liu, Y. L. Liu, Y. Liu, M. Livan, A. Lleres, J. Llorente Merino, S. L. Lloyd, F. Lo Sterzo, E. Lobodzinska, P. Loch, W. S. Lockman, F. K. Loebinger, A. E. Loevschall-Jensen, K. M. Loew, A. Loginov, T. Lohse, K. Lohwasser, M. Lokajicek, B. A. Long, J. D. Long, R. E. Long, L. Longo, K. A. Looper, L. Lopes, D. Lopez Mateos, B. Lopez Paredes, I. Lopez Paz, A. Lopez Solis, J. Lorenz, N. Lorenzo Martinez, M. Losada, P. J. Lösel, X. Lou, A. Lounis, J. Love, P. A. Love, H. Lu, N. Lu, H. J. Lubatti, C. Luci, A. Lucotte, C. Luedtke, F. Luehring, W. Lukas, L. Luminari, O. Lundberg, B. Lund-Jensen, D. Lynn, R. Lysak, E. Lytken, V. Lyubushkin, H. Ma, L. L. Ma, Y. Ma, G. Maccarrone, A. Macchiolo, C. M. Macdonald, B. Maček, J. Machado Miguens, D. Madaffari, R. Madar, H. J. Maddocks, W. F. Mader, A. Madsen, J. Maeda, S. Maeland, T. Maeno, A. Maevskiy, E. Magradze, J. Mahlstedt, C. Maiani, C. Maidantchik, A. A. Maier, T. Maier, A. Maio, S. Majewski, Y. Makida, N. Makovec, B. Malaescu, Pa. Malecki, V. P. Maleev, F. Malek, U. Mallik, D. Malon, C. Malone, S. Maltezos, S. Malyukov, J. Mamuzic, G. Mancini, B. Mandelli, L. Mandelli, I. Mandić, J. Maneira, L. Manhaes de Andrade Filho, J. Manjarres Ramos, A. Mann, B. Mansoulie, R. Mantifel, M. Mantoani, S. Manzoni, L. Mapelli, G. Marceca, L. March, G. Marchiori, M. Marcisovsky, M. Marjanovic, D. E. Marley, F. Marroquim, S. P. Marsden, Z. Marshall, L. F. Marti, S. Marti-Garcia, B. Martin, T. A. Martin, V. J. Martin, B. Martin dit Latour, M. Martinez, S. Martin-Haugh, V. S. Martoiu, A. C. Martyniuk, M. Marx, F. Marzano, A. Marzin, L. Masetti, T. Mashimo, R. Mashinistov, J. Masik, A. L. Maslennikov, I. Massa, L. Massa, P. Mastrandrea, A. Mastroberardino, T. Masubuchi, P. Mättig, J. Mattmann, J. Maurer, S. J. Maxfield, D. A. Maximov, R. Mazini, S. M. Mazza, N. C. Mc Fadden, G. Mc Goldrick, S. P. Mc Kee, A. McCarn, R. L. McCarthy, T. G. McCarthy, L. I. McClymont, K. W. McFarlane, J. A. Mcfayden, G. Mchedlidze, S. J. McMahon, R. A. McPherson, M. Medinnis, S. Meehan, S. Mehlhase, A. Mehta, K. Meier, C. Meineck, B. Meirose, B. R. Mellado Garcia, F. Meloni, A. Mengarelli, S. Menke, E. Meoni, K. M. Mercurio, S. Mergelmeyer, P. Mermod, L. Merola, C. Meroni, F. S. Merritt, A. Messina, J. Metcalfe, A. S. Mete, C. Meyer, C. Meyer, J-P. Meyer, J. Meyer, H. Meyer Zu Theenhausen, R. P. Middleton, S. Miglioranzi, L. Mijović, G. Mikenberg, M. Mikestikova, M. Mikuž, M. Milesi, A. Milic, D. W. Miller, C. Mills, A. Milov, D. A. Milstead, A. A. Minaenko, Y. Minami, I. A. Minashvili, A. I. Mincer, B. Mindur, M. Mineev, Y. Ming, L. M. Mir, K. P. Mistry, T. Mitani, J. Mitrevski, V. A. Mitsou, A. Miucci, P. S. Miyagawa, J. U. Mjörnmark, T. Moa, K. Mochizuki, S. Mohapatra, W. Mohr, S. Molander, R. Moles-Valls, R. Monden, M. C. Mondragon, K. Mönig, J. Monk, E. Monnier, A. Montalbano, J. Montejo Berlingen, F. Monticelli, S. Monzani, R. W. Moore, N. Morange, D. Moreno, M. Moreno Llácer, P. Morettini, D. Mori, T. Mori, M. Morii, M. Morinaga, V. Morisbak, S. Moritz, A. K. Morley, G. Mornacchi, J. D. Morris, S. S. Mortensen, L. Morvaj, M. Mosidze, J. Moss, K. Motohashi, R. Mount, E. Mountricha, S. V. Mouraviev, E. J. W. Moyse, S. Muanza, R. D. Mudd, F. Mueller, J. Mueller, R. S. P. Mueller, T. Mueller, D. Muenstermann, P. Mullen, G. A. Mullier, F. J. Munoz Sanchez, J. A. Murillo Quijada, W. J. Murray, H. Musheghyan, M. Muškinja, A. G. Myagkov, M. Myska, B. P. Nachman, O. Nackenhorst, J. Nadal, K. Nagai, R. Nagai, K. Nagano, Y. Nagasaka, K. Nagata, M. Nagel, E. Nagy, A. M. Nairz, Y. Nakahama, K. Nakamura, T. Nakamura, I. Nakano, H. Namasivayam, R. F. Naranjo Garcia, R. Narayan, D. I. Narrias Villar, I. Naryshkin, T. Naumann, G. Navarro, R. Nayyar, H. A. Neal, P. Yu. Nechaeva, T. J. Neep, P. D. Nef, A. Negri, M. Negrini, S. Nektarijevic, C. Nellist, A. Nelson, S. Nemecek, P. Nemethy, A. A. Nepomuceno, M. Nessi, M. S. Neubauer, M. Neumann, R. M. Neves, P. Nevski, P. R. Newman, D. H. Nguyen, R. B. Nickerson, R. Nicolaidou, B. Nicquevert, J. Nielsen, A. Nikiforov, V. Nikolaenko, I. Nikolic-Audit, K. Nikolopoulos, J. K. Nilsen, P. Nilsson, Y. Ninomiya, A. Nisati, R. Nisius, T. Nobe, L. Nodulman, M. Nomachi, I. Nomidis, T. Nooney, S. Norberg, M. Nordberg, N. Norjoharuddeen, O. Novgorodova, S. Nowak, M. Nozaki, L. Nozka, K. Ntekas, E. Nurse, F. Nuti, F. O’grady, D. C. O’Neil, A. A. O’Rourke, V. O’Shea, F. G. Oakham, H. Oberlack, T. Obermann, J. Ocariz, A. Ochi, I. Ochoa, J. P. Ochoa-Ricoux, S. Oda, S. Odaka, H. Ogren, A. Oh, S. H. Oh, C. C. Ohm, H. Ohman, H. Oide, H. Okawa, Y. Okumura, T. Okuyama, A. Olariu, L. F. Oleiro Seabra, S. A. Olivares Pino, D. Oliveira Damazio, A. Olszewski, J. Olszowska, A. Onofre, K. Onogi, P. U. E. Onyisi, C. J. Oram, M. J. Oreglia, Y. Oren, D. Orestano, N. Orlando, R. S. Orr, B. Osculati, R. Ospanov, G. Otero y Garzon, H. Otono, M. Ouchrif, F. Ould-Saada, A. Ouraou, K. P. Oussoren, Q. Ouyang, A. Ovcharova, M. Owen, R. E. Owen, V. E. Ozcan, N. Ozturk, K. Pachal, A. Pacheco Pages, C. Padilla Aranda, M. Pagáčová, S. Pagan Griso, F. Paige, P. Pais, K. Pajchel, G. Palacino, S. Palestini, M. Palka, D. Pallin, A. Palma, E. St. Panagiotopoulou, C. E. Pandini, J. G. Panduro Vazquez, P. Pani, S. Panitkin, D. Pantea, L. Paolozzi, Th. D. Papadopoulou, K. Papageorgiou, A. Paramonov, D. Paredes Hernandez, A. J. Parker, M. A. Parker, K. A. Parker, F. Parodi, J. A. Parsons, U. Parzefall, V. R. Pascuzzi, E. Pasqualucci, S. Passaggio, F. Pastore, Fr. Pastore, G. Pásztor, S. Pataraia, N. D. Patel, J. R. Pater, T. Pauly, J. Pearce, B. Pearson, L. E. Pedersen, M. Pedersen, S. Pedraza Lopez, R. Pedro, S. V. Peleganchuk, D. Pelikan, O. Penc, C. Peng, H. Peng, J. Penwell, B. S. Peralva, M. M. Perego, D. V. Perepelitsa, E. Perez Codina, L. Perini, H. Pernegger, S. Perrella, R. Peschke, V. D. Peshekhonov, K. Peters, R. F. Y. Peters, B. A. Petersen, T. C. Petersen, E. Petit, A. Petridis, C. Petridou, P. Petroff, E. Petrolo, M. Petrov, F. Petrucci, N. E. Pettersson, A. Peyaud, R. Pezoa, P. W. Phillips, G. Piacquadio, E. Pianori, A. Picazio, E. Piccaro, M. Piccinini, M. A. Pickering, R. Piegaia, J. E. Pilcher, A. D. Pilkington, A. W. J. Pin, J. Pina, M. Pinamonti, J. L. Pinfold, A. Pingel, S. Pires, H. Pirumov, M. Pitt, L. Plazak, M. -A. Pleier, V. Pleskot, E. Plotnikova, P. Plucinski, D. Pluth, R. Poettgen, L. Poggioli, D. Pohl, G. Polesello, A. Poley, A. Policicchio, R. Polifka, A. Polini, C. S. Pollard, V. Polychronakos, K. Pommès, L. Pontecorvo, B. G. Pope, G. A. Popeneciu, D. S. Popovic, A. Poppleton, S. Pospisil, K. Potamianos, I. N. Potrap, C. J. Potter, C. T. Potter, G. Poulard, J. Poveda, V. Pozdnyakov, M. E. Pozo Astigarraga, P. Pralavorio, A. Pranko, S. Prell, D. Price, L. E. Price, M. Primavera, S. Prince, M. Proissl, K. Prokofiev, F. Prokoshin, S. Protopopescu, J. Proudfoot, M. Przybycien, D. Puddu, D. Puldon, M. Purohit, P. Puzo, J. Qian, G. Qin, Y. Qin, A. Quadt, W. B. Quayle, M. Queitsch-Maitland, D. Quilty, S. Raddum, V. Radeka, V. Radescu, S. K. Radhakrishnan, P. Radloff, P. Rados, F. Ragusa, G. Rahal, J. A. Raine, S. Rajagopalan, M. Rammensee, C. Rangel-Smith, M. G. Ratti, F. Rauscher, S. Rave, T. Ravenscroft, M. Raymond, A. L. Read, N. P. Readioff, D. M. Rebuzzi, A. Redelbach, G. Redlinger, R. Reece, K. Reeves, L. Rehnisch, J. Reichert, H. Reisin, C. Rembser, H. Ren, M. Rescigno, S. Resconi, O. L. Rezanova, P. Reznicek, R. Rezvani, R. Richter, S. Richter, E. Richter-Was, O. Ricken, M. Ridel, P. Rieck, C. J. Riegel, J. Rieger, O. Rifki, M. Rijssenbeek, A. Rimoldi, L. Rinaldi, B. Ristić, E. Ritsch, I. Riu, F. Rizatdinova, E. Rizvi, C. Rizzi, S. H. Robertson, A. Robichaud-Veronneau, D. Robinson, J. E. M. Robinson, A. Robson, C. Roda, Y. Rodina, A. Rodriguez Perez, D. Rodriguez Rodriguez, S. Roe, C. S. Rogan, O. Røhne, A. Romaniouk, M. Romano, S. M. Romano Saez, E. Romero Adam, N. Rompotis, M. Ronzani, L. Roos, E. Ros, S. Rosati, K. Rosbach, P. Rose, O. Rosenthal, V. Rossetti, E. Rossi, L. P. Rossi, J. H. N. Rosten, R. Rosten, M. Rotaru, I. Roth, J. Rothberg, D. Rousseau, C. R. Royon, A. Rozanov, Y. Rozen, X. Ruan, F. Rubbo, I. Rubinskiy, V. I. Rud, M. S. Rudolph, F. Rühr, A. Ruiz-Martinez, Z. Rurikova, N. A. Rusakovich, A. Ruschke, H. L. Russell, J. P. Rutherfoord, N. Ruthmann, Y. F. Ryabov, M. Rybar, G. Rybkin, S. Ryu, A. Ryzhov, A. F. Saavedra, G. Sabato, S. Sacerdoti, H. F-W. Sadrozinski, R. Sadykov, F. Safai Tehrani, P. Saha, M. Sahinsoy, M. Saimpert, T. Saito, H. Sakamoto, Y. Sakurai, G. Salamanna, A. Salamon, J. E. Salazar Loyola, D. Salek, P. H. Sales De Bruin, D. Salihagic, A. Salnikov, J. Salt, D. Salvatore, F. Salvatore, A. Salvucci, A. Salzburger, D. Sammel, D. Sampsonidis, A. Sanchez, J. Sánchez, V. Sanchez Martinez, H. Sandaker, R. L. Sandbach, H. G. Sander, M. P. Sanders, M. Sandhoff, C. Sandoval, R. Sandstroem, D. P. C. Sankey, M. Sannino, A. Sansoni, C. Santoni, R. Santonico, H. Santos, I. Santoyo Castillo, K. Sapp, A. Sapronov, J. G. Saraiva, B. Sarrazin, O. Sasaki, Y. Sasaki, K. Sato, G. Sauvage, E. Sauvan, G. Savage, P. Savard, C. Sawyer, L. Sawyer, J. Saxon, C. Sbarra, A. Sbrizzi, T. Scanlon, D. A. Scannicchio, M. Scarcella, V. Scarfone, J. Schaarschmidt, P. Schacht, D. Schaefer, R. Schaefer, J. Schaeffer, S. Schaepe, S. Schaetzel, U. Schäfer, A. C. Schaffer, D. Schaile, R. D. Schamberger, V. Scharf, V. A. Schegelsky, D. Scheirich, M. Schernau, C. Schiavi, C. Schillo, M. Schioppa, S. Schlenker, K. Schmieden, C. Schmitt, S. Schmitt, S. Schmitz, B. Schneider, Y. J. Schnellbach, U. Schnoor, L. Schoeffel, A. Schoening, B. D. Schoenrock, E. Schopf, A. L. S. Schorlemmer, M. Schott, J. Schovancova, S. Schramm, M. Schreyer, N. Schuh, M. J. Schultens, H. -C. Schultz-Coulon, H. Schulz, M. Schumacher, B. A. Schumm, Ph. Schune, C. Schwanenberger, A. Schwartzman, T. A. Schwarz, Ph. Schwegler, H. Schweiger, Ph. Schwemling, R. Schwienhorst, J. Schwindling, T. Schwindt, G. Sciolla, F. Scuri, F. Scutti, J. Searcy, P. Seema, S. C. Seidel, A. Seiden, F. Seifert, J. M. Seixas, G. Sekhniaidze, K. Sekhon, S. J. Sekula, D. M. Seliverstov, N. Semprini-Cesari, C. Serfon, L. Serin, L. Serkin, M. Sessa, R. Seuster, H. Severini, T. Sfiligoj, F. Sforza, A. Sfyrla, E. Shabalina, N. W. Shaikh, L. Y. Shan, R. Shang, J. T. Shank, M. Shapiro, P. B. Shatalov, K. Shaw, S. M. Shaw, A. Shcherbakova, C. Y. Shehu, P. Sherwood, L. Shi, S. Shimizu, C. O. Shimmin, M. Shimojima, M. Shiyakova, A. Shmeleva, D. Shoaleh Saadi, M. J. Shochet, S. Shojaii, S. Shrestha, E. Shulga, M. A. Shupe, P. Sicho, P. E. Sidebo, O. Sidiropoulou, D. Sidorov, A. Sidoti, F. Siegert, Dj. Sijacki, J. Silva, S. B. Silverstein, V. Simak, O. Simard, Lj. Simic, S. Simion, E. Simioni, B. Simmons, D. Simon, M. Simon, P. Sinervo, N. B. Sinev, M. Sioli, G. Siragusa, S. Yu. Sivoklokov, J. Sjölin, T. B. Sjursen, M. B. Skinner, H. P. Skottowe, P. Skubic, M. Slater, T. Slavicek, M. Slawinska, K. Sliwa, R. Slovak, V. Smakhtin, B. H. Smart, L. Smestad, S. Yu. Smirnov, Y. Smirnov, L. N. Smirnova, O. Smirnova, M. N. K. Smith, R. W. Smith, M. Smizanska, K. Smolek, A. A. Snesarev, G. Snidero, S. Snyder, R. Sobie, F. Socher, A. Soffer, D. A. Soh, G. Sokhrannyi, C. A. Solans Sanchez, M. Solar, E. Yu. Soldatov, U. Soldevila, A. A. Solodkov, A. Soloshenko, O. V. Solovyanov, V. Solovyev, P. Sommer, H. Son, H. Y. Song, A. Sood, A. Sopczak, V. Sopko, V. Sorin, D. Sosa, C. L. Sotiropoulou, R. Soualah, A. M. Soukharev, D. South, B. C. Sowden, S. Spagnolo, M. Spalla, M. Spangenberg, F. Spanò, D. Sperlich, F. Spettel, R. Spighi, G. Spigo, L. A. Spiller, M. Spousta, R. D. St. Denis, A. Stabile, J. Stahlman, R. Stamen, S. Stamm, E. Stanecka, R. W. Stanek, C. Stanescu, M. Stanescu-Bellu, M. M. Stanitzki, S. Stapnes, E. A. Starchenko, G. H. Stark, J. Stark, P. Staroba, P. Starovoitov, S. Stärz, R. Staszewski, P. Steinberg, B. Stelzer, H. J. Stelzer, O. Stelzer-Chilton, H. Stenzel, G. A. Stewart, J. A. Stillings, M. C. Stockton, M. Stoebe, G. Stoicea, P. Stolte, S. Stonjek, A. R. Stradling, A. Straessner, M. E. Stramaglia, J. Strandberg, S. Strandberg, A. Strandlie, M. Strauss, P. Strizenec, R. Ströhmer, D. M. Strom, R. Stroynowski, A. Strubig, S. A. Stucci, B. Stugu, N. A. Styles, D. Su, J. Su, R. Subramaniam, S. Suchek, Y. Sugaya, M. Suk, V. V. Sulin, S. Sultansoy, T. Sumida, S. Sun, X. Sun, J. E. Sundermann, K. Suruliz, G. Susinno, M. R. Sutton, S. Suzuki, M. Svatos, M. Swiatlowski, I. Sykora, T. Sykora, D. Ta, C. Taccini, K. Tackmann, J. Taenzer, A. Taffard, R. Tafirout, N. Taiblum, H. Takai, R. Takashima, H. Takeda, T. Takeshita, Y. Takubo, M. Talby, A. A. Talyshev, J. Y. C. Tam, K. G. Tan, J. Tanaka, R. Tanaka, S. Tanaka, B. B. Tannenwald, S. Tapia Araya, S. Tapprogge, S. Tarem, G. F. Tartarelli, P. Tas, M. Tasevsky, T. Tashiro, E. Tassi, A. Tavares Delgado, Y. Tayalati, A. C. Taylor, G. N. Taylor, P. T. E. Taylor, W. Taylor, F. A. Teischinger, P. Teixeira-Dias, K. K. Temming, D. Temple, H. Ten Kate, P. K. Teng, J. J. Teoh, F. Tepel, S. Terada, K. Terashi, J. Terron, S. Terzo, M. Testa, R. J. Teuscher, T. Theveneaux-Pelzer, J. P. Thomas, J. Thomas-Wilsker, E. N. Thompson, P. D. Thompson, R. J. Thompson, A. S. Thompson, L. A. Thomsen, E. Thomson, M. Thomson, M. J. Tibbetts, R. E. Ticse Torres, V. O. Tikhomirov, Yu. A. Tikhonov, S. Timoshenko, P. Tipton, S. Tisserant, K. Todome, T. Todorov, S. Todorova-Nova, J. Tojo, S. Tokár, K. Tokushuku, E. Tolley, L. Tomlinson, M. Tomoto, L. Tompkins, K. Toms, B. Tong, E. Torrence, H. Torres, E. Torró Pastor, J. Toth, F. Touchard, D. R. Tovey, T. Trefzger, A. Tricoli, I. M. Trigger, S. Trincaz-Duvoid, M. F. Tripiana, W. Trischuk, B. Trocmé, A. Trofymov, C. Troncon, M. Trottier-McDonald, M. Trovatelli, L. Truong, M. Trzebinski, A. Trzupek, J. C-L. Tseng, P. V. Tsiareshka, G. Tsipolitis, N. Tsirintanis, S. Tsiskaridze, V. Tsiskaridze, E. G. Tskhadadze, K. M. Tsui, I. I. Tsukerman, V. Tsulaia, S. Tsuno, D. Tsybychev, A. Tudorache, V. Tudorache, A. N. Tuna, S. A. Tupputi, S. Turchikhin, D. Turecek, D. Turgeman, R. Turra, A. J. Turvey, P. M. Tuts, M. Tyndel, G. Ucchielli, I. Ueda, R. Ueno, M. Ughetto, F. Ukegawa, G. Unal, A. Undrus, G. Unel, F. C. Ungaro, Y. Unno, C. Unverdorben, J. Urban, P. Urquijo, P. Urrejola, G. Usai, A. Usanova, L. Vacavant, V. Vacek, B. Vachon, C. Valderanis, E. Valdes Santurio, N. Valencic, S. Valentinetti, A. Valero, L. Valery, S. Valkar, S. Vallecorsa, J. A. Valls Ferrer, W. Van Den Wollenberg, P. C. Van Der Deijl, R. van der Geer, H. van der Graaf, N. van Eldik, P. van Gemmeren, J. Van Nieuwkoop, I. van Vulpen, M. C. van Woerden, M. Vanadia, W. Vandelli, R. Vanguri, A. Vaniachine, P. Vankov, G. Vardanyan, R. Vari, E. W. Varnes, T. Varol, D. Varouchas, A. Vartapetian, K. E. Varvell, J. G. Vasquez, F. Vazeille, T. Vazquez Schroeder, J. Veatch, L. M. Veloce, F. Veloso, S. Veneziano, A. Ventura, M. Venturi, N. Venturi, A. Venturini, V. Vercesi, M. Verducci, W. Verkerke, J. C. Vermeulen, A. Vest, M. C. Vetterli, O. Viazlo, I. Vichou, T. Vickey, O. E. Vickey Boeriu, G. H. A. Viehhauser, S. Viel, L. Vigani, R. Vigne, M. Villa, M. Villaplana Perez, E. Vilucchi, M. G. Vincter, V. B. Vinogradov, C. Vittori, I. Vivarelli, S. Vlachos, M. Vlasak, M. Vogel, P. Vokac, G. Volpi, M. Volpi, H. von der Schmitt, E. von Toerne, V. Vorobel, K. Vorobev, M. Vos, R. Voss, J. H. Vossebeld, N. Vranjes, M. Vranjes Milosavljevic, V. Vrba, M. Vreeswijk, R. Vuillermet, I. Vukotic, Z. Vykydal, P. Wagner, W. Wagner, H. Wahlberg, S. Wahrmund, J. Wakabayashi, J. Walder, R. Walker, W. Walkowiak, V. Wallangen, C. Wang, C. Wang, F. Wang, H. Wang, H. Wang, J. Wang, J. Wang, K. Wang, R. Wang, S. M. Wang, T. Wang, T. Wang, X. Wang, C. Wanotayaroj, A. Warburton, C. P. Ward, D. R. Wardrope, A. Washbrook, P. M. Watkins, A. T. Watson, I. J. Watson, M. F. Watson, G. Watts, S. Watts, B. M. Waugh, S. Webb, M. S. Weber, S. W. Weber, J. S. Webster, A. R. Weidberg, B. Weinert, J. Weingarten, C. Weiser, H. Weits, P. S. Wells, T. Wenaus, T. Wengler, S. Wenig, N. Wermes, M. Werner, P. Werner, M. Wessels, J. Wetter, K. Whalen, N. L. Whallon, A. M. Wharton, A. White, M. J. White, R. White, S. White, D. Whiteson, F. J. Wickens, W. Wiedenmann, M. Wielers, P. Wienemann, C. Wiglesworth, L. A. M. Wiik-Fuchs, A. Wildauer, F. Wilk, H. G. Wilkens, H. H. Williams, S. Williams, C. Willis, S. Willocq, J. A. Wilson, I. Wingerter-Seez, F. Winklmeier, O. J. Winston, B. T. Winter, M. Wittgen, J. Wittkowski, S. J. Wollstadt, M. W. Wolter, H. Wolters, B. K. Wosiek, J. Wotschack, M. J. Woudstra, K. W. Wozniak, M. Wu, M. Wu, S. L. Wu, X. Wu, Y. Wu, T. R. Wyatt, B. M. Wynne, S. Xella, D. Xu, L. Xu, B. Yabsley, S. Yacoob, R. Yakabe, D. Yamaguchi, Y. Yamaguchi, A. Yamamoto, S. Yamamoto, T. Yamanaka, K. Yamauchi, Y. Yamazaki, Z. Yan, H. Yang, H. Yang, Y. Yang, Z. Yang, W-M. Yao, Y. C. Yap, Y. Yasu, E. Yatsenko, K. H. Yau Wong, J. Ye, S. Ye, I. Yeletskikh, A. L. Yen, E. Yildirim, K. Yorita, R. Yoshida, K. Yoshihara, C. Young, C. J. S. Young, S. Youssef, D. R. Yu, J. Yu, J. M. Yu, J. Yu, L. Yuan, S. P. Y. Yuen, I. Yusuff, B. Zabinski, R. Zaidan, A. M. Zaitsev, N. Zakharchuk, J. Zalieckas, A. Zaman, S. Zambito, L. Zanello, D. Zanzi, C. Zeitnitz, M. Zeman, A. Zemla, J. C. Zeng, Q. Zeng, K. Zengel, O. Zenin, T. Ženiš, D. Zerwas, D. Zhang, F. Zhang, G. Zhang, H. Zhang, J. Zhang, L. Zhang, R. Zhang, R. Zhang, X. Zhang, Z. Zhang, X. Zhao, Y. Zhao, Z. Zhao, A. Zhemchugov, J. Zhong, B. Zhou, C. Zhou, L. Zhou, L. Zhou, M. Zhou, N. Zhou, C. G. Zhu, H. Zhu, J. Zhu, Y. Zhu, X. Zhuang, K. Zhukov, A. Zibell, D. Zieminska, N. I. Zimine, C. Zimmermann, S. Zimmermann, Z. Zinonos, M. Zinser, M. Ziolkowski, L. Živković, G. Zobernig, A. Zoccoli, M. zur Nedden, G. Zurzolo, L. Zwalinski, ATLAS Collaboration

**Affiliations:** 1 Department of Physics, University of Adelaide, Adelaide, Australia; 2 Physics Department, SUNY Albany, Albany, NY USA; 3 Department of Physics, University of Alberta, Edmonton, AB Canada; 4 Department of Physics, Ankara University, Ankara, Turkey; 5 Istanbul Aydin University, Istanbul, Turkey; 6 Division of Physics, TOBB University of Economics and Technology, Ankara, Turkey; 7 LAPP, CNRS/IN2P3 and Université Savoie Mont Blanc, Annecy-le-Vieux, France; 8 High Energy Physics Division, Argonne National Laboratory, Argonne, IL USA; 9 Department of Physics, University of Arizona, Tucson, AZ USA; 10 Department of Physics, The University of Texas at Arlington, Arlington, TX USA; 11 Physics Department, University of Athens, Athens, Greece; 12 Physics Department, National Technical University of Athens, Zografou, Greece; 13 Department of Physics, The University of Texas at Austin, Austin, TX USA; 14 Institute of Physics, Azerbaijan Academy of Sciences, Baku, Azerbaijan; 15 Institut de Física d’Altes Energies (IFAE), The Barcelona Institute of Science and Technology, Barcelona, Spain; 16 Institute of Physics, University of Belgrade, Belgrade, Serbia; 17 Department for Physics and Technology, University of Bergen, Bergen, Norway; 18 Physics Division, Lawrence Berkeley National Laboratory and University of California, Berkeley, CA USA; 19 Department of Physics, Humboldt University, Berlin, Germany; 20 Albert Einstein Center for Fundamental Physics and Laboratory for High Energy Physics, University of Bern, Bern, Switzerland; 21 School of Physics and Astronomy, University of Birmingham, Birmingham, UK; 22 Department of Physics, Bogazici University, Istanbul, Turkey; 23 Department of Physics Engineering, Gaziantep University, Gaziantep, Turkey; 24 Faculty of Engineering and Natural Sciences, Istanbul Bilgi University, Istanbul, Turkey; 25 Faculty of Engineering and Natural Sciences, Bahcesehir University, Istanbul, Turkey; 26 Centro de Investigaciones, Universidad Antonio Narino, Bogota, Colombia; 27 INFN Sezione di Bologna, Bologna, Italy; 28 Dipartimento di Fisica e Astronomia, Università di Bologna, Bologna, Italy; 29 Physikalisches Institut, University of Bonn, Bonn, Germany; 30 Department of Physics, Boston University, Boston, MA USA; 31 Department of Physics, Brandeis University, Waltham, MA USA; 32 Universidade Federal do Rio De Janeiro COPPE/EE/IF, Rio de Janeiro, Brazil; 33 Electrical Circuits Department, Federal University of Juiz de Fora (UFJF), Juiz de Fora, Brazil; 34 Federal University of Sao Joao del Rei (UFSJ), Sao Joao del Rei, Brazil; 35 Instituto de Fisica, Universidade de Sao Paulo, São Paulo, Brazil; 36 Physics Department, Brookhaven National Laboratory, Upton, NY USA; 37 Transilvania University of Brasov, Brasov, Romania; 38 National Institute of Physics and Nuclear Engineering, Bucharest, Romania; 39 Physics Department, National Institute for Research and Development of Isotopic and Molecular Technologies, Cluj Napoca, Romania; 40 University Politehnica Bucharest, Bucharest, Romania; 41 West University in Timisoara, Timisoara, Romania; 42 Departamento de Física, Universidad de Buenos Aires, Buenos Aires, Argentina; 43 Cavendish Laboratory, University of Cambridge, Cambridge, UK; 44 Department of Physics, Carleton University, Ottawa, ON Canada; 45 CERN, Geneva, Switzerland; 46 Enrico Fermi Institute, University of Chicago, Chicago, IL USA; 47 Departamento de Física, Pontificia Universidad Católica de Chile, Santiago, Chile; 48 Departamento de Física, Universidad Técnica Federico Santa María, Valparaiso, Chile; 49 Institute of High Energy Physics, Chinese Academy of Sciences, Beijing, China; 50 Department of Modern Physics, University of Science and Technology of China, Hefei, Anhui China; 51 Department of Physics, Nanjing University, Nanjing, Jiangsu China; 52 School of Physics, Shandong University, Jinan, Shandong China; 53 Shanghai Key Laboratory for Particle Physics and Cosmology, Department of Physics and Astronomy, Shanghai Jiao Tong University, (also affiliated with PKU-CHEP), Shanghai, China; 54 Physics Department, Tsinghua University, Beijing, 100084 China; 55 Laboratoire de Physique Corpusculaire, Clermont Université and Université Blaise Pascal and CNRS/IN2P3, Clermont-Ferrand, France; 56 Nevis Laboratory, Columbia University, Irvington, NY USA; 57 Niels Bohr Institute, University of Copenhagen, Kobenhavn, Denmark; 58 Laboratori Nazionali di Frascati, INFN Gruppo Collegato di Cosenza, Frascati, Italy; 59 Dipartimento di Fisica, Università della Calabria, Rende, Italy; 60 Faculty of Physics and Applied Computer Science, AGH University of Science and Technology, Kraków, Poland; 61 Marian Smoluchowski Institute of Physics, Jagiellonian University, Kraków, Poland; 62 Institute of Nuclear Physics, Polish Academy of Sciences, Kraków, Poland; 63 Physics Department, Southern Methodist University, Dallas, TX USA; 64 Physics Department, University of Texas at Dallas, Richardson, TX USA; 65 DESY, Hamburg and Zeuthen, Germany; 66 Institut für Experimentelle Physik IV, Technische Universität Dortmund, Dortmund, Germany; 67 Institut für Kern- und Teilchenphysik, Technische Universität Dresden, Dresden, Germany; 68 Department of Physics, Duke University, Durham, NC USA; 69 SUPA-School of Physics and Astronomy, University of Edinburgh, Edinburgh, UK; 70 INFN Laboratori Nazionali di Frascati, Frascati, Italy; 71 Fakultät für Mathematik und Physik, Albert-Ludwigs-Universität, Freiburg, Germany; 72 Section de Physique, Université de Genève, Geneva, Switzerland; 73 INFN Sezione di Genova, Genoa, Italy; 74 Dipartimento di Fisica, Università di Genova, Genoa, Italy; 75 E. Andronikashvili Institute of Physics, Iv. Javakhishvili Tbilisi State University, Tbilisi, Georgia; 76 High Energy Physics Institute, Tbilisi State University, Tbilisi, Georgia; 77 II Physikalisches Institut, Justus-Liebig-Universität Giessen, Giessen, Germany; 78 SUPA-School of Physics and Astronomy, University of Glasgow, Glasgow, UK; 79 II Physikalisches Institut, Georg-August-Universität, Göttingen, Germany; 80 Laboratoire de Physique Subatomique et de Cosmologie, Université Grenoble-Alpes, CNRS/IN2P3, Grenoble, France; 81 Department of Physics, Hampton University, Hampton, VA USA; 82 Laboratory for Particle Physics and Cosmology, Harvard University, Cambridge, MA USA; 83 Kirchhoff-Institut für Physik, Ruprecht-Karls-Universität Heidelberg, Heidelberg, Germany; 84 Physikalisches Institut, Ruprecht-Karls-Universität Heidelberg, Heidelberg, Germany; 85 ZITI Institut für technische Informatik, Ruprecht-Karls-Universität Heidelberg, Mannheim, Germany; 86 Faculty of Applied Information Science, Hiroshima Institute of Technology, Hiroshima, Japan; 87 Department of Physics, The Chinese University of Hong Kong, Shatin, N.T., Hong Kong, China; 88 Department of Physics, The University of Hong Kong, Hong Kong, China; 89 Department of Physics, The Hong Kong University of Science and Technology, Clear Water Bay, Kowloon, Hong Kong China; 90 Department of Physics, Indiana University, Bloomington, IN USA; 91 Institut für Astro- und Teilchenphysik, Leopold-Franzens-Universität, Innsbruck, Austria; 92 University of Iowa, Iowa City, IA USA; 93 Department of Physics and Astronomy, Iowa State University, Ames, IA USA; 94 Joint Institute for Nuclear Research, JINR Dubna, Dubna, Russia; 95 KEK, High Energy Accelerator Research Organization, Tsukuba, Japan; 96 Graduate School of Science, Kobe University, Kobe, Japan; 97 Faculty of Science, Kyoto University, Kyoto, Japan; 98 Kyoto University of Education, Kyoto, Japan; 99 Department of Physics, Kyushu University, Fukuoka, Japan; 100 Instituto de Física La Plata, Universidad Nacional de La Plata and CONICET, La Plata, Argentina; 101 Physics Department, Lancaster University, Lancaster, UK; 102 INFN Sezione di Lecce, Lecce, Italy; 103 Dipartimento di Matematica e Fisica, Università del Salento, Lecce, Italy; 104 Oliver Lodge Laboratory, University of Liverpool, Liverpool, UK; 105 Department of Physics, Jožef Stefan Institute and University of Ljubljana, Ljubljana, Slovenia; 106 School of Physics and Astronomy, Queen Mary University of London, London, UK; 107 Department of Physics, Royal Holloway University of London, Surrey, UK; 108 Department of Physics and Astronomy, University College London, London, UK; 109 Louisiana Tech University, Ruston, LA USA; 110 Laboratoire de Physique Nucléaire et de Hautes Energies, UPMC and Université Paris-Diderot and CNRS/IN2P3, Paris, France; 111 Fysiska Institutionen, Lunds Universitet, Lund, Sweden; 112 Departamento de Fisica Teorica C-15, Universidad Autonoma de Madrid, Madrid, Spain; 113 Institut für Physik, Universität Mainz, Mainz, Germany; 114 School of Physics and Astronomy, University of Manchester, Manchester, UK; 115 CPPM, Aix-Marseille Université and CNRS/IN2P3, Marseille, France; 116 Department of Physics, University of Massachusetts, Amherst, MA USA; 117 Department of Physics, McGill University, Montreal, QC Canada; 118 School of Physics, University of Melbourne, Melbourne, VIC Australia; 119 Department of Physics, The University of Michigan, Ann Arbor, MI USA; 120 Department of Physics and Astronomy, Michigan State University, East Lansing, MI USA; 121 INFN Sezione di Milano, Milan, Italy; 122 Dipartimento di Fisica, Università di Milano, Milan, Italy; 123 B.I. Stepanov Institute of Physics, National Academy of Sciences of Belarus, Minsk, Republic of Belarus; 124 National Scientific and Educational Centre for Particle and High Energy Physics, Minsk, Republic of Belarus; 125 Group of Particle Physics, University of Montreal, Montreal, QC Canada; 126 P.N. Lebedev Physical Institute of the Russian, Academy of Sciences, Moscow, Russia; 127 Institute for Theoretical and Experimental Physics (ITEP), Moscow, Russia; 128 National Research Nuclear University MEPhI, Moscow, Russia; 129 D.V. Skobeltsyn Institute of Nuclear Physics, M.V. Lomonosov Moscow State University, Moscow, Russia; 130 Fakultät für Physik, Ludwig-Maximilians-Universität München, Munich, Germany; 131 Max-Planck-Institut für Physik (Werner-Heisenberg-Institut), Munich, Germany; 132 Nagasaki Institute of Applied Science, Nagasaki, Japan; 133 Graduate School of Science and Kobayashi-Maskawa Institute, Nagoya University, Nagoya, Japan; 134 INFN Sezione di Napoli, Naples, Italy; 135 Dipartimento di Fisica, Università di Napoli, Naples, Italy; 136 Department of Physics and Astronomy, University of New Mexico, Albuquerque, NM USA; 137 Institute for Mathematics, Astrophysics and Particle Physics, Radboud University Nijmegen/Nikhef, Nijmegen, The Netherlands; 138 Nikhef National Institute for Subatomic Physics and University of Amsterdam, Amsterdam, The Netherlands; 139 Department of Physics, Northern Illinois University, DeKalb, IL USA; 140 Budker Institute of Nuclear Physics, SB RAS, Novosibirsk, Russia; 141 Department of Physics, New York University, New York, NY USA; 142 Ohio State University, Columbus, OH USA; 143 Faculty of Science, Okayama University, Okayama, Japan; 144 Homer L. Dodge Department of Physics and Astronomy, University of Oklahoma, Norman, OK USA; 145 Department of Physics, Oklahoma State University, Stillwater, OK USA; 146 Palacký University, RCPTM, Olomouc, Czech Republic; 147 Center for High Energy Physics, University of Oregon, Eugene, OR USA; 148 LAL, University of Paris-Sud, CNRS/IN2P3, Université Paris-Saclay, Orsay, France; 149 Graduate School of Science, Osaka University, Osaka, Japan; 150 Department of Physics, University of Oslo, Oslo, Norway; 151 Department of Physics, Oxford University, Oxford, UK; 152 INFN Sezione di Pavia, Pavia, Italy; 153 Dipartimento di Fisica, Università di Pavia, Pavia, Italy; 154 Department of Physics, University of Pennsylvania, Philadelphia, PA USA; 155 National Research Centre “Kurchatov Institute” B.P.Konstantinov Petersburg Nuclear Physics Institute, St. Petersburg, Russia; 156 INFN Sezione di Pisa, Pisa, Italy; 157 Dipartimento di Fisica E. Fermi, Università di Pisa, Pisa, Italy; 158 Department of Physics and Astronomy, University of Pittsburgh, Pittsburgh, PA USA; 159 Laboratório de Instrumentação e Física Experimental de Partículas-LIP, Lisbon, Portugal; 160 Faculdade de Ciências, Universidade de Lisboa, Lisbon, Portugal; 161 Department of Physics, University of Coimbra, Coimbra, Portugal; 162 Centro de Física Nuclear da Universidade de Lisboa, Lisbon, Portugal; 163 Departamento de Fisica, Universidade do Minho, Braga, Portugal; 164 Departamento de Fisica Teorica y del Cosmos and CAFPE, Universidad de Granada, Granada, Spain; 165 Dep Fisica and CEFITEC of Faculdade de Ciencias e Tecnologia, Universidade Nova de Lisboa, Caparica, Portugal; 166 Institute of Physics, Academy of Sciences of the Czech Republic, Prague, Czech Republic; 167 Czech Technical University in Prague, Prague, Czech Republic; 168 Faculty of Mathematics and Physics, Charles University in Prague, Prague, Czech Republic; 169 State Research Center Institute for High Energy Physics (Protvino), NRC KI, Protvino, Russia; 170 Particle Physics Department, Rutherford Appleton Laboratory, Didcot, UK; 171 INFN Sezione di Roma, Rome, Italy; 172 Dipartimento di Fisica, Sapienza Università di Roma, Rome, Italy; 173 INFN Sezione di Roma Tor Vergata, Rome, Italy; 174 Dipartimento di Fisica, Università di Roma Tor Vergata, Rome, Italy; 175 INFN Sezione di Roma Tre, Rome, Italy; 176 Dipartimento di Matematica e Fisica, Università Roma Tre, Rome, Italy; 177 Faculté des Sciences Ain Chock, Réseau Universitaire de Physique des Hautes Energies-Université Hassan II, Casablanca, Morocco; 178 Centre National de l’Energie des Sciences Techniques Nucleaires, Rabat, Morocco; 179 Faculté des Sciences Semlalia, Université Cadi Ayyad, LPHEA-Marrakech, Marrakech, Morocco; 180 Faculté des Sciences, Université Mohamed Premier and LPTPM, Oujda, Morocco; 181 Faculté des Sciences, Université Mohammed V, Rabat, Morocco; 182 DSM/IRFU (Institut de Recherches sur les Lois Fondamentales de l’Univers), CEA Saclay (Commissariat à l’Energie Atomique et aux Energies Alternatives), Gif-sur-Yvette, France; 183 Santa Cruz Institute for Particle Physics, University of California Santa Cruz, Santa Cruz, CA USA; 184 Department of Physics, University of Washington, Seattle, WA USA; 185 Department of Physics and Astronomy, University of Sheffield, Sheffield, UK; 186 Department of Physics, Shinshu University, Nagano, Japan; 187 Fachbereich Physik, Universität Siegen, Siegen, Germany; 188 Department of Physics, Simon Fraser University, Burnaby, BC Canada; 189 SLAC National Accelerator Laboratory, Stanford, CA USA; 190 Faculty of Mathematics, Physics and Informatics, Comenius University, Bratislava, Slovak Republic; 191 Department of Subnuclear Physics, Institute of Experimental Physics of the Slovak Academy of Sciences, Kosice, Slovak Republic; 192 Department of Physics, University of Cape Town, Cape Town, South Africa; 193 Department of Physics, University of Johannesburg, Johannesburg, South Africa; 194 School of Physics, University of the Witwatersrand, Johannesburg, South Africa; 195 Department of Physics, Stockholm University, Stockholm, Sweden; 196 The Oskar Klein Centre, Stockholm, Sweden; 197 Physics Department, Royal Institute of Technology, Stockholm, Sweden; 198 Departments of Physics and Astronomy and Chemistry, Stony Brook University, Stony Brook, NY USA; 199 Department of Physics and Astronomy, University of Sussex, Brighton, UK; 200 School of Physics, University of Sydney, Sydney, Australia; 201 Institute of Physics, Academia Sinica, Taipei, Taiwan; 202 Department of Physics, Technion: Israel Institute of Technology, Haifa, Israel; 203 Raymond and Beverly Sackler School of Physics and Astronomy, Tel Aviv University, Tel Aviv, Israel; 204 Department of Physics, Aristotle University of Thessaloniki, Thessaloníki, Greece; 205 International Center for Elementary Particle Physics and Department of Physics, The University of Tokyo, Tokyo, Japan; 206 Graduate School of Science and Technology, Tokyo Metropolitan University, Tokyo, Japan; 207 Department of Physics, Tokyo Institute of Technology, Tokyo, Japan; 208 Department of Physics, University of Toronto, Toronto, ON Canada; 209 TRIUMF, Vancouver, BC Canada; 210 Department of Physics and Astronomy, York University, Toronto, ON Canada; 211 Faculty of Pure and Applied Sciences, and Center for Integrated Research in Fundamental Science and Engineering, University of Tsukuba, Tsukuba, Japan; 212 Department of Physics and Astronomy, Tufts University, Medford, MA USA; 213 Department of Physics and Astronomy, University of California Irvine, Irvine, CA USA; 214 INFN Gruppo Collegato di Udine, Sezione di Trieste, Udine, Italy; 215 ICTP, Trieste, Italy; 216 Dipartimento di Chimica Fisica e Ambiente, Università di Udine, Udine, Italy; 217 Department of Physics and Astronomy, University of Uppsala, Uppsala, Sweden; 218 Department of Physics, University of Illinois, Urbana, IL USA; 219 Instituto de Fisica Corpuscular (IFIC) and Departamento de Fisica Atomica, Molecular y Nuclear and Departamento de Ingeniería Electrónica and Instituto de Microelectrónica de Barcelona (IMB-CNM), University of Valencia and CSIC, Valencia, Spain; 220 Department of Physics, University of British Columbia, Vancouver, BC Canada; 221 Department of Physics and Astronomy, University of Victoria, Victoria, BC Canada; 222 Department of Physics, University of Warwick, Coventry, UK; 223 Waseda University, Tokyo, Japan; 224 Department of Particle Physics, The Weizmann Institute of Science, Rehovot, Israel; 225 Department of Physics, University of Wisconsin, Madison, WI USA; 226 Fakultät für Physik und Astronomie, Julius-Maximilians-Universität, Würzburg, Germany; 227 Fakultät für Mathematik und Naturwissenschaften, Fachgruppe Physik, Bergische Universität Wuppertal, Wuppertal, Germany; 228 Department of Physics, Yale University, New Haven, CT USA; 229 Yerevan Physics Institute, Yerevan, Armenia; 230 Centre de Calcul de l’Institut National de Physique Nucléaire et de Physique des Particules (IN2P3), Villeurbanne, France; 231 CERN, 1211 Geneva 23, Switzerland

## Abstract

The performance of the jet trigger for the ATLAS detector at the LHC during the 2011 data taking period is described. During 2011 the LHC provided proton–proton collisions with a centre-of-mass energy of 7 TeV and heavy ion collisions with a 2.76 TeV per nucleon–nucleon collision energy. The ATLAS trigger is a three level system designed to reduce the rate of events from the 40 MHz nominal maximum bunch crossing rate to the approximate 400 Hz which can be written to offline storage. The ATLAS jet trigger is the primary means for the online selection of events containing jets. Events are accepted by the trigger if they contain one or more jets above some transverse energy threshold. During 2011 data taking the jet trigger was fully efficient for jets with transverse energy above 25 GeV for triggers seeded randomly at Level 1. For triggers which require a jet to be identified at each of the three trigger levels, full efficiency is reached for offline jets with transverse energy above 60 GeV. Jets reconstructed in the final trigger level and corresponding to offline jets with transverse energy greater than 60 GeV, are reconstructed with a resolution in transverse energy with respect to offline jets, of better than 4 % in the central region and better than 2.5 % in the forward direction.

## Introduction

ATLAS [1] is one of two general purpose detectors at the Large Hadron Collider (LHC) [2]. During the 2011 running period the LHC operated with a collision energy of $\sqrt{s} = 7$ TeV, allowing ATLAS to collect an integrated luminosity of 5.25 fb$^{-1}$ during proton–proton (*pp*) collisions, and 158 $\mu$b$^{-1}$ during lead–lead (Pb+Pb) collisions with centre-of-mass energy of 2.76 TeV for each pair of colliding nucleons in the interaction.

The large event rate at the LHC makes the online selection of interesting physics events essential for achieving the physics goals of the LHC programme. During the 2011 data taking period, the LHC ran with a bunch spacing of 50 ns providing a nominal rate of 20 MHz, and with a mean of more than 20 separate *pp* interactions per bunch crossing (known as pile-up) towards the end of data taking. To reduce the rate of events to be read out from the detector to a rate of around 400 Hz which can be written to offline storage, a rejection factor greater than $10^5$ is required. This is achieved by the ATLAS trigger [3] which is divided into the Level 1 (L1) trigger and the High Level Trigger (HLT). In 2011 the HLT itself consisted of two levels: Level 2 (L2) followed by the event filter (EF).

The jet trigger system of the ATLAS detector is the primary means to select events containing jets with high transverse energy ($E_\mathrm{T}$). It selects collision events to be used in jet physics analyses [4–11], as well as in many other analyses where one or more jets may be required, perhaps in conjunction with additional physics signatures such as an isolated lepton candidate. In this paper, the design and performance of the ATLAS jet trigger during the 2011 data taking is described.

The outline of this paper is as follows: Sect. 2 describes the design of the ATLAS jet trigger. Section 3 provides an overview of the jet based event selection defined in the ATLAS trigger menu and explains the nomenclature used for trigger names. The timing, or CPU budget, of each trigger level is outlined in Sect. 4. Various aspects of jet trigger performance are described in Sect. 5, which outlines the measures used for the evaluation of the trigger performance, and includes the selection efficiencies for inclusive single jet and multi-jet triggers. Descriptions of specialised triggers designed for specific physics selections are provided in Sect. 6. These include selections for triggering on large summed scalar $E_\mathrm{T}$ or boosted objects that can decay into multiple narrow jets. Event selection in the Pb+Pb programme is described in Sect. 7.

### The ATLAS detector and trigger system

**Fig. 1:**
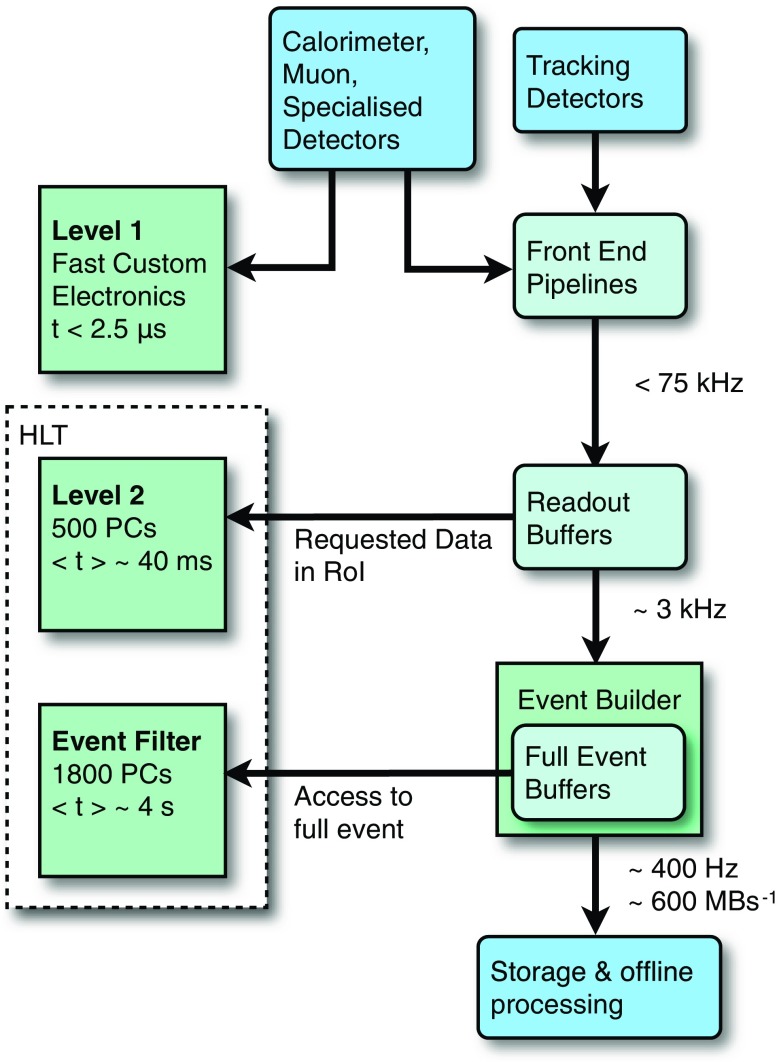
The ATLAS trigger system

The ATLAS detector is a multi-purpose particle detector with a forward-backward symmetric cylindrical geometry and a near $4\pi$ coverage in solid angle^1^. Owing to the cylindrical geometry, subdetector components are described as being in the *central region*, if they are part of the barrel, at small absolute pseudorapidity, or described as *forward*, if part of the endcaps at large absolute pseudorapidity. Outwards from the beam pipe, ATLAS consists of an inner tracking detector surrounded by a thin superconducting solenoid providing a 2 T axial magnetic field, electromagnetic and hadronic calorimeters, and a muon spectrometer. The inner tracking detector covers the pseudorapidity range $|\eta | < 2.5$ and consists of silicon pixel, silicon microstrip, and transition radiation tracking detectors.

The calorimeters cover the region $|\eta | < 4.9$ and consist of electromagnetic (EM), and hadronic subsystems. The EM, the hadronic endcap (HEC), and the forward calorimeters (FCal) use liquid argon (LAr) as the active medium, and either a lead, copper or tungsten absorber technology. The EM calorimeter is divided into a barrel part, $|\eta |<1.475$, and two endcap components with $1.375<|\eta |<3.2$. The central hadronic calorimeter, referred to as the tile calorimeter, uses steel absorber layers interleaved with plastic scintillator covering the pseudorapidity range $|\eta | < 1.7$. A presampler is installed in front of the EM calorimeter for $|\eta | < 1.8$. For the calorimeter subsystems, there are two separate readout paths: the first, a very fast readout of combined towers of calorimeter cells, is used at Level 1, while the second is the slower readout of the full calorimeter cell information for use in the HLT and offline.

The muon spectrometer surrounds the calorimeters and is based on three large air-core toroid superconducting magnets with eight coils each. The toroid bending power ranges from 2.0 to 7.5 Tm. The muon spectrometer includes a system of precision tracking chambers and fast detectors for triggering.

The ATLAS trigger [3, 12, 13] for 2011 consisted of three processing levels, each allowing increasingly detailed reconstruction and selection algorithms. This approach enables the successive identification of potentially interesting features and the early rejection of less interesting events. The L1 trigger runs hardware algorithms over data with reduced spatial granularity from the calorimeter and muon subsystems to identify geometrical *regions of interest* (RoI) in the detector, containing candidate physical objects which should be examined more closely in subsequent trigger levels. The L1 trigger has a fixed maximum latency of 2.5 $\mu$s, and a rate for accepting events up to 75 kHz. For standard triggers, events with at least one RoI passing the L1 selection are passed to the L2 system, which runs software algorithms on a farm of commodity CPUs. The L2 algorithms have access to the data at the full detector granularity but only from those detector elements that lie within an RoI. The number of processors in the L2 farm and the time taken to process each event provides a limit on the rate at which events can be accepted by the L1 system.

Following the L2 processing, all events with RoIs that satisfy a set of predefined selection criteria are passed to the event builder which reads out the detector at full granularity. These fully built events are then processed by the EF, which also consists of a farm of commodity CPUs. The EF farm runs modified versions of the offline reconstruction algorithms, simplified to improve the speed of execution. Although the full event data are available at the EF, for many trigger signatures the EF trigger reconstruction takes place within RoIs for reasons of speed. This is not the case for the jet trigger, for which the whole detector is read out. The rate of L2 accepted events passed to the EF during 2011 was approximately 3 kHz, and the rate at which events were read out for offline storage was approximately 400 Hz. The ATLAS trigger is illustrated in Fig. 1.

In 2011 the full jet trigger was operated in rejection mode for the first time, allowing events to be discarded at each of the three trigger levels. Prior to 2011, the ATLAS trigger selection for events containing jets was based purely on the algorithms running at L1 and L2, with the EF algorithms executed in commissioning mode only. In this mode, events were processed by the EF but not rejected should they have failed the EF requirements. The resulting trigger decision was stored in the event stream for commissioning purposes.

## Jet trigger design overview

The jet trigger is an integral part of the ATLAS trigger system, processing events based on successively more detailed detector information at the L1, L2 and EF stages. Hadronic and electromagnetic energy deposits in the calorimeter subsystems are used to reconstruct jets; fast, custom jet algorithms are used at L1 and L2; and for the EF, the anti-$k_{t}$ [14] algorithm in the four-momentum recombination scheme, implemented in the FastJet [15] package is used. In each of the three stages, the bandwidth allocated to the jet trigger is about 10 % of the total. Jet trigger signatures, simply referred to as jet triggers, are divided into either *central* or *forward*, with the central jet triggers using detector data from the central and endcap calorimeters ($|\eta |< 3.2$) and the forward jet triggers in the region $3.2<|\eta |< 4.9$ using data from the FCal. Different electronics are used for each to take account [16] of the more coarse FCal detector granularity in the forward direction.

The L1 calorimeter trigger system (L1Calo) [17], is the first stage of the jet trigger. This reconstructs jets from the combined energy deposits in the LAr and tile calorimeters, using collections of calorimeter cells projecting back to the nominal interaction point, known as *trigger towers*. A square sliding window of $0.8\times 0.8$ in $\Delta \eta \times \Delta \phi$ is used to identify regions where the summed transverse energy within the central $0.4\times 0.4$ region of the window is large and corresponds to a local maximum [18, 19].

The jet candidate $E_\mathrm{T}$ values are then compared to a set of predefined $E_\mathrm{T}$ thresholds to decide which candidates should form an RoI. The trigger thresholds are discussed in Section 3. Information about the regions of the detector that contain jet candidates – specifically the multiplicity of candidates exceeding each threshold – is sent to the central trigger processor (CTP) and used in the generation of the global L1 decision. This is then distributed to the detector front-end electronics, to initiate the data readout, and the subsequent stages of the trigger. Information on which jet thresholds from L1 have been satisfied can also be combined with information from other L1 trigger subsystems, such as electron or muon triggers, to produce multi-object triggers.

The data from events which pass the L1 selection are processed by the L2 trigger, which has access to the calorimeter cells within the RoIs identified by L1. Limiting the data processed in this way allows the detailed trigger reconstruction of any potentially interesting object, whilst requiring typically only 1–2 % of the full detector data corresponding to the detector elements within the RoIs to be read out. The L2 jet trigger runs a feature extraction algorithm consisting of a simple, iterative cone algorithm (described in Sect. 2.2.2) to build jets using the full detector granularity. The L1 RoI corresponding to the jet is said to *seed* the L2 processing in the HLT. The characteristics of jets found using the iterative cone algorithm are tested with a *hypothesis algorithm* to determine if they fulfil the predetermined L2 trigger selection criteria. These criteria may include minimum values for the jet transverse energy, and selection on the jet pseudorapidity and quality. Each event selected at L2 is then fully built from the various fragments temporarily stored in memory in the data acquisition system.

The final stage of the trigger, the EF, must perform jet reconstruction in the full event within approximately 4 s before making a decision on whether to write the event to offline storage. Due to the larger available latency at the EF compared to L2, more sophisticated reconstruction algorithms can be applied. To the maximum extent possible, the EF uses standard ATLAS event reconstruction algorithms developed for offline analysis, as well as final offline detector calibrations. Since the EF runs after the full event has been built by the event builder, it is able to access information from the complete detector, rather than just that from detector elements in an RoI. The EF jet trigger reconstructs anti-$k_{t}$ jets in the full calorimeter, in the same manner as the standard offline jet reconstruction, rather than separately processing data within individual RoIs.

The ability of the EF to operate on the full calorimeter data permits seeding by triggers which select, at random, some fraction of events from L1 at a predefined rate irrespective of whether any RoI is present. Using the random trigger in this way allows the EF to trigger on jets free from any bias that might be introduced by the jet reconstruction at either the L1 or L2 stages. This is particularly useful for lower $E_\mathrm{T}$ jet thresholds, where such biases can be large.

### Level 1

The L1 trigger decision is based on analogue sums of signals from calorimeter elements within 7168 projective regions (trigger towers), independent of the precision readout used in the HLT and offline. Trigger towers have a size of approximately $\Delta \eta \times \Delta \phi = 0.1 \times 0.1$ in the central part of the calorimeter within $|\eta | < 2.5$, and are larger and less regular in the more forward regions. Electromagnetic and hadronic calorimeters have separate trigger towers. The 7168 analogue inputs to the L1 calorimeter trigger are first digitised and then assigned to a particular LHC bunch crossing.

Two separate processor systems, working in parallel, run the trigger algorithms. One system, the cluster processor, uses the full L1 trigger granularity information in the central region to look for small localised calorimeter energy clusters typical of electrons, photons or the products of tau lepton decays. The other, used for jet and missing energy triggers, uses coarser granularity *jet elements*, to identify jet candidates and form global $E_\mathrm{T}$ sums: missing $E_\mathrm{T}$, total $E_\mathrm{T}$, and the scalar sum of all jet $E_\mathrm{T}$. The jet elements consist of $2\times 2$ arrays of trigger towers in the central region and fewer in the foward region where the trigger towers are larger. The $E_\mathrm{T}$ of individual energy depositions and the $E_\mathrm{T}$ sums are compared to preprogrammed trigger thresholds to form the trigger decision. Jet RoIs are defined as $4\times 4$ jet element windows for which the summed electromagnetic and hadronic calorimeter $E_\mathrm{T}$ exceeds predefined thresholds and which encompass a $2\times 2$ jet element core where the hadronic calorimeter $E_\mathrm{T}$ is a local maximum. The location of the centre of this $2\times 2$ array defines the coordinates of the jet RoI.

### Level 2

In order to handle the large event rate from the detector, following a decision to accept an event at L1, the L2 decision must arrive within approximately 40 ms. Even with the reduction in data volume from reading out only those data corresponding to the RoIs identified by L1, the data preparation at L2 still represents a large contribution to the overall processing time. In this section the data preparation and jet finding stages of the L2 system are discussed.

#### Level 2 data preparation

The data preparation for the L2 jet trigger is a crucial part of the L2 processing. It provides the collection of data from detector readout drivers (RODs) [12], delivery to the L2 processing units, and the conversion from the raw data into the objects used by the HLT algorithms. The RODs receive data from the calorimeter front-end boards via optical fibres. These boards are installed on the detector and contain electronics for the amplification, shaping, sampling, pipelining, and digitisation of the calorimeter signals [20, 21]. Due to the large number of calorimeter readout channels, approximately $2\times 10^5$, and in order to meet the L2 timing performance goal of 40 ms per event, the data volume read out should be kept to the minimum required to avoid compromising algorithm performance. For each detector element (calorimeter cell) within the RoI window, the direction from the nominal interaction point to the element position is binned in a grid in the $\eta$–$\phi$ plane, for use in the L2 jet reconstruction algorithm.

#### Level 2 jet reconstruction algorithm

At L2, jets are defined as cone-shaped objects [16] in the $\eta$–$\phi$ plane with a given radius, *R*, such that they contain energy deposits with a separation $\Delta R\equiv \sqrt{(\Delta \eta )^2+(\Delta \phi )^2} < R$, where $\Delta \eta$ and $\Delta \phi$ are defined with respect to the jet axis. The value of the radius parameter, *R*, is set during the trigger configuration. The jet energy and position are found through an iterative procedure using the grid in $(\eta , \phi )$ populated by the cell energies, with the following steps:

- First, an initial reference jet, $j_0$, is defined by the L1 jet RoI position with the predefined cone radius *R*. Note that the possible directions of the reference jet are discrete due to the 0.2 $\times$ 0.2 granularity at L1.
- The *k* elements from the binned distribution that fall within the ($\eta$, $\phi$) region encompassed by the reference jet, $j_0$, are used to recalculate the jet energy and the energy weighted average position of the jet, to define a new, updated reference jet $j_1$, according to
  1 $$\begin{aligned} E_{j_1}= & {} \sum _{i=1}^{k} E_{i},\end{aligned}$$
  2 $$\begin{aligned} \eta _{j_1}= & {} \frac{\sum _{i=1}^{k}E_{i}\eta _{{i}}}{\sum _{{i=1}}^{k}E_{i}},\end{aligned}$$
  3 $$\begin{aligned} \phi _{j_1}= & {} \phi _{{j_0}}+\frac{\sum _{{i=1}}^{{k}}E_{{i}}\times (\phi _{{i}}-\phi _{ {j_0}})}{\sum _{{i=1}}^{{k}}E_{{i}}}. \end{aligned}$$
  where the sum runs over the *k* grid elements whose centroids are contained within the cone of radius *R* centred on the reference jet $j_0$. The total energy, and coordinates $\eta _{j_1}$ and $\phi _{j_1}$, are computed from Eqs. (1),  (2) and (3).
- The previous step is repeated with $j_0$ replaced by $j_1$ in Eqs. (2) and (3), and so on to form jet $j_i$ from jet $j_{i-1}$, updating the jet energy $E_{j_i}$ and the coordinates $\eta _{{j_i}}$ and $\phi _{{j_i}}$. The iteration is repeated *N* times to create jet $j_{{N}}$. A configurable number of iterations are executed. Typically, $N=3$ is used, having been found sufficient to achieve the required performance [16].

The result of this algorithm is a jet defined by the reconstructed ($\eta$, $\phi$) direction, and the total jet energy. This energy is evaluated at the electromagnetic calorimeter energy scale, by summing the energy depositions in the electromagnetic and hadronic parts of the calorimeter without applying any further calibration.

For the central jet trigger, $R=0.4$ is used. For the forward jet trigger, because of the coarse granularity of the FCal, the radii used for the first and second iterations are 1.0 and 0.7 respectively, to ensure that the energy deposits are fully contained given the coarse position available for the L1 jet. For the final iteration, the radius $R=0.4$ is used.

#### Level 2 full scan trigger

Towards the end of data taking in 2011 a new *Level 2 full scan* trigger [22, 23], using the lower granularity trigger tower data from Level 1, was introduced. Here, the trigger tower data for the full calorimeter for each event was read out by the Level 2 system and processed on the Level 2 CPU farm with the anti-$k_{t}$ algorithm. This trigger was running in commissioning mode only during the heavy ion run at the end of 2011 and was not deployed for production data taking in the proton–proton jet trigger until 2012.

### Event filter

The EF is the last stage in the trigger and is responsible for the final decision of whether an event should be sent to offline storage or discarded. The jet trigger at the EF is modular and makes use of three general stages; data preparation (calorimeter unpacking and pre-clustering), jet finding, and hypothesis testing.

**Fig. 2:**
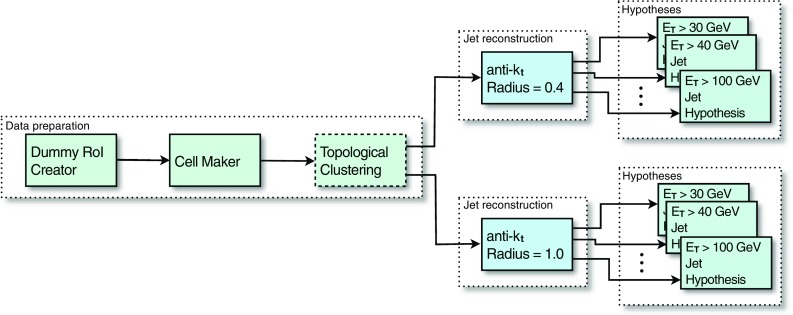
The stages of algorithm processing in the Event Filter for several inclusive single jet triggers with different $E_\mathrm{T}$ thresholds. The case illustrated shows two sets of signatures, each set with a different jet radius parameter

In contrast to the RoI-based approach used at L2, the EF runs the jet finding algorithm once per event for each configured jet radius, using data from the complete calorimeter. This is referred to as a *full scan*. The full scan approach has several advantages for jet reconstruction with respect to the RoI based approach used at L2. The large RoIs required at L2 to ensure that any jet is completely contained has the unfortunate disadvantage that RoIs may overlap in events with high jet multiplicity, resulting in some parts of the detector being processed multiple times. This can result in jets being fully, or partially reconstructed in several RoIs, which may cause the double counting of energy deposits and jets, which would affect the multi-jet signatures. The full scan approach completely eliminates the multiple processing of regions of the detector and, as a consequence, leads to faster processing in high occupancy events, although it takes longer in low occupancy events, where the processing time is in any case low.

Since the output from L2 is in the form of lists of RoIs passing each trigger threshold, a slightly different approach is required to seed the EF processing. In this case, the first jet RoI to be processed by the EF initiates the creation of a dummy, full scan RoI, encompassing the entire detector, required to ensure that the entire calorimeter is processed. The calorimeter cell data for this full detector RoI is then extracted by the *cell maker* and processed to provide the objects upon which the jet finding will then run. Following the jet finding, hypothesis algorithms are executed. These determine whether any specific jet selection signatures are satisfied, for example, typical selections are those based on specific jet $E_\mathrm{T}$ thresholds.

The objects from both the data preparation and the jet finding stages are cached for this full scan RoI. When evaluating any additional trigger signature requiring jets passing a different $E_\mathrm{T}$ threshold in the same event, the trigger can establish that this dummy RoI has already been created and will not start the sequence for the data preparation and jet finding again, instead simply retrieving the jets from the cache. The hypothesis algorithm for this different $E_\mathrm{T}$ threshold will then be executed.

Since the cell data are cached following the data preparation stage, the jet algorithms with different radius parameters run over the cached data and only the jet finding itself will be executed again for each different required radius. In this way the data preparation is common to all jet finding, which is in turn executed only once for each jet radius required. The full sequence for multiple thresholds and multiple jet radius parameters is illustrated in Fig. 2 and the individual stages are discussed in more detail below.

#### Event filter data preparation

The jet finder stage can operate with a number of different types of input objects produced by the data preparation from the raw cell data. In early 2011 the primary objects used as input to jet finding were projective calorimeter towers constructed from the raw calorimeter cell information. From May 2011, so-called *topological clusters* [18] were used. These are discussed later. Since the offline jet reconstruction also uses topological clusters, this improves the EF jet resolutions with respect to offline reconstruction, although the topological clustering algorithm does add additional processing time to the data preparation stage.

The topological clustering algorithm creates clusters of topologically related energy deposits. The algorithm starts with a seed calorimeter cell, with an energy deposit with absolute value greater than four standard deviations above the expected noise. All cells directly neighbouring these seed cells, in all three dimensions, are collected into the cluster. Cells adjacent to the cluster are then added, if they have an energy with an absolute value exceeding the noise by two standard deviations, iterating until all such adjacent cells have been used. Finally, a ring of guard cells is added to complete the cluster. After the initial clusters have been formed, they are analysed to identify local maxima, and split should more than one such maxima be found in a cluster [18].

#### Pile-up noise suppression

Jet reconstruction in the trigger is affected by the presence of pile-up interactions, which give rise to energy deposits in the calorimeter that are unrelated to the primary interaction of interest. The overlap of these energy deposits with those of the jets of interest can distort the reconstructed direction and $E_\mathrm{T}$ of the jet. Due to the long integration time of the calorimeter electronics – up to 600 ns [1] – the detector response is also dependent on energy deposits arriving earlier or later than the nominal beam crossing. The size and likelihood of contributions due to pile-up depend on the number of interactions per bunch crossing. To account for this, the noise thresholds applied during the topological clustering process were tuned at the start of the 2011 running period to reflect the expected mean number of interactions per bunch crossing.

#### Jet finding and hypothesis testing

Jet finding can be performed using any of the available offline jet algorithms. Due to problems with the infrared and collinear safety of cone algorithms [24], ATLAS has adopted $k_\perp$-ordered sequential combination algorithms [25, 26], and specifically the anti-$k_{t}$ [14] algorithm in the four-momentum recombination scheme as the jet algorithm of choice for physics analyses [4–6, 8]. To match this offline choice, the anti-$k_{t}$ algorithm was chosen for use in the EF for 2011 data taking, to replace the ATLAS cone [27, 28] jet algorithm used in the trigger prior to 2011. Two different values of the radius parameter, *R*, were used in the EF trigger reconstruction in 2011, $R=0.4$ and 1.0, the larger radius being useful for the study of hadronic decays of boosted heavy particles.

Should any additional calibrations be required for the final jets themselves, the jet reconstruction process can run a post-processing stage to apply them to jets. As in the case of the offline processing, the EF jet algorithm runs on the full calorimeter information. Differences between the trigger and offline jets generally only arise because the final offline calibrations are not normally available at the time of data taking. During the 2011 data taking the jet trigger was operated at the electromagnetic scale, i.e. with no jet calibration applied.

For an inclusive single jet trigger, the hypothesis algorithm that executes following the jet finding, accepts events which have at least one jet which satisfies the required criteria. Since the jets for each event are cached in memory, subsequent calls to hypothesis algorithms with different selection thresholds simply use this cached jet collection. Identifying multi-jet events is also simply a case of iterating over the reconstructed jets to identify combinations which pass the relevant selections for each signature. Different multi-jet signatures are possible, including those where the $E_\mathrm{T}$ of each jet in the event is required to exceed a different $E_\mathrm{T}$ threshold. The hypothesis algorithm takes as parameters the required jet multiplicity, *n*, the $\eta$ range within which the jets must lie, $\eta _{\mathrm {min}} \le |\eta _{\mathrm {jet}}| < \eta _{\mathrm {max}}$, and the required $E_\mathrm{T}$ thresholds for each of the required *n* jets.

## The jet trigger menu

The trigger system is configured via a menu which includes the specification of the list of event signatures to be accepted for events written to offline storage. For the jet trigger, this includes the number of jets, $E_\mathrm{T}$ thresholds, $\eta$ ranges, and other parameters such as jet-quality criteria, to be applied at each of the three trigger levels. The aim of the menu design is to deploy a complementary and robust set of selections for physics channels of interest, compatible with the given bandwidth limitations. The trigger menu determines the configuration of the L1 firmware and the algorithms executed at the HLT. Corresponding triggers in each of the three trigger levels constitute a trigger *chain*.

The names of the trigger selections used in this document consist of the jet multiplicity followed by the $E_\mathrm{T}$ threshold separated with a *j* for L2 and the EF, or *J* for L1. This is preceded by the trigger level separated by an underscore, so for instance *EF_j100* would be a 100 GeV single-jet trigger at the EF, and *L1_5J10 * would be a five jet trigger at L1 with a 10 GeV transverse energy requirement on each jet. Additional items may be included in the name for specialised triggers, such as *FJ* for forward jets which are required to have $|\eta |>3.2$. Typically the item names also include information regarding the specific jet algorithm. For instance *a4tc* or *a10tc* indicate that the anti-$k_{t}$ algorithm was used, with radius parameters 0.4 or 1.0 respectively, and running on topological clusters (*tc*). Where this string is omitted, anti-$k_{t}$ jets with radius parameter $R=0.4$ should be assumed. All the jet triggers used at the EF during 2011 were full scan triggers, and as such had names appended by *EFFS* to indicate the EF full scan; however, for the following discussion, the *EFFS* may be omitted from the trigger name for brevity.

Trigger selections at each level are designed to reduce the CPU usage at later trigger levels by maximising event rejection at early stages. Trigger thresholds in the higher levels are tightened to avoid the distortion of the efficiency curve from the slower-rising efficiency of previous levels. Triggers can operate in *pass-through* mode, which entails executing the trigger algorithms but accepting the event irrespective of the algorithm decision. This allows the trigger selections and algorithms to be validated, to ensure that they are robust against the varying beam and detector conditions, which are hard to predict before data taking. Partial pass-through mode allows only a certain percentage of events to be passed through the trigger in this way, the rest being subject to the usual trigger selection. This operational mode was used during data taking for several triggers. Passing events through in this way allows data to be collected by the higher threshold triggers for performance evaluation and debugging, with as little bias as possible.

Further flexibility is provided by defining *bunch groups*, which allow triggers to include specific requirements on the LHC bunches colliding in ATLAS. Not all bunch crossings contain protons; those that do are called *filled* bunches. For the random trigger, filled bunch crossings were required, indicated in the trigger name by *FILLED* at L1, and *filled* at L2. Non-collision triggers require a coincidence with an *empty* or *unpaired* bunch crossing, which correspond respectively to no protons in either LHC beam or a filled bunch in only one beam. For some of the lowest threshold physics triggers, a corresponding non-collision trigger was included in the chain for background studies.

As well as the trigger chains selecting jets at both L1 and L2, there were chains running at the EF, which were seeded by a random trigger at L1, and passed the events through L2 without running a selection algorithm. These allowed triggering on very low $E_\mathrm{T}$ jets at the EF without the biases introduced by the L1 jet reconstruction at low $E_\mathrm{T}$.

In addition to the more common jet triggers such as inclusive single jet, and multi-jet triggers, some specialised jet triggers, dedicated to more specific physics signatures, were used in 2011:

- Event Filter triggers that reconstruct $H_\mathrm{T}$, the total scalar sum of $E_\mathrm{T}$ of all jets in an event. Such triggers are useful for physics analyses which study or search for events with a large summed $E_\mathrm{T}$ in the final state, as the requirement of large $H_\mathrm{T}$ can help to control the trigger rate without requiring e.g. a very energetic leading jet;
- jet triggers where the jet algorithm is executed with a large-$R$ parameter, useful for searching for heavy particles decaying into boosted hadronic final states; the anti-$k_{t}$ algorithm was used with $R=1.0$ (denoted *a10*);
- heavy ion triggers, used for the Pb+Pb data taking period at the end of 2011, having a total transverse energy requirement in GeV denoted by *TE*, differing with respect to the $H_\mathrm{T}$ requirement used in proton runs in that TE is the sum of all transverse energy in the calorimeter, not only of that clustered in jets.

**Fig. 3:**
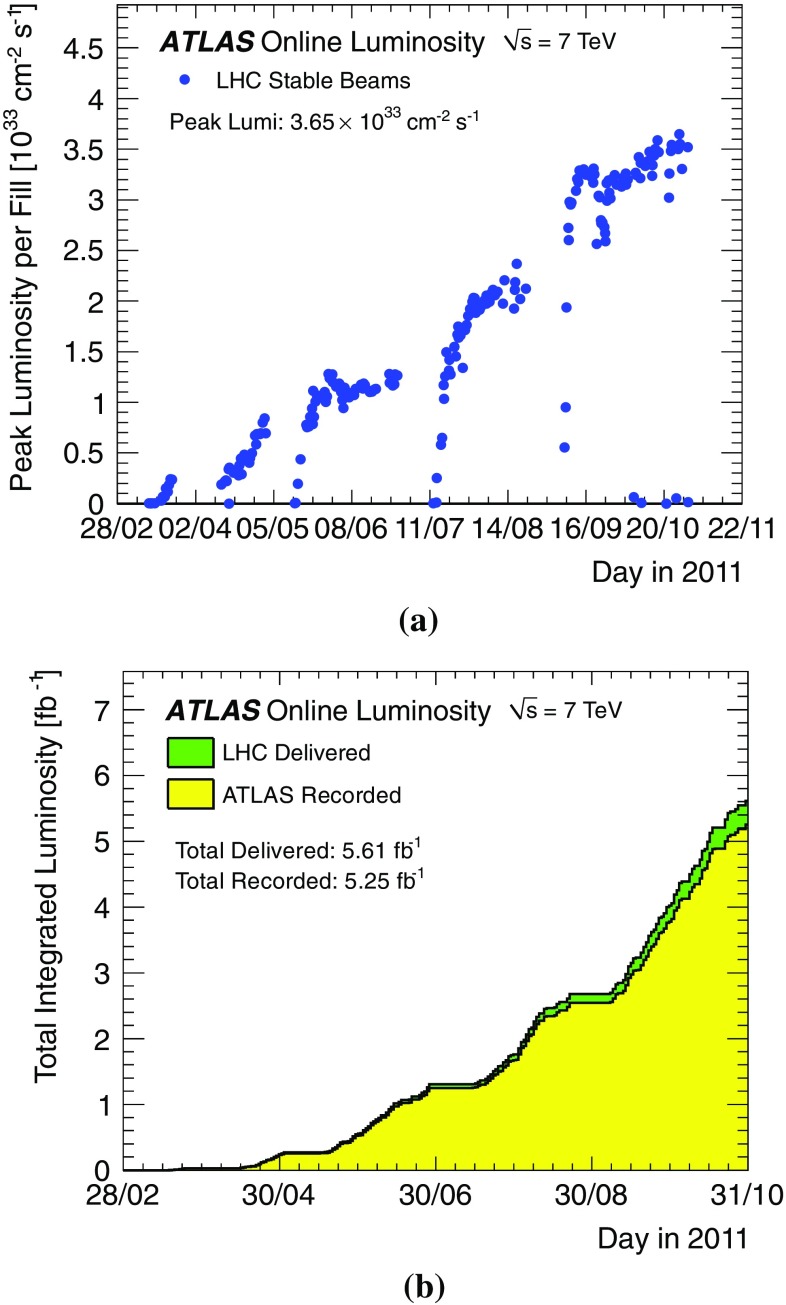
The luminosity measurement at the ATLAS interaction region for 2011 data taking [29]: **a** the maximum instantaneous luminosity versus day delivered to ATLAS during stable beam operation; **b** the cumulative luminosity versus day delivered to (*green*), and recorded by (*yellow*) ATLAS during stable beam operation for *pp* collisions at 7 TeV centre-of-mass energy

The first time ATLAS used both the L2 and EF stages of the HLT in event rejection mode was in 2011. A number of key improvements were introduced during that year, including the ability to use topological clusters rather than calorimeter towers at the EF, as discussed in Sect. 2.3.1, which was found to increase the stability of the algorithm in the presence of pile-up. During the 2011 data taking period the LHC peak instantaneous luminosity increased by more than an order of magnitude, from $10^{32}\,\text {cm}^{-2}\text {s}^{-1}$ to $3.6\times 10^{33}\,\text {cm}^{-2}\text {s}^{-1}$. Figure 3 shows the maximum instantaneous luminosity and the integrated luminosity delivered to ATLAS during 2011 as a function of time. The highest values for the mean number of interactions per bunch crossing reached $\sim$20 towards the end of running in 2011. The jet trigger menu evolved during this period to adapt to the changing LHC conditions.

**Fig. 4:**
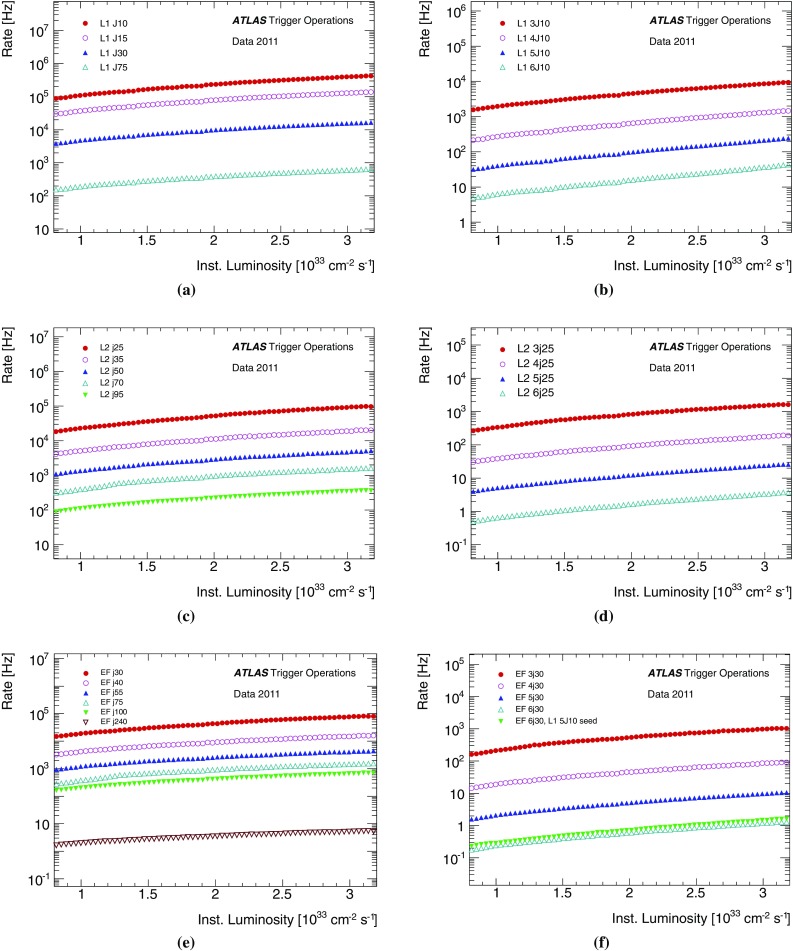
The jet trigger rates versus instantaneous luminosity, before application of prescale factors, for triggers operating in 2011 **a**, **c**, **e** for several single inclusive jet triggers; **b**, **d**, **f** for multi-jet triggers. Shown are the rates for L1, L2 and EF signatures

In order to keep the rate from the jet trigger within the allowed bandwidth, *prescale* factors are used to suppress the rates from signatures with lower thresholds. A prescaled trigger selects only a fraction, 1/*prescale*, of events that would otherwise pass the trigger. For the best expected statistical significance, wherever possible, triggers intended for searches or analyses requiring the highest possible number of data events, should not be prescaled. As the luminosity increased during 2011 data taking, the prescale factors applied to the triggers with lower thresholds were increased accordingly, to ensure that the output rate remained within the available bandwidth for writing to offline storage. Figure 4 illustrates the evolution of jet trigger rates with instantaneous luminosity for a selection of single inclusive jet and multi-jet triggers operating in 2011 at each of the three trigger levels. The rates shown are before application of any prescales. Typical prescale factors for the inclusive jet signatures applied on two separate dates during 2011 can be seen in Table 1.

**Table 1:** Typical values for the L1 and HLT prescales for the inclusive jet signatures, here denoted by the EF signatures, on two dates from different running periods. Also shown is the effective full chain prescale obtained by multiplying the L1 and HLT prescales. The three lowest $E_\mathrm{T}$ signatures are seeded by a random trigger at L1 with the same prescale, but have separate prescales at the HLT to control the rate. The remaining signatures are seeded by a jet trigger at both L1 and L2

	Apr 28$^\mathrm{th}$			Oct 22$^\mathrm{nd}$		
Trigger	L1 prescale	HLT prescale	Combined	L1 prescale	HLT prescale	Combined
EF_j10_a4tc$^\dagger$	2710	60.9	165039	58600	18.6	1089960
EF_j15_a4tc$^\dagger$	2710	12.4	33604	58600	4.3	251980
EF_j20_a4tc$^\dagger$	2710	3.8	10298	58600	1.2	70320
EF_j30_a4tc	7550	1	7550	39300	1	39300
EF_j40_a4tc	5080	1	5080	25300	1	25300
EF_j55_a4tc	1110	1.3	1443	3940	1.8	7092
EF_j75_a4tc	404	1	404	1910	1	1910
EF_j100_a4tc	1	116	116	1	529	529
EF_j135_a4tc	1	3	3	1	135	135
EF_j180_a4tc	1	1	1	1	31.6	31.6
EF_j240_a4tc	1	1	1	1	1	1
EF_j320_a4tc$^\ddagger$				1	1	1
EF_j425_a4tc$^\ddagger$				1	1	1

$^\dagger$ Randomly seeded at L1, passthrough at L2

$^\ddagger$ Not active during early running

**Table 2:** The evolution of the lowest $E_\mathrm{T}$, unprescaled EF threshold for single-jet triggers during 2011 data taking

Instantaneous luminosity	Lowest unprescaled trigger
$[10^{33} \text {cm}^{-2}\text {s}^{-1}]$	$E_\mathrm{T}$ threshold [GeV]
0 – 0.16	100
0.16 – 0.25	135
0.25 – 1.1	180
1.1 – 3.6	240

In addition to applying prescale factors to low-threshold triggers, the EF $E_\mathrm{T}$ threshold of the lowest-$E_\mathrm{T}$ unprescaled single inclusive jet trigger was raised on three occasions to accommodate the increasing instantaneous luminosity. The evolution of the minimum unprescaled EF threshold is detailed in Table 2 and effectively determines the lowest trigger threshold which can be used in several physics analyses. Technical improvements were implemented to improve trigger rejection and cope with the increasing luminosity and varying LHC conditions. From May 2011, calorimeter noise suppression and pile-up corrections were applied in the L2 calorimeter data preparation in order to reduce sudden increases in the trigger rate due to bad detector conditions, as well as maintaining performance under higher pile-up conditions.

## Timing

As a hardware system, the L1 trigger operates with a fixed latency, whereas the L2 and EF systems operate with a variable processing time, and must complete their respective processing within the constraints provided by the L1 rate, the rate at which events can be recorded offline, and the number of available CPU nodes in each HLT farm. In this section, the time taken to process events for the L1 system and the HLT is discussed.

### Level 1

The L1 jet trigger is a fixed latency, hardware based trigger operating synchronously with the LHC bunch clock and the rest of the L1 system. The pipelines in the detector front-end electronics are typically 120 bunch crossings deep and as such the latency from the complete L1 processing must fit within the corresponding time. Throughout the L1 system each step is handled in parallel with other steps. Data transfers between parts of the system are performed concurrently with the processing of the data that has already been transferred. The analogue data are digitised and sent as input to a jet algorithm, and the final decision is sent from the L1 calorimeter system to the central trigger processor (CTP). The jet algorithm processing itself is very fast and takes only approximately 50 ns, but represents only part of the processing necessary to reconstruct jet candidates, the rest being in formatting the input and output data such that the algorithm can execute quickly. The overall time for all these stages including the transmission of the results of the calorimeter trigger reconstruction to the CTP is approximately 1.5 $\mu$s. The additional time required for the subsequent CTP processing to determine the global L1 decision, and the time taken for transmission of this decision back to the detector front-end is approximately 0.5 $\mu$s so that the full latency of the entire L1 system is within the required maximum 2.5 $\mu$s.

### High level trigger

**Fig. 5:**
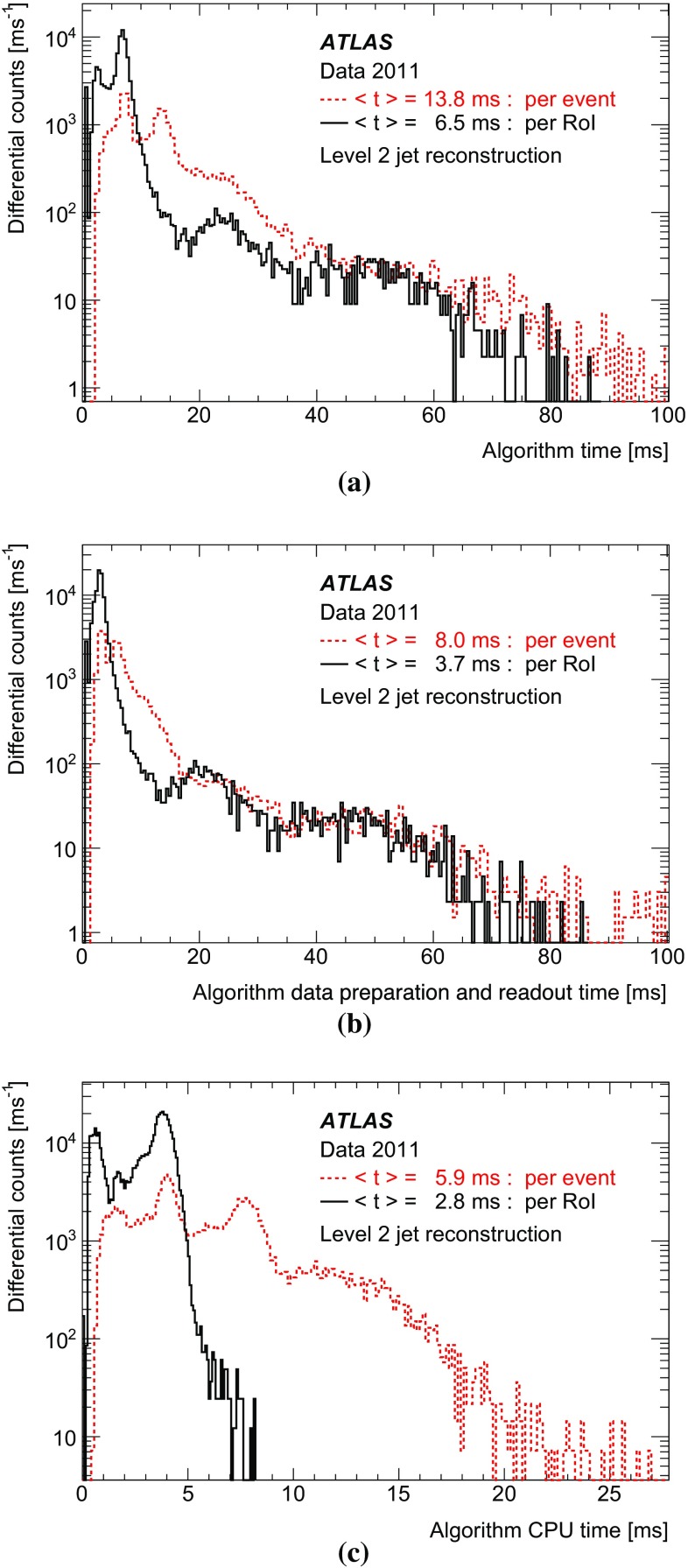
The processing time for the L2 jet trigger: **a** the full algorithm time; **b** the data preparation time; **c** the algorithm processing CPU time. The full algorithm time includes both the data preparation and algorithm processing times. The *solid lines* show the processing time per call, and the *dashed lines* show the processing times per event

In this section, timing distributions are presented for a physics run taken during October 2011. During this run, the peak instantaneous luminosity was 3.5$\times 10^{33}$ cm$^{-2}$ s$^{-1}$ with a mean of 17 interactions per bunch crossing at the start of the fill. The total L2 processing time is shown in Fig. 5a. This includes the data preparation time for the extraction of the data from the readout buffers, shown in Fig. 5b, and the algorithmic CPU time, shown in Fig. 5c. Since the L2 algorithm executes on a per RoI basis, the time per event is determined by the time for processing a single RoI and the number of RoIs in the event. The full algorithm time for a single RoI has a mean of 6.5 ms and a long tail extending to approximately 80 ms, corresponding to an algorithm processing mean time of 2.8 ms and a combined data preparation and readout time with a mean of 3.7 ms which also provides the long tail.

**Fig. 6:**
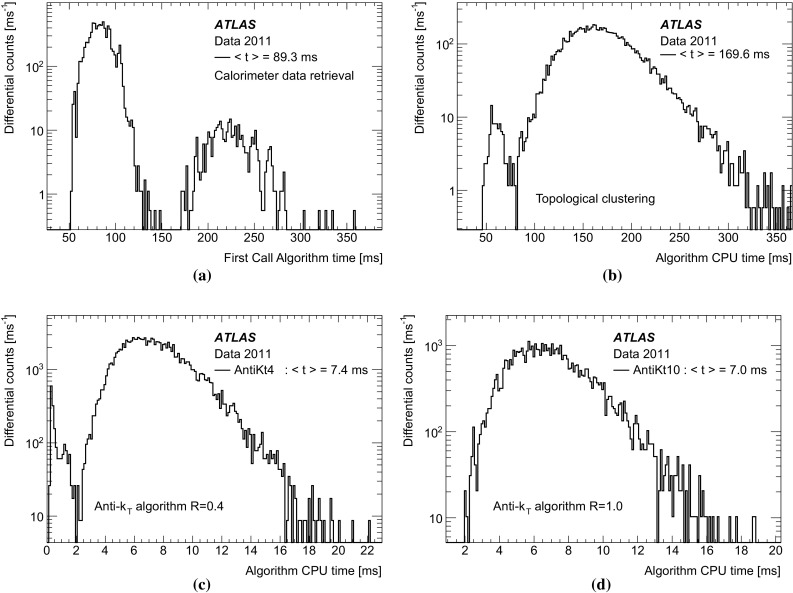
The processing times for the Event Filter jet trigger: **a** the time for the data preparation for the full calorimeter data; **b** the processing time for the topological clustering; **c**, **d** the times for the actual jet finding for the anti-$k_{t}$ algorithm, for instances with radius parameters **c** $R=0.4$, and **d** $R=1.0$

The execution time of the algorithm per event, rather than per RoI shows a clear peak around 6 ms and a second peak around 12 ms, due to events containing two RoIs. The data preparation time for the full event has a peak at around 3 ms for single RoI events and another from the two-RoI events around 6 ms. The algorithm CPU time has corresponding peaks around 4 and 8 ms.

At the EF, following the data preparation steps – the retrieval of calorimeter cell information from memory and building of topological clusters – a single instance of the anti-$k_{t}$ jet finder is executed for each of the required jet radii, $R=0.4$ and $R=1.0$. The times for each of the data preparation stages and the two jet radii are shown in Fig. 6, for the same 2011 run. For the data retrieval stage, before the topological clustering, two distinct peaks are observed. The first, with a mean around 80 ms, represents the processing of the complete event. The second broader peak, with a mean of approximately 220 ms, is due to an artefact of the trigger processing by the EF farm: each CPU in the EF farm runs a separate instance of the EF software and performs some additional initialisation for the first event each receives, increasing the processing time for the first event processed by each CPU node. The number of events in this second peak then corresponds to the number of individual CPUs in the farm. The most time consuming part of the full EF processing is the topological clustering, with a mean of approximately 170 ms. The jet finding itself is comparatively fast, requiring approximately 7 ms per instance. Due to the different prescales and thresholds used for the triggers for the different jet radii, the event topology and $E_\mathrm{T}$ spectrum is slightly different for the events processed by each instance of the anti-$k_{t}$ algorithm, resulting in the slightly different distributions seen in Figs. 6c, d. The peaks seen at short times in the topological clustering and $R=0.4$ anti-$k_{t}$ jet finding are due to the low threshold EF triggers seeded by the random trigger at L1 which therefore may contain fewer calorimeter cells with significant energy and so do not take as long to process. The anti-$k_{t}$ jet finding using $R=1.0$ only processes events seeded by jets found both at L1 and L2, where these jets pass the 95 GeV L2 threshold, so this peak is largely absent in Fig. 6d.

After the jet finding has completed, the selection hypothesis algorithms are executed both at L2 and the EF. For the single inclusive and multi-jet triggers the hypothesis algorithm typically executes in approximately 10 $\mu$s for each signature.

## Comparison of trigger and offline performance

An important concern for the trigger reconstruction is the producion of objects resembling as closely as possible those later reconstructed offline, to allow informed event selection with high efficiency while minimising any increase in the trigger rate. This is achieved by reducing any finite trigger–offline resolution or bias so that any selection of objects on the basis of trigger quantities more closely corresponds to the offline selection used for physics analyses. For this reason, the performance of the jet trigger during 2011 data taking has been evaluated with respect to the offline jet reconstruction. The offline reference jets have been reconstructed using the infrared and collinear safe anti-$k_{t}$ algorithm [14] implemented in the FASTJET [15] package. The same values of the radius parameter are used offline: $R = 0.4$ for the standard analyses, and $R=1.0$ used for the analysis of boosted objects.

The trigger performance is defined in terms of specific metrics, comparing offline and trigger reconstructed jets, such as jet selection efficiency, and the transverse energy or angular resolution with respect to offline jets. Comparisons of the same metrics with Monte Carlo simulated samples are shown in this and the following sections to illustrate how well the simulation describes the data and where disagreements appear. It should be emphasised, however, that the focus is on performance indicators determined from collision data, and detailed comparisons of different simulation configurations are beyond the scope of this paper.

### Data samples and event selection

It is informative to evaluate the performance of the trigger in simulated events and compare it to the real trigger running in collision data. The ATLAS trigger simulation runs exactly the same code for the HLT as that run online, and a very precise emulation for L1. The differences observed between collision data and simulation are due either to differences in the underlying physics, such as the composition and internal topology of the jets themselves, or to the kinematics, hadronisation, treatment of underlying event, or to differences in the simulation of the detector response or the detector conditions.

Because of these potential sources of differences between data and simulation, for jet physics analyses, trigger selection and trigger related calibrations are generally obtained using the data rather than relying on the trigger performance from the Monte Carlo simulation. Therefore, while it is desirable for the simulation to accurately reproduce the behaviour of the trigger, it is by no means essential.

For the evaluation of the trigger performance, events are selected from those written offline that are free from known problems with the detector or beam conditions. From these events, offline reconstructed jets which satisfy standard ATLAS jet selection criteria used in physics analyses [4–6] are selected to provide a reference jet sample. Besides the kinematic selection, these criteria also include jet-quality selections [10, 30, 31] to reject fake jets reconstructed from non-collision signals, such as beam-related background, cosmic rays or detector noise. Similar jet quality criteria are applied online to the trigger jets.

The efficiency for each specific chain has been evaluated using events selected by an alternative chain which is unbiased by the selection of the chain being evaluated. Therefore, wherever possible, the efficiencies have been evaluated using trigger chains seeded by a random trigger at L1, passing through L2 and EF without additional trigger selection. Where this was not possible, the standard chains have been used, but selecting only those *pass through* events, where the trigger accepted the event irrespective of the trigger jet selection, as discussed in Sect. 3.

There are a number of general purpose event generators for LHC physics: for more complete review, see elsewhere [32]. In the following studies, data are compared with simulated events produced using either the Herwig [33] or Pythia [34] Monte Carlo generators. Each simulates complete physics events using a hard subprocess with a leading logarithmic parton shower followed by a soft hadronisation model to generate the outgoing hadrons. Both include models for the underlying event: In Herwig, the formation of hadrons from the final state quarks and gluons produced in the parton shower is described using a cluster hadronisation model [35], whereas the Pythia generator uses the Lund string fragmentation model [36, 37].

In the following discussion the central and the forward jets triggers are discussed separately. For central jet triggers in the range $|\eta _{\mathrm {jet}}|<3.2$, offline jets are required to lie in the range $|\eta _{\mathrm {jet}}|<2.8$ in order to completely contain jets with radius parameter 0.4. Similarly, for the forward jet triggers, which lie in the range $3.2<|\eta _{\mathrm {jet}}|<4.9$, offline jets satisfying $3.6<|\eta _{\mathrm {jet}}|<4.5$ are required.

For offline analyses, jets are corrected for the difference between electromagnetic and hadronic responses in the calorimeter. Therefore jets can be defined either at the electromagnetic (EM) scale, which correctly measures the energy deposited by electromagnetic showers in the calorimeter, or after the full hadronic jet energy scale (JES) calibration [31, 38]. In the trigger, the JES calibration was not applied in 2011 since the full calibration was not available during data taking. The standard calibration of the reference offline jets is referred to as EM+JES, meaning jets built from (electromagnetic-scale) topological clusters, with jet energy corrected by the application of the JES calibration.

### Jet trigger performance metrics

Descriptions of the metrics used to assess the jet trigger performance can be found in this section: specifically for the efficiency measurement, and the evaluation of the resolution and bias arising from any offset between the trigger and offline reconstructed quantities.

#### Efficiency definition

Unless otherwise stated, the inclusive single jet efficiencies presented in this paper are of the form of *per jet* efficiencies with respect to the corresponding jet reconstructed offline. This represents the probability that an offline jet will have a corresponding jet reconstructed in the trigger that satisfies the trigger selection. Efficiencies *per event* can also be defined, in terms of global event properties, such as the $E_\mathrm{T}$ of the leading jet in the event. These are more sensitive to the event topology and more difficult to interpret, since, for example, any other jet might cause the event to be accepted, even if the leading offline jet does not. For a multi-jet trigger however, where the selection depends on the properties of many jets, these *per event* selections may be very informative; this is discussed further in Sect. 5.4.3.

The jet reconstruction efficiency, $\varepsilon$, for a sample of jets can be defined as the ratio of the number of offline jets, *N*, passing some selection which defines the sample, and the number of those jets, *m*, which are also reconstructed in the trigger to within some appropriate matching criteria, such that $\varepsilon \equiv m/N$.

**Fig. 7:**
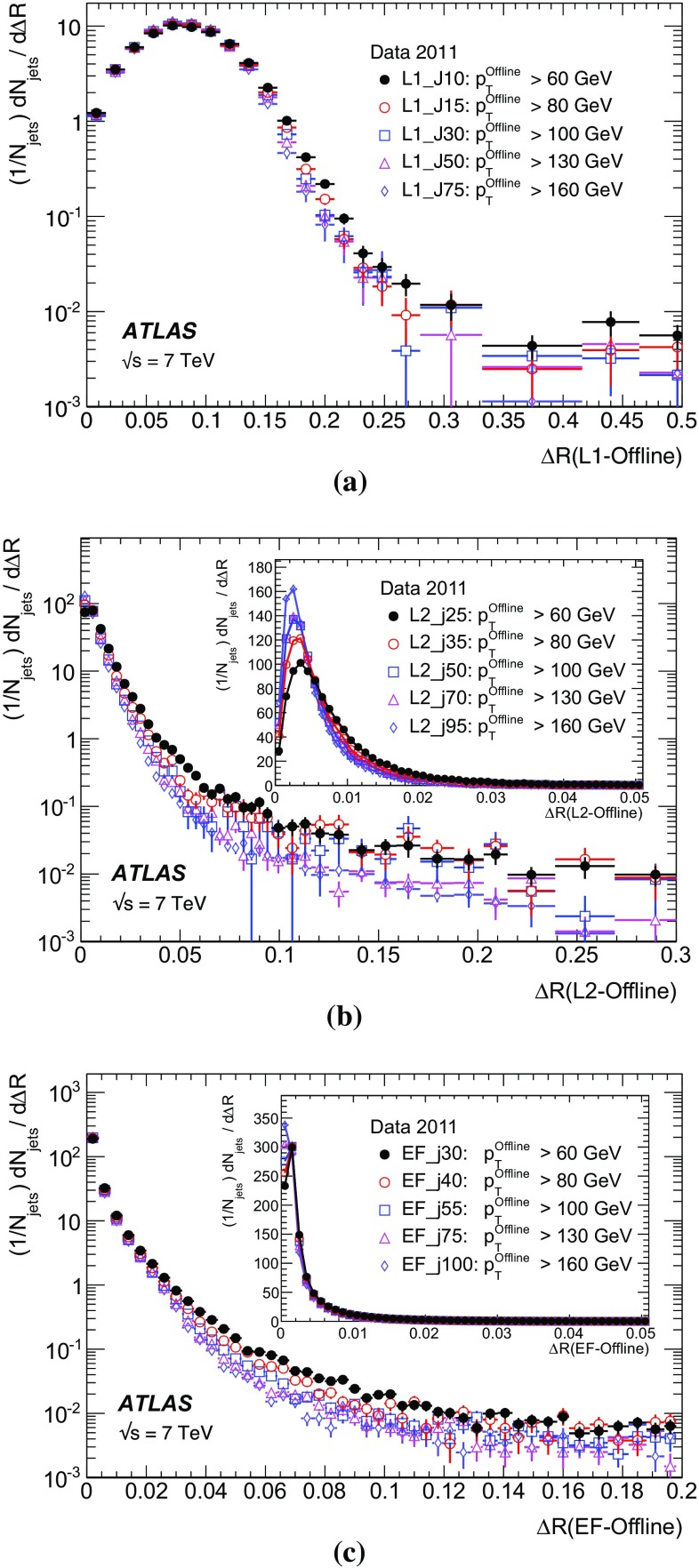
The distribution of $\Delta R$ between the offline jets and the closest matching trigger jet: **a** for L1; **b** for L2; **c** for the EF. In each case the differences are shown with respect to offline jets above the $p_{\text {T}}$ thresholds indicated such that the trigger for each threshold is fully efficient. Statistical uncertainties only are shown

The choice of matching criteria must be considered as an important aspect of the definition of the efficiency, since a tighter matching will necessarily result in a lower efficiency and *vice versa*. This is important since the correspondence of offline jets to trigger jets is not one-to-one.

The binned differential efficiency, $\varepsilon _i$, in some generic variable $x_{\mathrm {jet}}$, where $x_i \le x_{\mathrm {jet}} < ( x_i+\Delta x )$, is defined analogously,

4 $$\begin{aligned} \varepsilon _i = \frac{m(x_i \le x_{\mathrm {jet}}< (x_i+\Delta x) )}{N(x_i \le x_{\mathrm {jet}} < ( x_i+\Delta x) ) }. \end{aligned}$$

The criterion applied here for matching online and offline jets is based on the distance $\Delta R = \sqrt{ (\Delta \eta )^2+(\Delta \phi )^2}$ in the $\eta$–$\phi$ plane between the offline jet and the closest matching trigger jet.

Figure 7 shows the distribution of $\Delta R$ for trigger jets from L1, L2 and the EF. Distributions are shown for each trigger for several different $p_{\text {T}}$ ranges such that the trigger in each case is fully efficient. For the L2 and EF $\Delta R$ distributions, the agreement between online and offline clearly improves with increasing jet $E_\mathrm{T}$. Since the L1 trigger uses only coarse granularity calorimeter information, and quantises the $\eta$ and $\phi$ directions to the nearest 0.2, the resolution in $\eta$ or $\phi$ from L1 would be expected to be approximately 0.06. For similar Gaussian distributed residuals in $\eta$ and $\phi$ this would result in a maximum in the $\Delta R$ distribution of around 0.08, as observed in Fig. 7a. Although the L2 trigger operates only within each RoI, it uses calorimeter information at the full detector granularity. Therefore the jet $\eta$ and $\phi$ reconstruction in Fig. 7b for L2 is significantly improved with respect to that seen in Fig. 7a for L1. The EF uses the same topological clustering algorithm and the same jet finding algorithm as the offline reconstruction. This leads to a further improvement in the resolution between the trigger and offline jets for the EF with respect to what is already acheived at L2, and can be seen in Fig. 7c.

For the matching used to define the resolution and efficiency, criteria in $\Delta R$ have been chosen which allow high efficiency for genuine matches while reducing the contribution from random matches that may degrade the resolution. For the analyses of the efficiency and resolution for single jets presented here, trigger jets are required to match with the closest offline jet to within $\Delta R<0.4$ at L1, and to within $\Delta R<0.2$ for L2 and the EF.

#### Trigger efficiency behaviour near threshold

The trigger system selects jets based largely on the $E_\mathrm{T}$ and pseudorapidity of the jets reconstructed at the three trigger levels. The principle source of difference between the $E_\mathrm{T}$ of offline and trigger jets in 2011 was the hadronic calibration, which was not applied online in this period. Smaller differences at the different levels arise from the detector granularity at L1, the input objects to the jet algorithms and the L2 algorithms. These differences give rise to shifts and additional resolution smearing of the $E_\mathrm{T}$ reconstructed in the trigger with respect to that reconstructed offline. The selection efficiencies for the various trigger levels resulting from these shifts and smearing are therefore not step functions when measured as a function of the offline $E_\mathrm{T}$. Instead, the efficiency as a function of $E_\mathrm{T}$ will exhibit a more slowly rising edge as the trigger turns on. This has an impact on ATLAS physics analyses; in general, a steeply rising efficiency near the $E_\mathrm{T}$ threshold which rapidly approaches a plateau near 100 % efficiency indicates good performance of the trigger. A more slowly rising efficiency, or one which does not saturate near 100 % can be problematic for offline data analysis as it has the potential to introduce large systematic uncertainties in the selection efficiency and background estimates.

A more slowly rising edge is expected at L1 due to the poorer $E_\mathrm{T}$ resolution, arising from the coarse granularity data. Care must therefore be taken to ensure that the L1 efficiency reaches its plateau for $E_\mathrm{T}$ values below the onset of the rising edges of the L2 and EF efficiency curves. The thresholds for the higher trigger levels therefore impose upper limits on the corresponding L1 thresholds, and so reduce the efficacy of raising these thresholds in order to reduce the rate of L1 accepted events. Because of the steeply falling $p_{\text {T}}$ spectrum this implies that more events need to be accepted at L1 to avoid reducing the EF efficiency significantly at higher $E_\mathrm{T}$. A more steeply rising efficiency is expected at the EF due to the improved $E_\mathrm{T}$ resolution and the greater similarity in the reconstruction algorithms used online and offline. To minimise systematic uncertainties associated with the trigger, most ATLAS physics analyses relying on jet triggers require that the offline $E_\mathrm{T}$ for selected jets lie in the *efficiency plateau* region, where the efficiency is above 99 %.

#### Definition of transverse energy resolutions and offsets

The transverse energy resolutions and offsets are computed from the distributions of the residuals between the quantities computed offline and at trigger level.

To provide a single statistic to parameterise the resolution, the root-mean-square (RMS) deviation of the central 95 % of the residual distribution is used. This is further divided by the RMS for the central 95 % of a Gaussian distribution with unit standard deviation. In this way, if the distribution were Gaussian, the normal Gaussian resolution would be obtained. This measure was chosen because the RMS of the full distribution can be strongly biased by significant non-Gaussian tails. Similarly, a measure for the resolution based on the width of a Gaussian distribution fitted over the central region of the distribution will fail to take into account a significant fraction of the distribution if there are large tails and will not be representative of the actual performance.

Offsets and resolutions between jets reconstructed in the trigger and reconstructed offline are obtained from the distribution of the quantity

5 $$\begin{aligned} \frac{E_{\mathrm T}^{\mathrm {Trigger}}-E_\mathrm{T}^{\,\mathrm{Offline}}}{E_\mathrm{T}^{\,\mathrm{Offline}}}. \end{aligned}$$

For a comparison of offline, fully calibrated, jets with the trigger jets reconstructed at the electromagnetic scale, the transverse energy offset will be large. Therefore, results are first presented in terms of the jet definitions actually used by offline analyses and the online systems, and then also shown for the case of comparison of the online jets with the offline jets, both reconstructed at the electromagnetic scale, which more closely resemble each other.

### Transverse energy offsets and resolutions

Understanding of the offsets and resolutions in the data is useful for the determination of the behaviour of the trigger efficiencies near the $E_\mathrm{T}$ thresholds, since the offset and resolution will, respectively, have an impact on the position and gradient of the rising edge.

As the resolutions and offsets presented in this section are with respect to offline jets at the EM+JES scale, large offsets are expected. For brevity, only the performance of the EF trigger is presented as this corresponds most closely to the offline reconstruction. Since physics analyses generally use $p_{\text {T}}$, where applicable, the offset and resolution of the quantity actually reconstructed in the trigger – namely $E_\mathrm{T}$ – are shown as a function of the offline jet $p_{\text {T}}$.

**Fig. 8:**
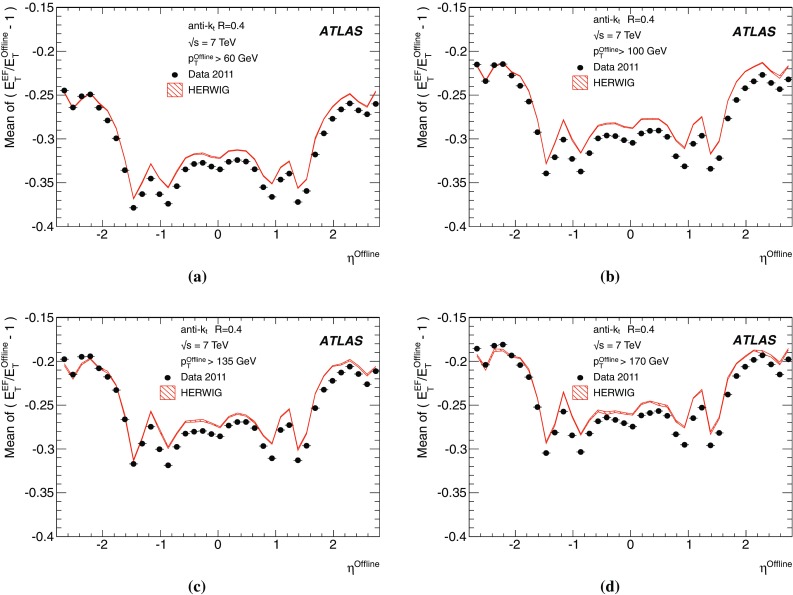
The mean relative offset for the EF trigger jets with respect to offline jets at the EM+JES energy scale as a function of the offline jet $\eta$ in four different ranges of jet $p_{\text {T}}$: **a** $p_\mathrm{T}^\mathrm{Offline}$ $>60$ GeV; **b** $p_\mathrm{T}^\mathrm{Offline}$ $>100$ GeV; **c** $p_\mathrm{T}^\mathrm{Offline}$ $>135$ GeV; and **d** $p_\mathrm{T}^\mathrm{Offline}$ $>170$ GeV. Statistical uncertainties only are shown: the data are shown as the solid points with error bars, and the Herwigsimulated sample as the hatched band.

In order to ensure that the trigger has reached plateau efficiency for the lowest L1 jet $E_\mathrm{T}$ threshold, the performance in terms of reconstruction of the jet transverse energy and pseudorapidity is presented for offline jets with $p_{\text {T}} >60$ GeV when evaluating the central jet triggers seeded by the L1 jet trigger, and $p_{\text {T}} >50$ GeV when evaluating the forward jet trigger.

#### Central jets

Figure 8 shows the mean relative offset as a function of the offline jet $\eta$ for both the data and the Herwig simulated sample integrated over $p_{\text {T}}$ above four different thresholds, indicated in the figure. The general trend of the data is reasonably well reproduced by the Monte Carlo simulation, with small differences at the percent level. As discussed previously, differences between the simulation and data result from inaccuracies or approximations in the simulation of the detector response or in the application of the detector conditions, but also from differences in the underlying kinematics and $p_{\text {T}}$ spectrum of the Monte Carlo sample.

A large $\eta$ dependence can be discerned: at low $p_{\text {T}}$, negative offsets of between 24 and 27 % in the endcaps, and between 32 and 35 % in the barrel are observed. This variation with $\eta$ is largely determined by the detector geometry and the different performance of the respective calorimeter subsystems – notably with larger differences in the transition (crack) regions between the barrel and endcap subsystems, around $|\eta |\sim 1.5$, which are populated with detector services and around $|\eta |\sim 0.8$ where there is a 20 % reduction in the depth of active material in the LAr calorimeter with respect to more central pseudorapidities. For the same minimum offline $p_{\text {T}}$ requirement, jets at higher $\eta$ values also have higher energy, which may also contribute to the observed differences in the endcap response when compared to more central pseudorapidities. These effects are largely accounted for in full calibration for the offline jets, but not for the trigger jets, where this correction was not applied in 2011. Differences in the detector conditions between online and offline reconstruction, such as information on masked, or inactive front end boards, which is only obtained following the offline calibration, also play a rôle. This can be seen in the small asymmetry observed between the forward and rear barrel regions for $|\eta |<0.6$, where the simulation, which includes these effects, broadly follows the trend seen in data, albeit with small quantitative differences. Larger offsets are seen in the crack regions due to the greater energy loss in the additional dead material in front of the calorimeter. These effects, including changes in the detector conditions occurring during data taking, are corrected for in the offline reconstruction using the full calibration.

The relative offset in the data is in general slightly more negative than that shown by the Monte Carlo simulation. The size of the offset of the EF trigger jets with respect to offline jets tends to decrease for the higher $p_{\text {T}}$ selections. This is largely attributable to the comparitively reduced energy loss in inactive material for jets of high energy when compared to those with lower energy. This trend is also fairly well reproduced by the simulation

**Fig. 9:**
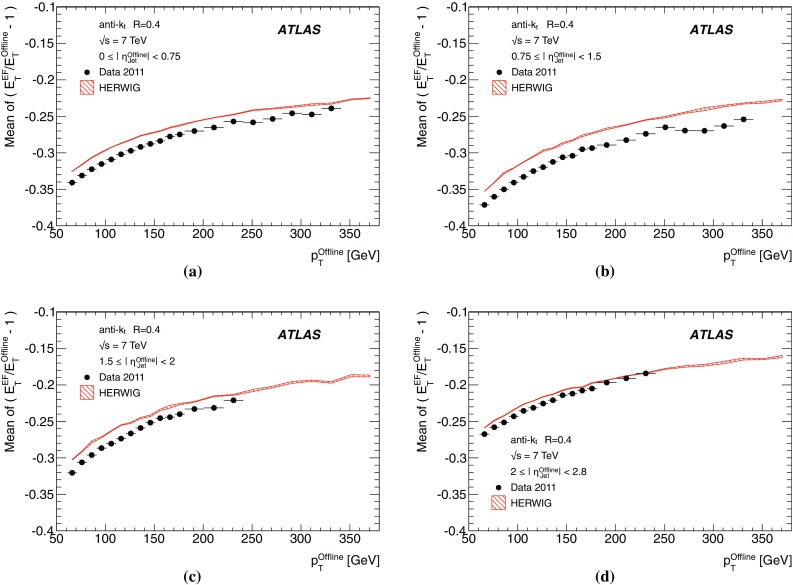
The mean relative offset for the EF trigger jets with respect to offline jets at the EM+JES energy scale as a function of the offline jet $p_{\text {T}}$, in four different regions of offline jet pseudorapidity: **a** $|\eta ^\mathrm{Offline}| < 0.75$; **b** $0.75\le |\eta ^\mathrm{Offline}| < 1.5$, **c** $1.5\le |\eta ^\mathrm{Offline}| < 2$; and **d** $2\le |\eta ^\mathrm{Offline}| < 2.8$. Statistical uncertainties only are shown: the data are shown as the solid points with error bars, and the Herwigsimulated sample as the hatched band.

In Fig. 8 the offset in transverse energy from the Event Filter, $E_\mathrm{T}^\mathrm{EF}$, with respect to the offline jets is seen to vary over a range of approximately 10 % with $\eta$, with the offsets themselves being only two or three times larger than this range. Therefore the widths of the distributions of residuals obtained when integrating over the entire $\eta$ range would result from a convolution of the true resolution and the variation of the offset with $\eta$. The consequent resolution would appear artificially large, smeared by this additional factor of 10 %.

As a result, the mean offset and resolution in $E_\mathrm{T}$ as a function of $p_{\text {T}}$ has been measured in four separate regions of $|\eta ^\mathrm{Offline}|$;

6 $$\begin{aligned} 0~\le&|\eta ^\mathrm{Offline}|&< ~0.75 \end{aligned}$$

7 $$\begin{aligned} 0.75~\le&|\eta ^\mathrm{Offline}|&< ~1.5 \end{aligned}$$

8 $$\begin{aligned} 1.5~\le&|\eta ^\mathrm{Offline}|&< ~2 \end{aligned}$$

9 $$\begin{aligned} 2~\le&|\eta ^\mathrm{Offline}|&< ~2.8, \end{aligned}$$

with the first and last corresponding to those regions where the offset is approximately constant in the barrel and endcap regions, respectively. In the remaining two regions the offset varies rapidly due to the crack regions in the calorimeters. The resulting offsets are shown as a function of $p_\mathrm{T}^\mathrm{Offline}$ in Fig. 9. It is seen that the mean offset decreases as the offline jet $p_{\text {T}}$ increases, with the smallest offset in the endcap regions, as expected from the variation of the offset with $\eta ^\mathrm{Offline}$ seen in Fig. 8. Again, as can be seen in Fig. 9, the simulation underestimates the offsets for all $|\eta ^\mathrm{Offline}|$, by aproximately 1 % for $|\eta ^\mathrm{Offline}|<0.75$ and 2 % for the regions $0.75<|\eta ^\mathrm{Offline}|<2$. This is also true, albeit to a lesser degree, for the range $2\le |\eta ^\mathrm{Offline}|<2.8$, since the positive and negative pseudorapidity regions seen in Fig. 8 have been combined.

**Fig. 10:**
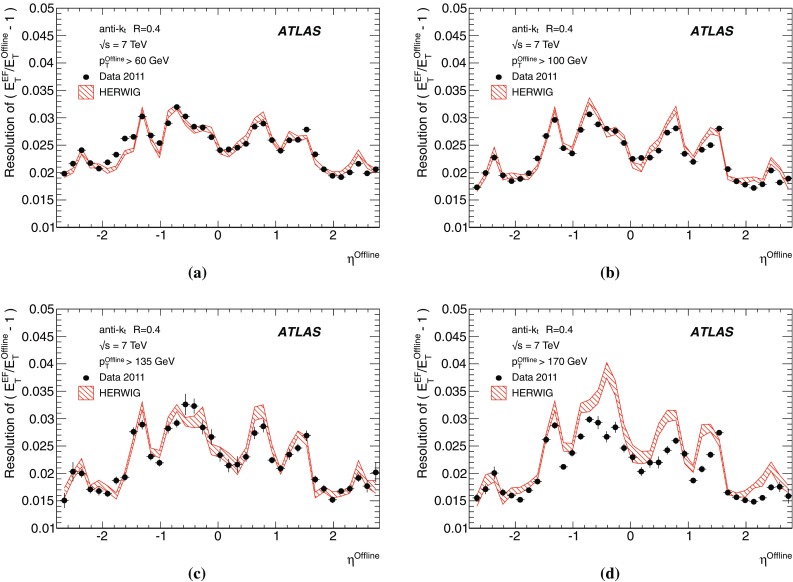
The resolution for the EF trigger jets with respect to offline jets at the EM+JES energy scale as a function of the offline jet $\eta$ in four different ranges of jet offline $p_{\text {T}}$: **a** $p_\mathrm{T}^\mathrm{Offline}$ $>60$ GeV; **b** $p_\mathrm{T}^\mathrm{Offline}$ $>100$ GeV; **c** $p_\mathrm{T}^\mathrm{Offline}$ $>135$ GeV; and **d** $p_\mathrm{T}^\mathrm{Offline}$ $>170$ GeV. Statistical uncertainties only are shown: the data are shown as the solid points with error bars, and the Herwigsimulated sample as the hatched band.

Because of the large dependence of the offset on $p_{\text {T}}$, when integrating the distribution over $p_\mathrm{T}^\mathrm{Offline}$ to show for example the variation of the resolution as a function of $\eta ^\mathrm{Offline}$, this will include the convolution of the resolution from the detector response with the variation of the offset with $p_\mathrm{T}^\mathrm{Offline}$ itself. This should be taken into account when estimating the resolution. This effect will, however, not be as pronounced as in the case of the similar variation of the offset with $\eta ^\mathrm{Offline}$, because of the steeply falling $p_{\text {T}}$ spectrum, so the shape of the residual distributions will be largely determined by the jets near the $p_\mathrm{T}^\mathrm{Offline}$ threshold.

Figure 10 shows the resolution versus $\eta ^\mathrm{Offline}$ for the four $p_{\text {T}}$ ranges shown earlier. The resolution is generally better in the endcap regions than in the barrel and does not vary greatly between the four $p_{\text {T}}$ ranges, although it varies sharply as a function of $\eta ^\mathrm{Offline}$. The resolution is quite well described by the simulation where, as in data, it does not vary greatly with $p_{\text {T}}$. The large asymmetry in the resolution between the barrel regions at positive and negative $\eta$ due to the detector conditions is approximately reproduced by the simulation. At high $p_{\text {T}}$, the simulation predicts a worse resolution than seen in the data, by up to 0.5 % or slightly higher in some regions. In the more forward directions, the better $E_\mathrm{T}$ resolution partly results from the larger jet energy relative to jets in the barrel with the same $E_\mathrm{T}$.

**Fig. 11:**
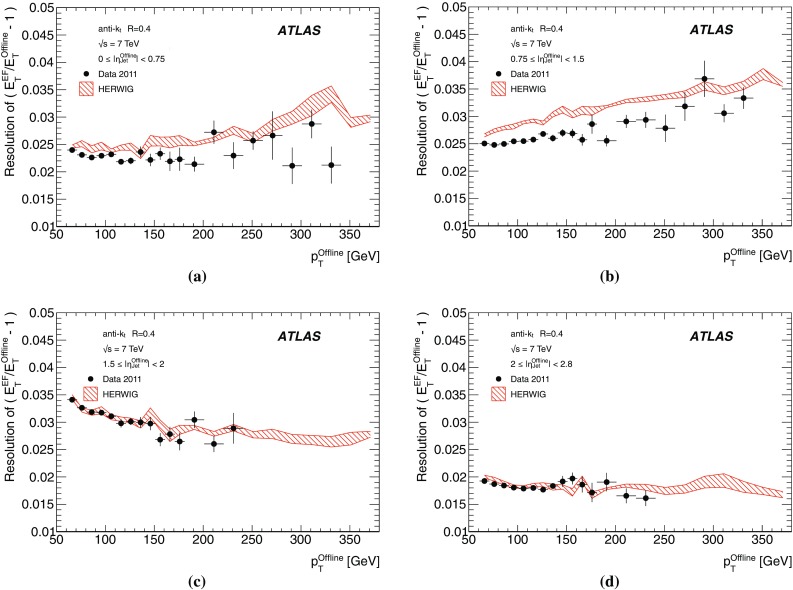
The resolution for the EF trigger jets with respect to offline jets at the EM+JES energy scale, as a function of the offline jet $p_{\text {T}}$, in four different regions of offline jet pseudorapidity: **a** $|\eta ^\mathrm{Offline}| < 0.75$; **b** $0.75\le |\eta ^\mathrm{Offline}| < 1.5$; **c** $1.5\le |\eta ^\mathrm{Offline}| < 2$; and **d** $2\le |\eta ^\mathrm{Offline}| < 2.8$. Statistical uncertainties only are shown: the data are shown as the solid points with error bars, and the Herwigsimulated sample as the hatched band.

Figure 11 illustrates the resolution as a function of transverse energy in the same four $\eta ^\mathrm{Offline}$ ranges discussed previously. In the barrel region, at low $p_\mathrm{T}^\mathrm{Offline}$, the resolution is approximately constant. The resolution found in collision data is again not fully reproduced by simulation, although the differences found are small, being only around 0.5(% at most. In the crack regions, as might be expected from the larger energy loss indicated by the larger offsets observed earlier, the resolutions are worse than in the barrel or endcaps and show a larger dependence on the $p_{\text {T}}$.

#### Forward jets

**Fig. 12:**
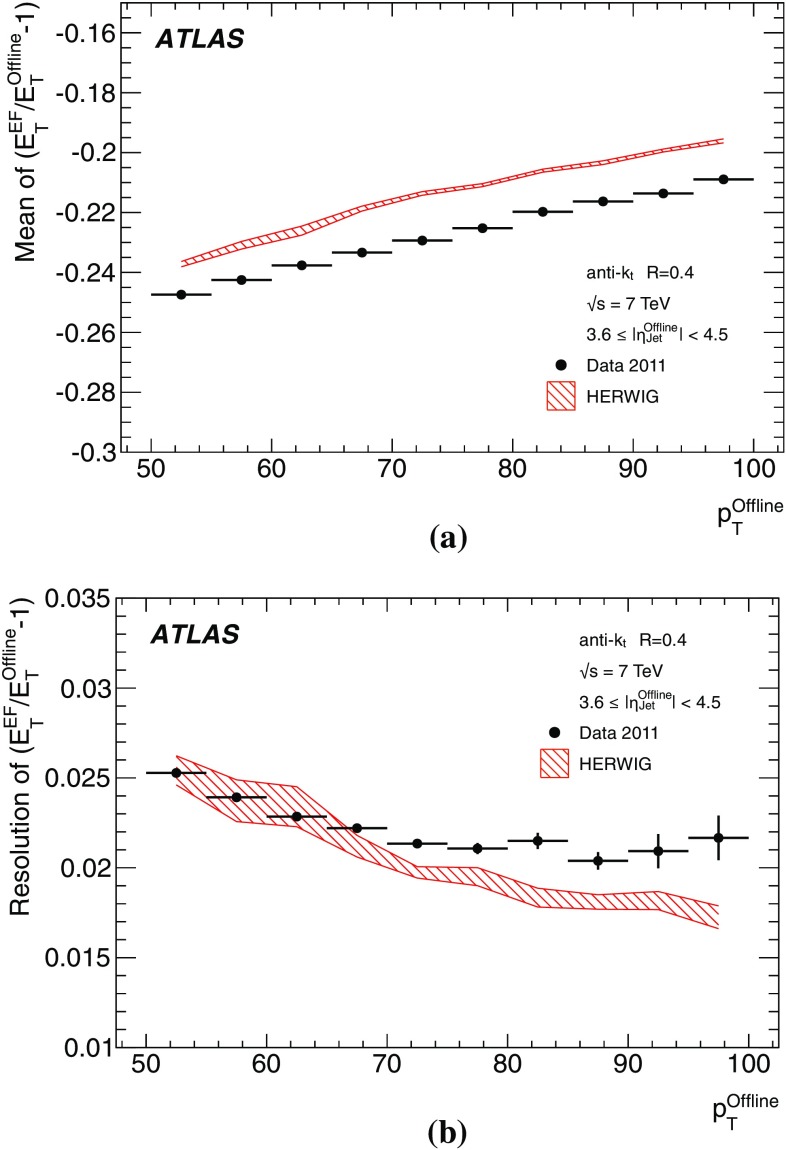
The performance for the EF forward trigger jets with respect to offline jets at the EM+JES energy scale as a function of the offline jet $p_{\text {T}}$ for jets with $3.6<|\eta ^\mathrm{Offline}|<4.4$: **a** The mean relative offset; and **b** the resolution. Statistical uncertainties only are shown: the data are shown as the solid points with error bars, and the Herwigsimulated sample as the hatched band.

The offsets and resolutions with respect to offline jets as a function of offline $p_{\text {T}}$ for jets from the forward jet trigger are shown in Fig. 12a, b respectively. The offline jets are produced with a radius parameter $R=0.4$ and are required to be within the range $3.6< |\eta ^\mathrm{Offline}| < 4.4$. These show a dependence of the offset on the jet $p_{\text {T}}$ which improves towards high $p_{\text {T}}$ as for the central jets, from approximately 24 % at lower $p_{\text {T}}$ to only 20 % at high $p_{\text {T}}$, broadly consistent with the behaviour seen in the central jet trigger. The simulation shows approximately 1% smaller offsets than seen in the data over the full $p_{\text {T}}$ range. The jet resolution is reasonably well described by the simulation at low $p_{\text {T}}$, with larger differences at high $p_{\text {T}}$, of less than 0.5 %. The data show only a small variation of the resolution with offline $p_{\text {T}}$, of between 2.0 and 2.5 %, whereas the simulation shows a slightly stronger dependence at higher $p_{\text {T}}$.

#### The performance with respect to offline EM scale jets

All of the above results compare electromagnetic-scale jets, as measured by the trigger, with offline reconstructed jets at the EM+JES scale. In this section, trigger jets are compared to offline jets reconstructed at the EM scale only, to better illustrate the correspondence between the reconstruction in the trigger and offline when the same calibrations are applied to both.^2^

**Fig. 13:**
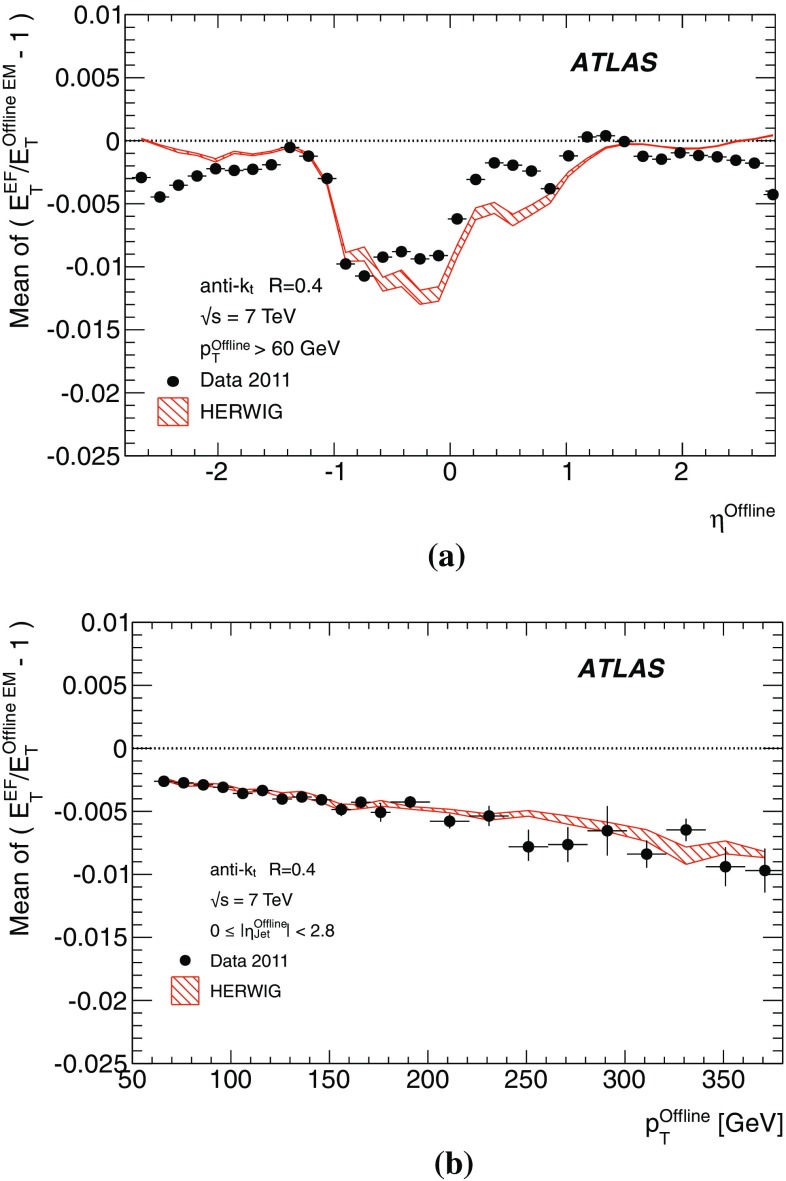
The mean relative offset for the EF trigger jets with respect to offline jets at the EM energy scale as a function: **a** of the offline jet $\eta$ for jets with $p_{\text {T}}$ $>60$ GeV; **b** as a function of the offline jet $p_{\text {T}}$ at the hadronic energy scale for jets in the range $|\eta ^{\mathrm {Offline}}|<2.8$. Statistical uncertainties only are shown: the data are shown as the solid points with error bars, and the Herwigsimulated sample as the hatched band.

Figure 13 shows the mean relative offset of the trigger jets at the EF with respect to offline jets at the EM scale as functions of the offline jet pseudorapidityand $p_{\text {T}}$ at the EM+JES scale. A mean offset of less than 0.5 % is observed at low $p_{\text {T}}$, increasing to around 1 % at higher $p_{\text {T}}$. This is in contrast to the much stronger dependence on $p_{\text {T}}$ seen for the offset when comparing to fully corrected offline jets from Fig. 9, where the offset varies from 35 % at low $p_{\text {T}}$ to 15 % at higher $p_{\text {T}}$. The offset as a function of offline jet pseudorapidity, integrated over the range $p_{\text {T}}$ $>60$ GeV, has a value of better than 0.5 % everywhere, except for the barrel region in the range $-1<\eta <0$, where the offset is approximately 1 %. This is again in stark contrast to the offsets found between EM scale trigger jets and EM+JES offline jets shown in Fig. 8, which extend to 35 %.

**Fig. 14:**
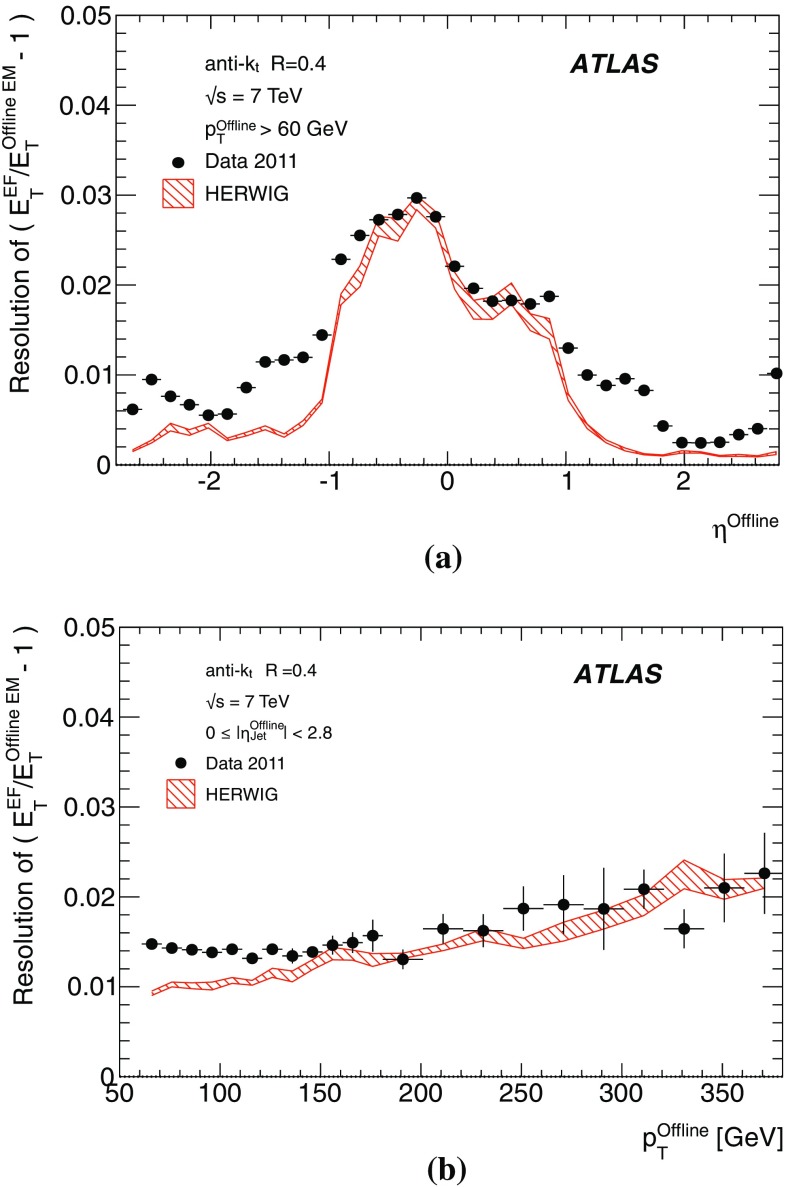
The resolution for the EF trigger jets with respect to offline jets at the EM energy scale: (a) as a function of the offline jet $\eta$ for jets with offline $p_{\text {T}}$ $>60$ GeV; (b) as a function of the offline jet $p_{\text {T}}$ at the hadronic energy scale for jets in the range $|\eta ^{\mathrm {Offline}}|<2.8$. Statistical uncertainties only are shown: the data are shown as the solid points with error bars, and the Herwigsimulated sample as the hatched band.

The resolutions as a function of the offline jet pseudorapidity and $p_{\text {T}}$ are presented in Fig. 14 for all jets with $p_\mathrm{T}^\mathrm{Offline}$ $>60$ GeV. Resolutions of approximately 1, 2 and 3 % are seen for $|\eta |>1$, and the positive and negative pseudorapidity regions in the barrel respectively. The resolution in the central barrel region is approximately the same as that with respect to fully corrected offline jets, but around 1 % better in the region $1<|\eta |<2$. As a function of $p_{\text {T}}$, the resolution degrades slightly from approximately 1.5 % at low $p_{\text {T}}$ to 2 % at higher $p_{\text {T}}$. The simulation describes the resolution reasonably well in the barrel region, but predicts significantly better resolution than in the data for the regions with $|\eta |>1$. The asymmetry between the positive and negative $\eta$ barrel regions observed when comparing with EM+JES jets is also observed here.

The difference in the offsets observed between the EM and EM+JES jets serves to illustrate the size of the correction applied during the JES calibration and suggests that, should the same correction be applied online, the correspondence between offline and trigger reconstructed jets would be better than a few percent with resolutions of better than 3 %. Since 2012 the JES calibration has been applied to the trigger jets online.

### Jet trigger reconstruction efficiency

To understand the performance of the trigger in more detail, the trigger efficiency versus $E_\mathrm{T}$ has been studied for all the major inclusive trigger chains, comparing once again to fully calibrated offline jets at the EM+JES scale. Here $E_\mathrm{T}$ is used as the variable of merit, since the trigger selects jets based on $E_\mathrm{T}$ rather than the $p_{\text {T}}$ used in physics analyses.

Measuring the efficiency in data requires events to be selected with an independent reference trigger which is unbiased with respect to the trigger being studied. The reference trigger is usually chosen to have a lower $E_\mathrm{T}$ threshold than the specific trigger being evaluated, for which the $E_\mathrm{T}$ region to be studied must lie well within the plateau region of the reference trigger. To study the very low $E_\mathrm{T}$ triggers, triggers selecting events randomly at L1 or pass through events without additional trigger selection have been used.

#### The single inclusive jet trigger efficiency

**Fig. 15:**
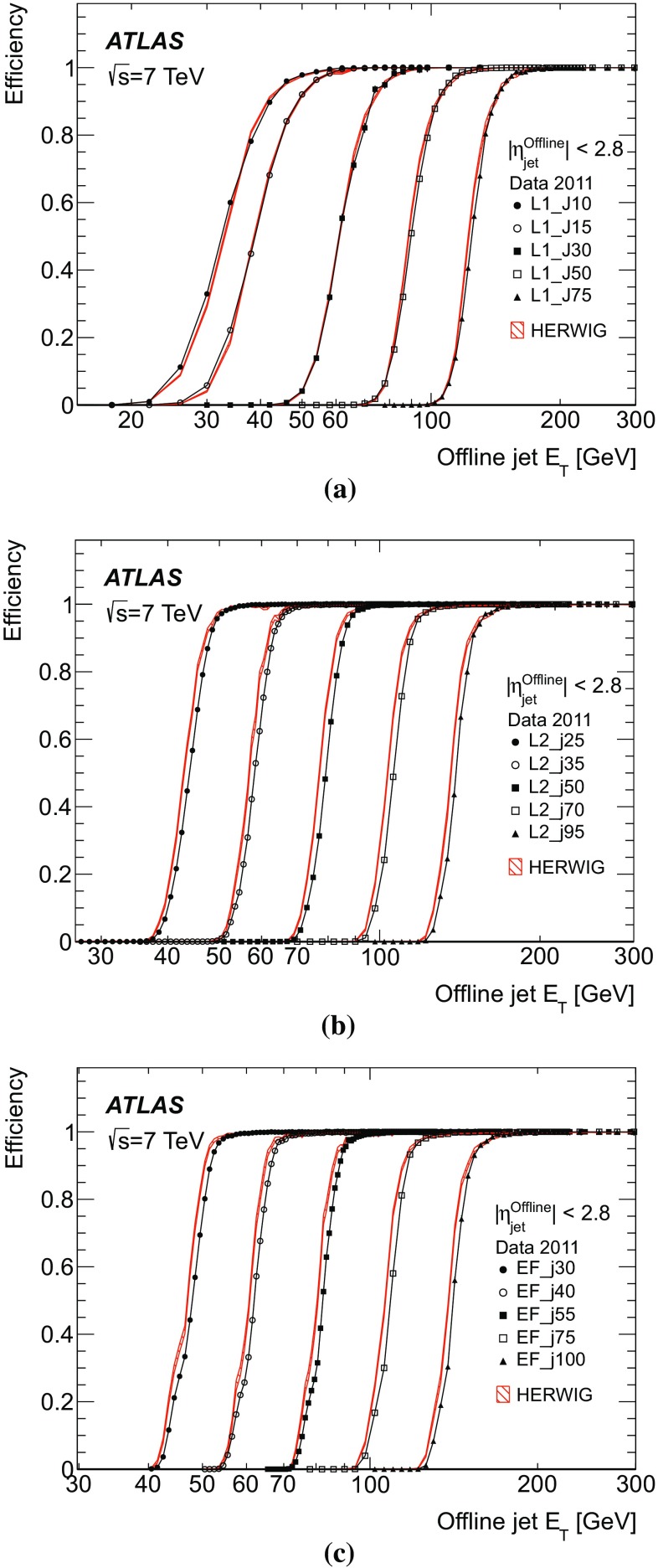
The efficiency as a function of offline jet $E_\mathrm{T}$ for various single inclusive jet triggers. Shown are the efficiency for data, and for the Herwig simulated sample for: **a** L1; **b** L2; and **c** the EF triggers. For data, the efficiency is computed with respect to events taken by an independent trigger that is 100 % efficient in the relevant region. Statistical uncertainties only are shown: the data are shown as the solid points with error bars, and the Herwigsimulated sample as the hatched band.

The efficiency curves for a selection of single inclusive jet triggers as a function of $E_\mathrm{T}$ are shown in Fig. 15 for data and simulation, for each of the three trigger levels. Relative trigger efficiencies are shown: the L2 trigger requires that a jet has already satisfied the L1 trigger in the chain; similarly an EF trigger requires that L2 has been satisfied. The rising edges for the L2 and the EF selection are considerably sharper than for the corresponding L1 selection due to the improved $E_\mathrm{T}$ resolution in the HLT. At all levels, any discrepancies between data and simulation are of the order of a few percent close to the full efficiency region.

In Sect. 5.3, the Monte Carlo simulation was seen to predict smaller offsets than the data at nearly all $p_{\text {T}}$. The result of this is that the trigger jets in the Herwig sample would have a correspondingly higher $E_\mathrm{T}$ than those from the data, and so the trigger would be expected to turn on earlier than the data.

**Fig. 16:**
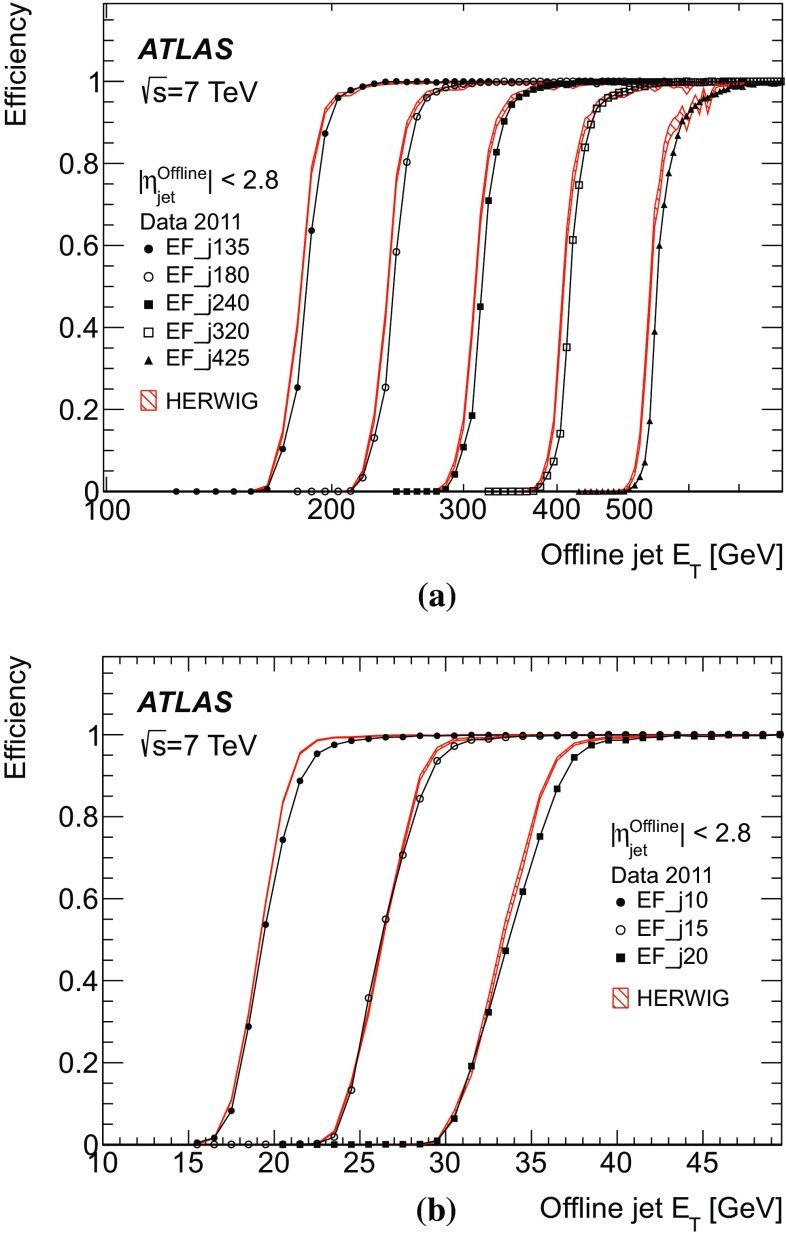
The efficiency for various EF triggers as a function of offline jet $E_\mathrm{T}$. Shown are the efficiencies for data and the Herwig simulated sample for the: **a** EF triggers seeded by L2_j95 and L1_J75; **b** EF triggers seeded by a random trigger at L1 and passed through L2. Statistical uncertainties only are shown: the data are shown as the solid points with error bars, and the Herwigsimulated sample as the hatched band.

The efficiencies as a function of $E_\mathrm{T}$ for additional EF triggers which ran in 2011 are shown in Fig. 16 for data and simulation. The high $E_\mathrm{T}$ threshold triggers are shown in Fig. 16a. The efficiencies as a function of $E_\mathrm{T}$ for the EF triggers seeded by a random trigger at L1 which are passed through L2 are shown in Fig. 16b. Since the random triggers require no jet selection at either L1 or L2, these EF triggers are unaffected by the coarse resolution and the less steep rising edge seen for the low threshold jet triggers at L1. This allows the triggers to reach their full efficiency at a lower $E_\mathrm{T}$ than is possible for the chains seeded by an L1 jet trigger. In this case, the lowest threshold trigger, with a transverse energy requirement of 10 GeV, is fully efficient by 25 GeV.

**Fig. 17:**
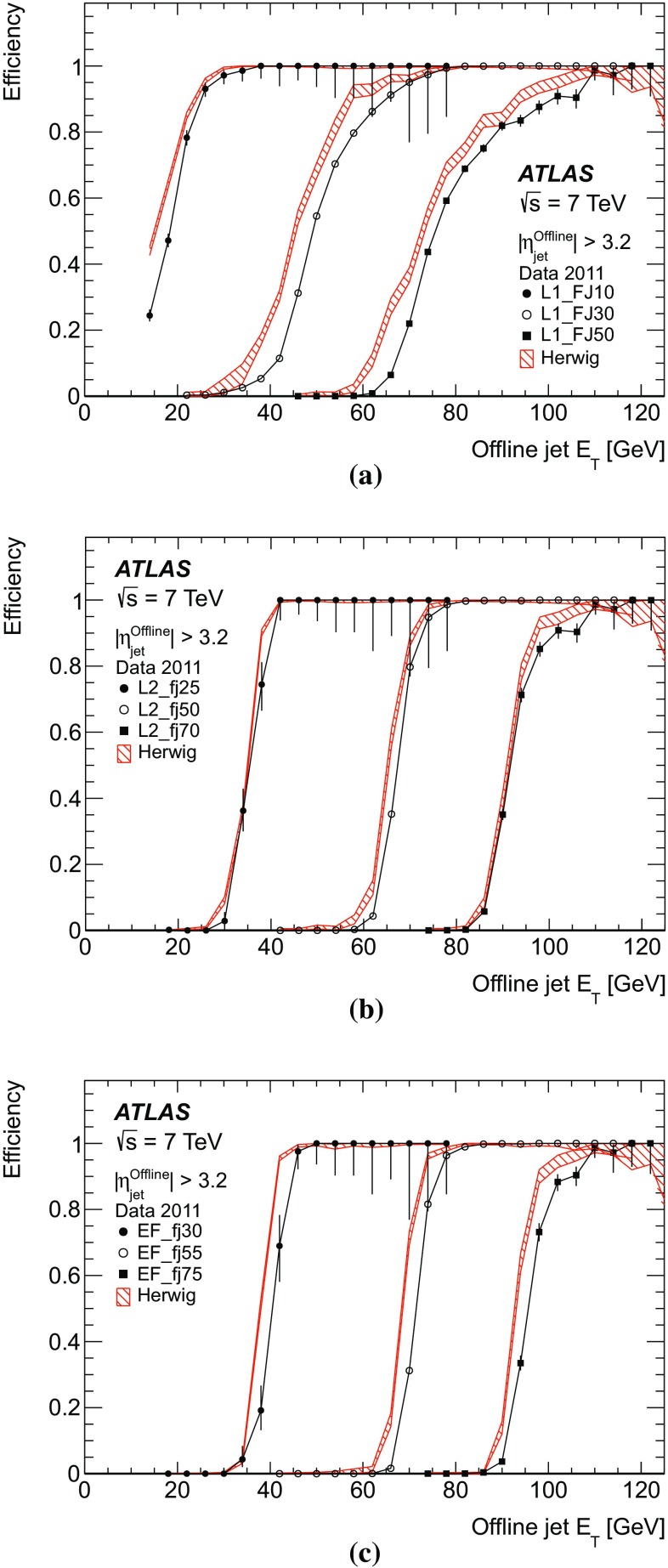
The efficiency for various forward jet triggers in data and the Herwig simulated sample as a function of offline jet $E_\mathrm{T}$ for: **a** L1_FJ10, L1_FJ30 and L1_FJ50; **b** L2_fj25, L2_fj50 and L2_fj70; **c** EF_fj30, EF_fj55 and EF_fj75. Statistical uncertainties only are shown: the data are shown as the solid points with error bars, and the Herwigsimulated sample as the hatched band.

Figure 17 shows the efficiency as a function of $E_\mathrm{T}$ for L1, L2 and EF jets in the *forward region*, defined as having a pseudorapidity $|\eta |> 3.2$. However, in order for these jets to be fully contained in the forward calorimeter the offline $|\eta |$ is required to be in the range $3.6 \le |\eta |\le 4.8$. The agreement between data and simulation is worse in the forward region than for central jets. This is related to the smaller offsets seen in simulation in Sect. 5.3.2 when compared to the data. This results in the trigger turning on at slightly lower $E_\mathrm{T}$ in the simulation than in the data.

#### Trigger efficiency versus pseudorapidity

The offset and resolution of the trigger, and the underlying kinematics, each affect the rising edge of the trigger efficiency as it increases towards plateau.

The resolution and offset of the trigger jets have been shown to vary significantly with pseudorapidity. This has a significant effect on the trigger efficiency and introduces a strong dependence on the pseudorapidity, of both the position of the midpoint and the sharpness of the rising edge of the trigger, and of the $E_\mathrm{T}$ at which the trigger reaches its maximal plateau efficiency.

**Fig. 18:**
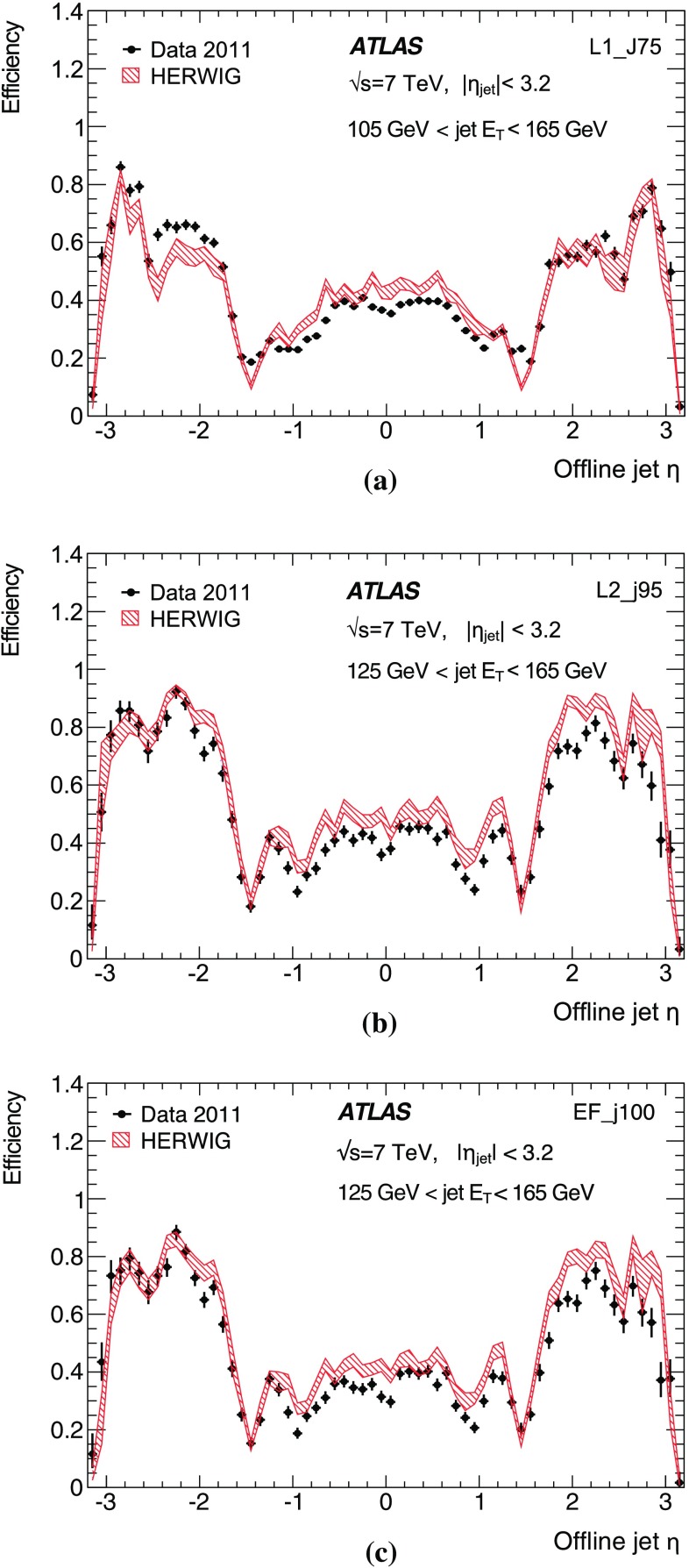
The single inclusive jet trigger efficiency integrated over the $E_\mathrm{T}$ of the rising edge of the trigger, as a function of $\eta$ for the triggers: **a** L1_J75; **b** L2_j95; **c** EF_j100. The data are shown as the *solid points* with *error bars* with the simulated sample shown as a shaded band. Statistical uncertainties only are shown.

To quantify the behaviour of the trigger efficiency in the vicinity of the rising edge as a function of pseudorapidity, it is informative to study the efficiency, differential in $\eta$, but integrated over the $E_\mathrm{T}$ interval defined by the 1 and 99 % efficiency points of the sample as a whole. Figure 18 shows this integrated single inclusive jet trigger efficiency, as a function of $\eta$ for the trigger chain consisting of thresholds of 75, 95 and 100 GeV at L1, L2 and the EF, respectively. A lower efficiency is seen near $|\eta | = 1.5$, corresponding to the crack region between the barrel and endcap calorimeters where the measured energy in the calorimeter will be lower. These variations are related to the detector geometry and detector conditions, and are very strongly correlated with the offsets observed in the previous section, where for instance, the larger (negative) offset seen in the barrel results in fewer jets passing the trigger threshold. Related to what was seen in Sect. 5.3, a small asymmetry is observed between the positive and negative barrel regions.

#### The multi-jet trigger efficiency

A multi-jet trigger requires that *N* jets in the event pass certain $E_\mathrm{T}$ thresholds. For the triggers considered in this study, all jets must be reconstructed in the central part of the calorimeter ($|\eta |<2.8$). When searching for final states with large jet multiplicities in the high energy environment of the LHC, the requirement of several jets means that a multi-jet trigger is more likely to remain unprescaled than its single jet counterpart.

However, the principal disadvantage of a multi-jet trigger is an overall loss in efficiency due to limitations in both transverse energy and angular resolution at L1 and L2. This loss in efficiency is compounded by the jet multiplicity requirement in the trigger, but is less significant for offline jets when they are geometrically isolated. The primary reasons for these inefficiencies in the trigger are the use of the square sliding window and reduced granularity at L1, and the limited RoI size used for the reconstruction of jets at L2.

Multi-jet triggers have been used in signal selection and multi-jet background estimation in searches for the Higgs boson, supersymmetry, and other, beyond-the-SM, processes [39–41]. During 2011, multi-jet triggers requiring between three and six jets were available, with $E_\mathrm{T}$ thresholds at the EF ranging from 30 to 100 GeV.

For a multi-jet trigger efficiency, when requiring a signature containing *N* jets with a single common threshold, the efficiency will essentially be determined by the efficiency for triggering on the *N*-th leading jet in $E_\mathrm{T}$. For simplicity, only multi-jet triggers with a single common threshold are considered here. For multi-jet efficiencies, it is therefore more useful to determine the *event level efficiency*, determined as a function of the $E_\mathrm{T}$ of this *N*-th jet.

**Fig. 19:**
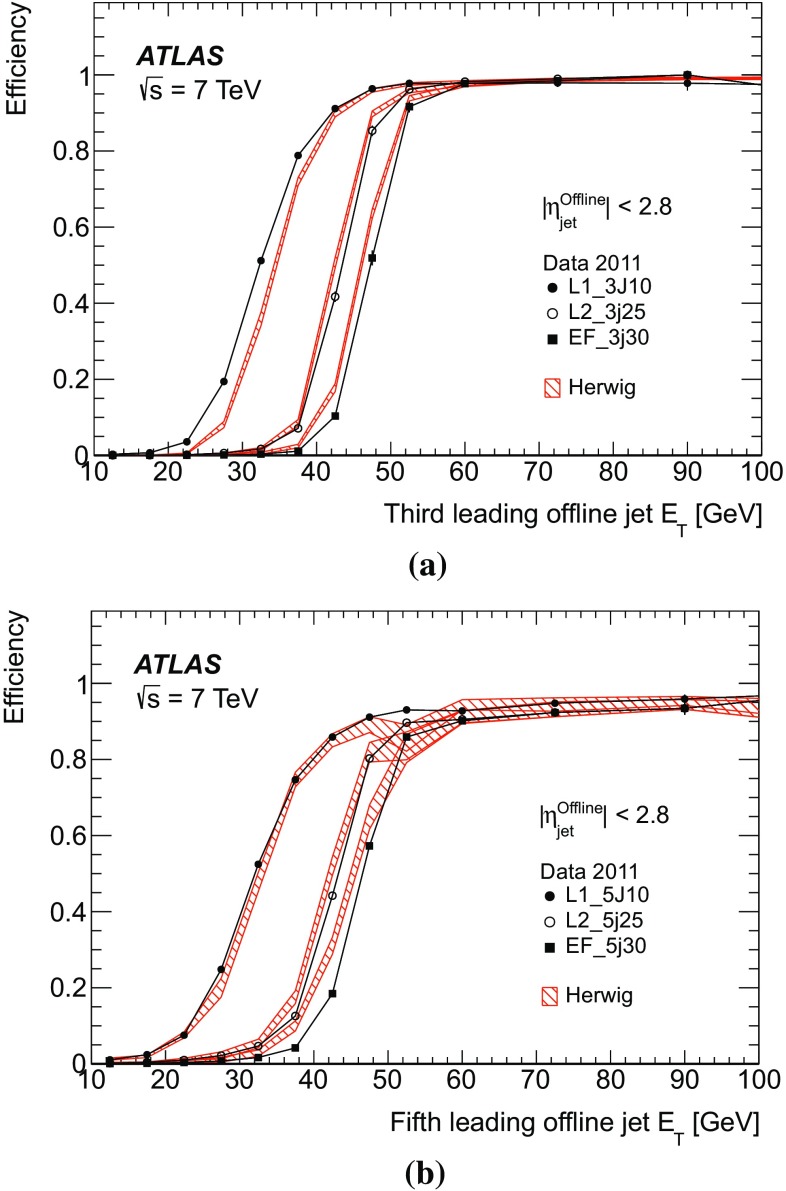
The efficiency for the three-jet and five-jet chains with a 30 GeV threshold at the EF, as a function of: **a** the *third* jet $E_\mathrm{T}$ for the three jet chains; and **b** as a function of the *fifth* jet $E_\mathrm{T}$ for the five jet trigger chains. Shown are the absolute trigger efficiencies: the L2 efficiency also includes that for L1 and the EF efficiency includes that from both L1 and L2. Statistical uncertainties only are shown: the data are shown as the solid points with error bars, and the Herwigsimulated sample as the hatched band.

The characteristics of multi-jet triggers are illustrated in Fig. 19, which shows the efficiency for the lowest $E_\mathrm{T}$, three jet, and five jet trigger chains. The reference triggers were chosen to have a combination of a lower jet multiplicity and a lower $E_\mathrm{T}$ requirement, compared to the trigger being studied, so that they are fully efficient over the rising edge of the trigger being studied. For the three jet trigger chain, the reference trigger at L1 required the event to pass either the random seeded, 10, 15, or 20 GeV EF triggers, operating beyond their respective plateaux. For the three jet chain at L2 and EF, the L1 threshold at 10 GeV was required, with pass-through at L2 and EF. For the four jet and five jet trigger chains the requirement of three EF jets above 30 GeV was used as the reference trigger. In contrast to the single inclusive jet trigger analysis, no jet matching is applied from one level to the next, and no jet isolation is imposed unless specifically stated. When the jet multiplicity requirement is increased from three to five, the plateau efficiency decreases and the uncertainties on the simulated sample increase, due to the smaller Monte Carlo sample size.

In order to allow a very approximate quantitative comparison of the efficiency for a selection of jet triggers with different multiplicities, a fit to the efficiency distributions for four multi-jet trigger chains has been performed and the relevant parameters extracted. A sigmoid function was chosen to parameterise the efficiency,

10 $$\begin{aligned} \varepsilon (E_\mathrm{T}) = c_3 + (c_0 - c_3) \left[ 1+\exp \left( -\frac{{E_\mathrm{T}} - c_1}{c_2}\right) \right] ^{-1} \end{aligned}$$

where $c_0$ is the plateau efficiency in percent, $c_1$ is the *midpoint* of the rising edge, in GeV, $c_2$ – also in GeV – is related to the width or *sharpness* of the rising edge, and $c_3$ is the residual efficiency in the region before the trigger begins to turn on.

**Fig. 20:**
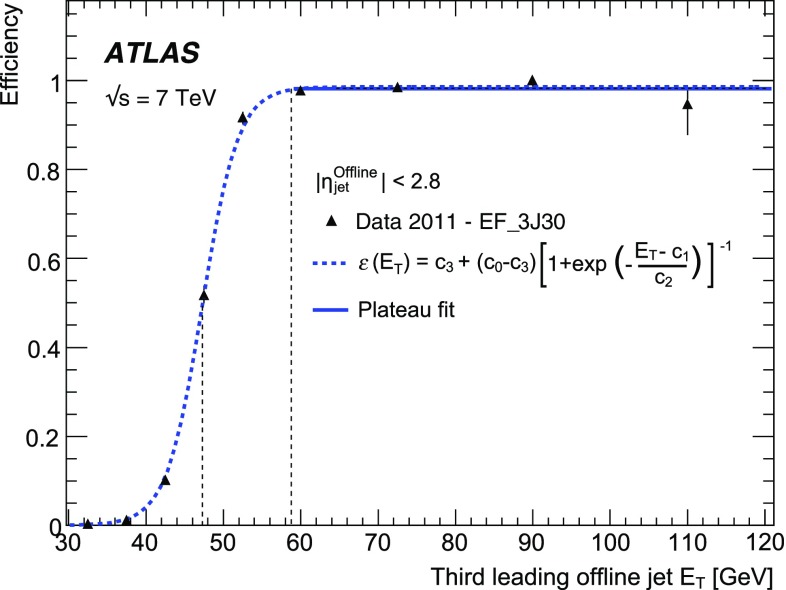
The sigmoid fit to the rising edge of the efficiency and the fit purely to the plateau region for the EF_3j30 trigger, without any jet isolation requirement. The parameter $c_0$ represents the plateau efficiency, $c_1$ represents the midpoint of the rising edge, $c_2$ is related to the sharpness of the rising edge, and $c_3$ is the efficiency prior to the rising edge. The *horizontal solid blue line* indicates the plateau efficiency. The *vertical dashed lines* indicate the rising edge midpoint ($c_1$), and the start of the plateau ($c_1 + 5c_2$)

The plateau efficiency was also determined using the parameters from the sigmoid fit and, additionally, fitting a constant to the region $E_\mathrm{T}$ $> c_1 + 5c_2$, corresponding approximately to the region where the efficiency is above 99 % of the ultimate value. This provides an alternative determination of the plateau efficiency. Figure 20 shows example fits for the EF_3j30 chain.

Table 3 displays the plateau efficiency and parameters describing the efficiency at each trigger level, for the lowest $E_\mathrm{T}$ single, three, four and five jet trigger chains. This table highlights the loss in plateau efficiency with increasing jet multiplicity, the consistency of the rising edge midpoint between different jet multiplicities, and the general reduction of sharpness of the rising edge for higher multiplicities.

**Table 3:** The plateau efficiency from the linear fit, and the midpoint $E_\mathrm{T}$ and sharpness of the rising edge from the sigmoid fit, for the single, three, four, and five jet trigger chains, each with an EF threshold of 30 GeV and without offline jet isolation. The plateau efficiency decreases with increasing jet multiplicity

Trigger	Plateau [%]		Midpoint [GeV]		Sharpness [GeV]	
L1_J10	98.00	± 0.04	30.77	± 0.04	4.10	± 0.03
L2_j25	99.65	± 0.02	43.01	± 0.01	1.94	± 0.01
EF_j30	99.75	± 0.02	47.09	± 0.02	1.94	± 0.01
L1_3J10	97.3	$^{+~ 0.3}_{-~ 0.4}$	32.0	± 0.1	2.92	± 0.03
L2_3j25	98.6	$^{+~ 0.4}_{-~ 0.5}$	43.6	± 0.1	2.78	± 0.06
EF_3j30	98.1	$^{+~ 0.5}_{-~ 0.6}$	47.3	± 0.1	2.30	± 0.07
L1_4J10	95.2	± 0.1	30.20	± 0.02	3.93	± 0.02
L2_4j25	95.0	± 0.1	41.98	± 0.02	3.06	± 0.02
EF_4j30	94.7	± 0.1	46.30	± 0.02	2.74	± 0.02
L1_5J10	93.4	± 0.3	31.50	± 0.04	3.71	± 0.02
L2_5j25	91.3	± 0.5	42.84	± 0.06	2.47	± 0.04
EF_5j30	91.1	± 0.5	46.56	± 0.07	3.17	± 0.04

The plateau efficiency decreases with increasing jet multiplicity because of the limitations of accurately reconstructing jets which are not well separated and discriminating between them at L1 and L2.

**Fig. 21:**
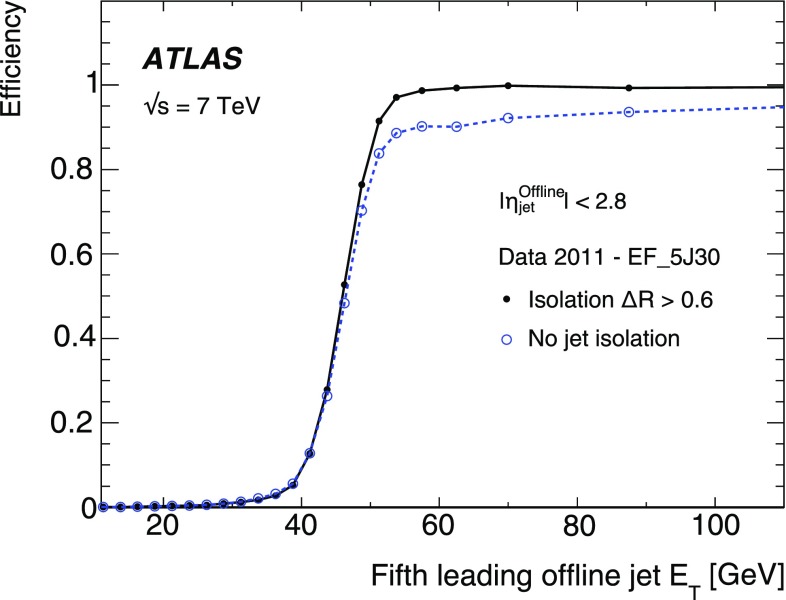
The efficiency as a function of the fifth jet $E_\mathrm{T}$ for the five-jet EF trigger, where no jet isolation is required (*dashed line*) and where jet isolation is required (*solid line*). Shown are the absolute trigger efficiency, including both the L1 and L2 efficiencies. Statistical uncertainties only are shown.

Figure 21 shows the absolute efficiency including the contributions from L1 and L2, for the five jet trigger chain as a function of the fifth jet $E_\mathrm{T}$. The solid curve in the figure shows the same efficiency for events where the leading offline jets are separated by a distance $\Delta R > 0.6$ with respect to the corresponding closest jets. In this case, the isolation requirement is applied only to the four leading jets – there is no requirement on the isolation of the fifth leading jet. The difference observed with these different isolation requirements clearly illustrates that this loss in efficiency is primarily due to issues in the reconstruction of poorly separated jets in the L1 and L2 triggers. This effect is shown quantitatively, for the three multi-jet trigger chains studied, in Table 4.

**Table 4:** The plateau efficiency from the linear fit, and the midpoint $E_\mathrm{T}$ and sharpness of the rising edge from the sigmoid fit, for the three, four and five jet trigger chains, with an EF threshold of 30 GeV and with jet isolation applied between the *N* leading offline jets. By imposing jet isolation the loss in plateau efficiency at the EF is recovered

Trigger	Plateau [%]		Midpoint [GeV]		Sharpness [GeV]	
L1_3J10	99.3	± 0.1	31.94	± 0.04	2.84	± 0.02
L2_3j25	99.7	± 0.2	43.29	± 0.08	2.26	± 0.04
EF_3j30	99.5	± 0.2	46.96	± 0.09	2.09	± 0.05
L1_4J10	99.60	± 0.02	30.21	± 0.02	3.89	± 0.01
L2_4j25	99.64	± 0.03	42.15	± 0.01	2.47	± 0.01
EF_4j30	99.71	± 0.03	46.08	± 0.01	2.37	± 0.01
L1_5J10	99.4	± 0.1	31.32	± 0.02	3.61	± 0.01
L2_5j25	99.4	± 0.1	42.66	± 0.03	2.79	± 0.02
EF_5j30	99.5	± 0.1	45.98	± 0.04	2.66	± 0.02

## Jet identification for *pp* collisions performed by specialised jet triggers

To further exploit the *pp* data, jet triggers designed to reconstruct specific physics signatures are used in the ATLAS trigger. In 2011 these included $H_\mathrm{T}$ triggers, cutting on the scalar transverse energy sum of all jets, and triggers identifying jets with large radii discussed below.

### $H_\mathrm{T}$ triggers

In many searches for physics beyond-the-standard model (BSM), including Supersymmetry (SUSY) and other exotic physics signatures, the hard process gives rise to a final state containing energetic jets and a large missing transverse momentum. The selection adopted to discriminate the signal process from the background in such searches typically includes requirements on the $E_\mathrm{T}$ and the scalar sum of transverse momenta of all selected physics objects. Missing transverse momentum triggers [42] can be used in such searches; however, an alternative approach is the use of $H_\mathrm{T}$ triggers which reconstruct the total scalar sum of jet transverse energy ($H_\mathrm{T}$) in an event at the EF. The $H_\mathrm{T}$ triggers are useful for physics analyses that study, or search for, events with large overall $E_\mathrm{T}$ in the final state. In this case, the requirement of large $H_\mathrm{T}$ can help to control the trigger rate without requiring a very energetic leading jet, although a leading jet with some $E_\mathrm{T}$ may still be required to seed the reconstruction. Because the resolution of the missing transverse momentum reconstruction in the trigger is poor for small values, using an $H_\mathrm{T}$ based trigger is a realistic alternative to using a missing transverse momentum trigger for final states where the missing transverse momentum is small.

The $H_\mathrm{T}$ triggers were introduced to the trigger menu in 2011, the primary motivation being the selection of events for searches for SUSY in events with no leptons [43]. Single, and multi-jet $H_\mathrm{T}$ triggers exist, where the single jet $H_\mathrm{T}$ triggers are seeded by a standard single inclusive trigger and the multi-jet $H_\mathrm{T}$ triggers are seeded by a standard multi-jet trigger. These seeding triggers are required since the calculation of $H_\mathrm{T}$ without such a seeding trigger would require full jet finding in all events, which would be computationally prohibitive in the trigger.

To illustrate the $H_\mathrm{T}$ trigger performance a single $H_\mathrm{T}$ trigger has been selected, requiring a leading energetic trigger jet, with $E_\mathrm{T}$ $> 100$ GeV, and total $H_\mathrm{T}$ $>400$ GeV at the EF. The L1 and L2 stages for the $H_\mathrm{T}$ triggers are identical to those of the single and multi-jet chains discussed in Sect. 5.4. Thus the efficiencies shown in Fig. 15 for the L1_J75 and L2_j95 triggers are relevant for the specific $H_\mathrm{T}$ trigger discussed here.

The quantity $H_\mathrm{T}$ in the trigger is calculated from all EF jets with an $E_\mathrm{T}$ above a specified threshold, and within $|\eta |< 3.2$, to exclude jets reconstructed in the less well understood forward region. There are thus two key factors that affect the performance of an $H_\mathrm{T}$ trigger; the leading jet $E_\mathrm{T}$ requirement and the jet $E_\mathrm{T}$ threshold for summing the $H_\mathrm{T}$. These factors are investigated and presented below.

**Fig. 22:**
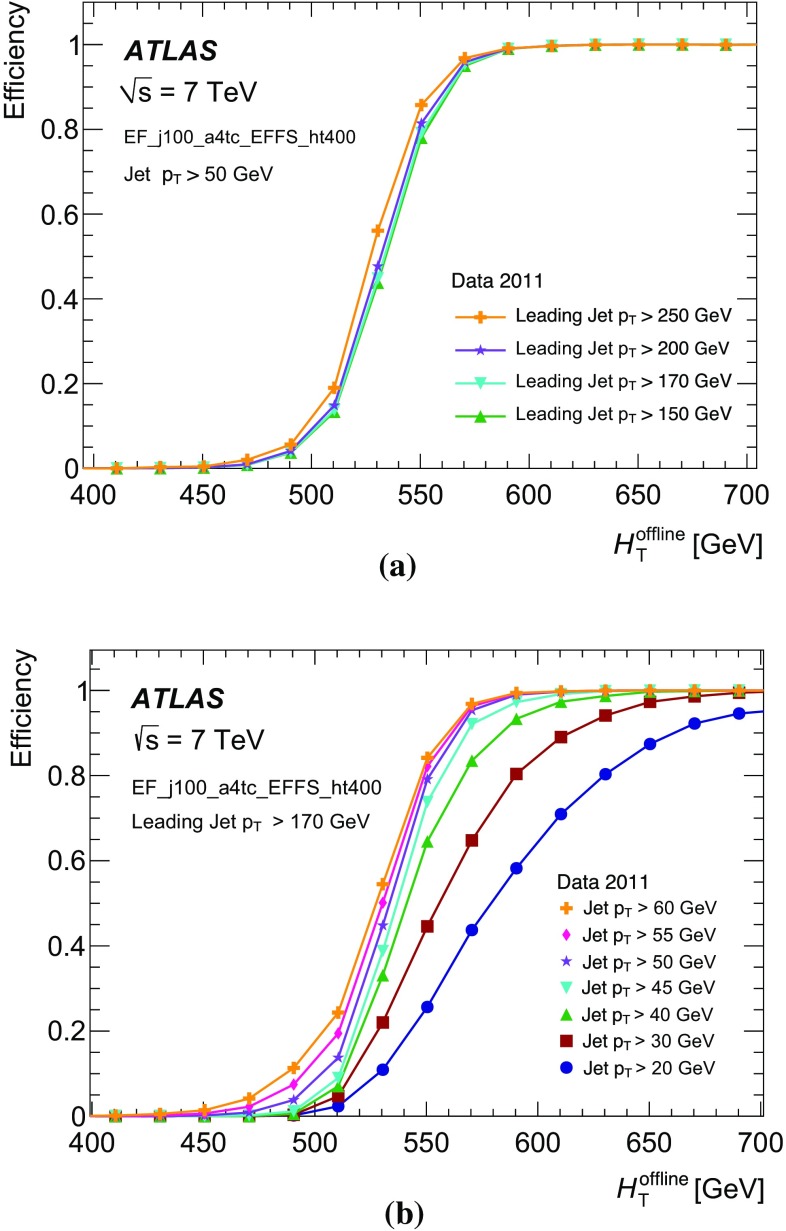
The efficiency for the trigger requiring both $H_\mathrm{T}$ $>400$ GeV and a leading jet with $E_\mathrm{T}> 100$ GeV at the trigger level shown as a function of offline $H_\mathrm{T}$ for: **a** various leading offline jet $p_{\text {T}}$ selections, where the offline $H_\mathrm{T}$ is calculated from offline jets with $p_{\text {T}} > 50$ GeV; **b** various offline jet $p_{\text {T}}$ selections for the definition of offline $H_\mathrm{T}$, where the leading offline jet $p_{\text {T}}$ selection is 170 GeV

The efficiency with respect to the offline $H_\mathrm{T}$, for the $H_\mathrm{T}$ trigger requiring the leading jet $E_\mathrm{T}$ at the trigger level to be greater than 100 GeV, and the total trigger $H_\mathrm{T}$ to be larger than 400 GeV, is shown in Fig. 22 for various choices of the offline $H_\mathrm{T}$ definition. Figure 22a shows the effect of changing the leading offline jet $p_{\text {T}}$ requirement in the definition of the offline $H_\mathrm{T}$, formed in this case from all offline jets with $p_{\text {T}}$ greater than 50 GeV. With the specified trigger thresholds, the efficiency is seen to be relatively insensitive to changes in the choice of the leading offline jet $p_{\text {T}}$ selection within the range shown and remains at maximum efficiency for offline $H_\mathrm{T}$ $>600$ GeV for all illustrated leading offline jet selections.

Figure 22b shows the effect of changing the $p_{\text {T}}$ threshold for all offline jets used in the calculation of the offline $H_\mathrm{T}$. In this case the leading offline jet $p_{\text {T}}$ threshold used in the offline definition is 170 GeV. The trigger is seen to maintain full efficiency only for offline $H_\mathrm{T}$ $>600$ GeV for those definitions where the selected offline jets are required to have $p_{\text {T}}$ greater than approximately 50 GeV. Definitions where the offline jet $p_{\text {T}}$ selection is reduced to 40 GeV, are seen to incur no corresponding loss in efficiency only when the offline $H_\mathrm{T}$ is greater than 650 GeV.

In conclusion the performance of the $H_\mathrm{T}$ trigger when seeded by a single high $E_\mathrm{T}$ jet is more sensitive to the choice of jet $E_\mathrm{T}$ threshold for calculating $H_\mathrm{T}$ than the choice of leading jet $E_\mathrm{T}$. This is because the choice of the leading jet is primarily a selection on the events from which the jets used in the $H_\mathrm{T}$ calculation are taken and does not significantly affect the value of $H_\mathrm{T}$ in that event, whereas the selection of the overall jet threshold used in the definition of $H_\mathrm{T}$ will change the calculated value of $H_\mathrm{T}$. Therefore as long as the leading jet threshold is chosen such that the single inclusive trigger is maximally efficient for the leading jet threshold used in the offline definition of $H_\mathrm{T}$ then the $H_\mathrm{T}$ trigger will be maximally efficient given a suitable choice of offline threshold for the remaining jets.

### Large-$R$ jet triggers

Physics analyses studying the properties of, or searching for, new or heavy particles decaying into boosted hadronic final states, may include kinematic regions where the decay products are more collinear. Such events may not be triggered by standard ($R=0.4$) multi-jet triggers, if the jets are too close to one another to be resolved. In such situations, large-$R$ jet triggers are useful. For example, the decay products of a top quark produced with an $E_\mathrm{T}$ above 300 GeV might be contained within a single jet with about twice the radius of a standard jet. One such ATLAS study involving boosted top quarks where the hadronic decay products can not be resolved as individual jets, is the search for new heavy resonances decaying into $t\bar{t}$ pairs [44]. Large-$R$ jet triggers are useful in such searches [45, 46] and also jet substructure studies [47].

The large-*R* jet triggers at the EF are essentially the same as the standard jet triggers discussed earlier; they are seeded by L1 and L2 triggers and use the same reconstruction algorithms as the standard jets but with a larger jet radius, namely $R=1.0$, in contrast to the $R=0.4$ of the standard jet triggers.

The EF_j240_a10tc trigger, designed to target such final states as described above, is described in this section. This was the lowest $E_\mathrm{T}$ unprescaled large-$R$ jet trigger for the 2011 running period.

**Fig. 23:**
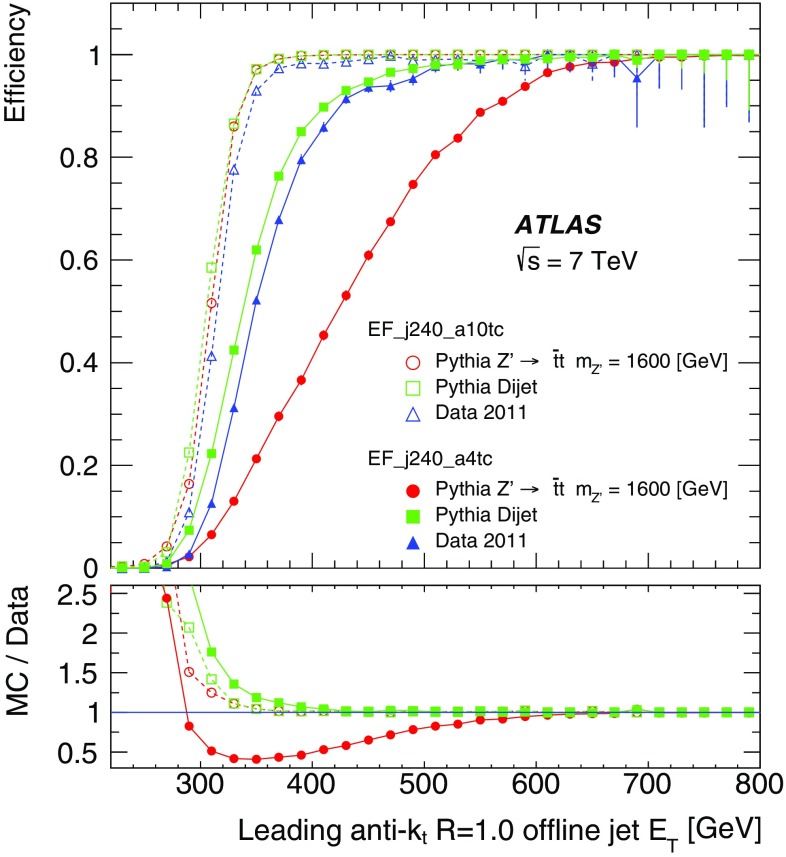
The efficiency of the large-$R$ jet trigger, EF_j240_a10tc (*open markers*), compared to the standard EF_j240_a4tc trigger (*filled markers*), both with respect to offline $R=1.0$ jets, for both data and simulation

Two Pythia [34] Monte Carlo samples were selected to study such scenarios, both containing a large number of high $E_\mathrm{T}$ jets. The first sample, labelled Pythia Dijet, contains predominantly light quarks and gluons from the hard process, and the second sample, labelled Pythia $Z^{\prime }\rightarrow t\overline{t}$, models the production of a BSM heavy $Z^\prime$ gauge boson, decaying to a top anti-top quark pair. For the specific Pythia Dijet sample chosen, the $E_\mathrm{T}$ of the leading jet in each event lies in the range 300–600 GeV.

In Fig. 23 the trigger efficiency of the large-$R$ jet trigger is compared with that of the standard jet trigger with the same energy threshold. The single inclusive jet trigger efficiency has a sharper rising edge when applied to the Pythia Dijet sample, in which the jets have most of their energy occupying a narrow cone, than when applied to the Pythia $Z^\prime \rightarrow t\overline{t}$ sample, in which the jets are the result of the decay of heavy, boosted objects. The large-*R* jet trigger has both a sharper rising edge than the standard jet trigger, and similar behaviour for all samples. This suggests that the large-$R$ trigger is less sensitive than the standard jet trigger to the quark or gluon nature of the jets studied.

**Fig. 24:**
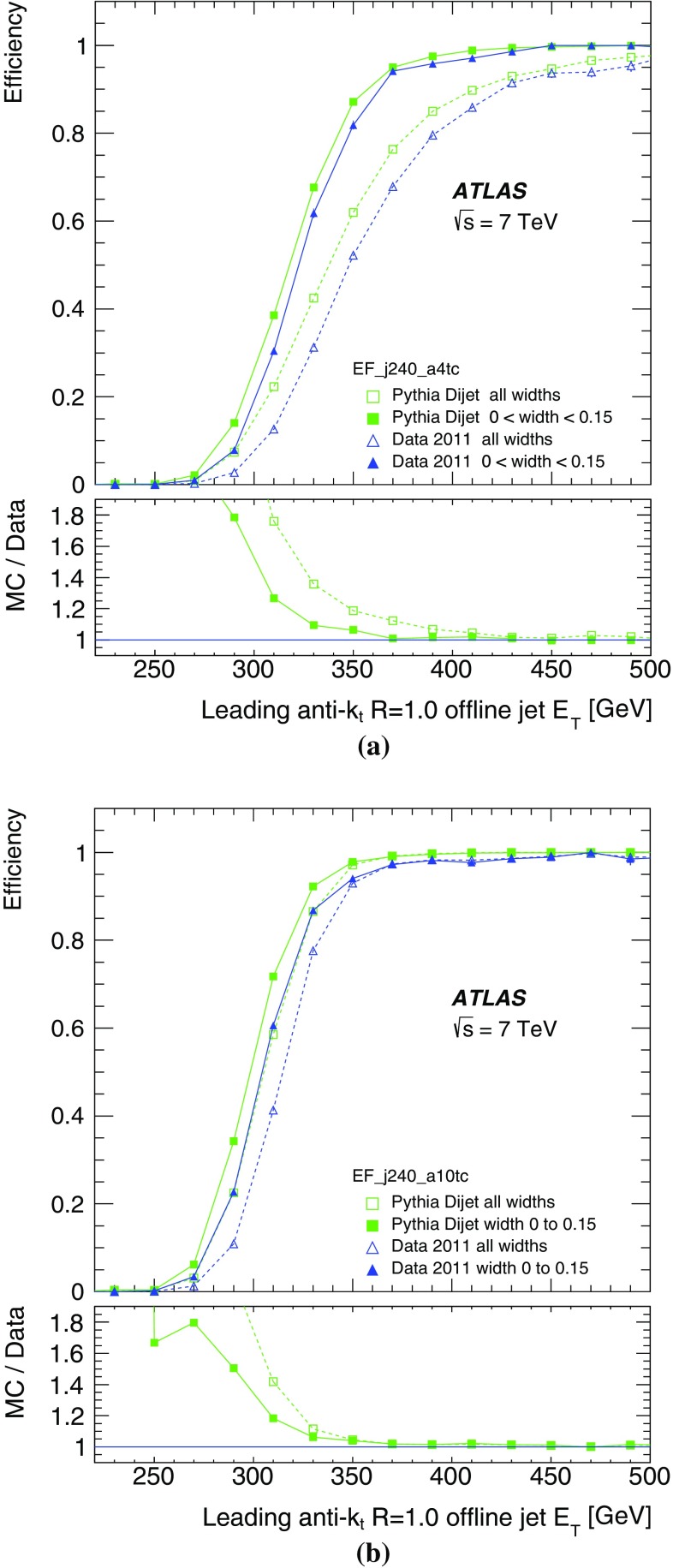
The effect of jet width on efficiency, for data and Monte Carlo, with respect to offline $R=1.0$ jets: **a** when the standard jet trigger, EF_j240_a4tc, is used; and **b** where the large-$R$ jet trigger, EF_j240_a10tc, is used. Inclusive jet widths (*open markers*) and widths restricted to $w<0.15$ (*filled markers*) are shown

In Fig. 24 the sensitivity of the jet trigger to differences between quark-initiated and gluon-initiated jets is explored further. Since gluon-initiated jets are typically wider than quark-initiated jets, selecting narrower jets with a width [46] $w<0.15$, would slightly favour quark-initiated jets from the dijet sample. A slightly sharper rising edge than seen in the inclusive sample is observed. The differences between the efficiencies for the samples with different widths are more distinct for the standard jet trigger, seen in Fig. 24a, than the large-$R$ jet trigger, as shown in Fig. 24b. The performance of the jet trigger in the simulation is seen to be broadly in agreement with data for the large-$R$ jet trigger where the larger jet width selection exhibits a sharper rising edge. The rising edge is considerably less sharp for the standard jet trigger and shows significantly more variability with the properties of the sample. The large-$R$ jet trigger is therefore more robust to changes of the jet width, which is a measure of jet substructure and radiation profiles.

The single inclusive jet triggers with $R=0.4$ have a sharply rising edge for jets with a narrow energy core, but the performance is reduced for jets with wider, or multi-pronged energy distributions. Jet triggers with large-$R$ not only improve the performance for jets with wide energy distributions but also improve the performance for jets with a narrow energy core although at the cost of greatly increasing both the sensitivity of the trigger to pile-up, and the trigger rate.

## Jet identification for heavy ion collisions

Heavy ion (HI) collisions differ significantly from *pp* collisions: the intrinsic geometry of nuclear collisions results in large variations of both the track multiplicity and the energy density. The data collected in 2011 for the ATLAS HI programme included collisions of lead nuclei with a nucleon–nucleon centre-of-mass energy of 2.76 TeV. Dedicated HI triggers are required for the very different environment of HI collisions. Jets produced in HI collisions can be used as direct probes of the resultant evanescent, hot, dense medium, and as such represent a very important tool for physics studies [48–50]. Studies of such jets at ATLAS in 2010 led to the first direct observation of a dijet asymmetry, or *jet quenching*, in Pb+Pb collisions [51].

The study of a full range of observables which characterise the hot and dense medium formed in HI collisions is possible with specific HI triggers. In addition to global measurements of quantities such as particle multiplicity and collective flow, electroweak gauge boson production, heavy-quarkonia suppression, and the modification of jets passing through the dense medium are accessible with ATLAS data [48–50].

**Fig. 25:**
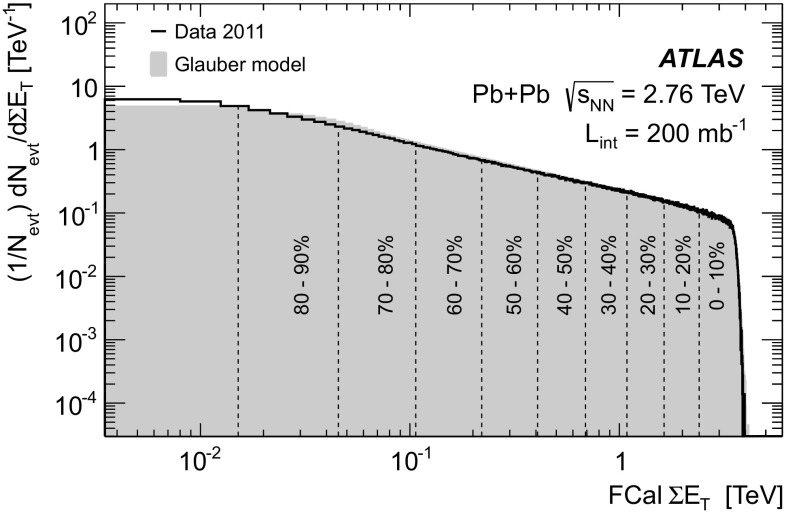
The distribution of the transverse energy deposited in forward calorimeter, FCal $\Sigma E_{\mathrm {T}}$, divided into 10 % centrality bins [53]

The dominant issue for jet measurements in the HI environment is the presence of a large amount of additional energy coming from the underlying event (UE), additional interactions originating from the same Pb+Pb collision. The properties of this energy depend on the impact parameter, or minimum transverse distance between the two colliding nuclei. A direct measurement of the impact parameter is not possible and so the *centrality* of the collision, defined as the $E_\mathrm{T}$ deposited in forward calorimeter, FCal $\Sigma E_{\mathrm {T}}$, is used to characterise the UE [52]. The distribution of FCal $\Sigma E_{\mathrm {T}}$ is shown in Fig. 25 and is divided into bins according to percentiles of the total Pb+Pb cross section.

The 0–10 % centrality bin represents the most central collisions, with the smallest impact parameter and the largest UE contributions. The 60–80 % centrality bin represents the most peripheral collisions used in this study, corresponding to the largest impact parameters and the smallest UE contributions. Jet reconstruction in HI events typically corrects for the contributions of the underlying event.

During the first LHC Pb+Pb run, in 2010, all events identified by the minimum bias (MB) and forward detector triggers [3, 13] were recorded and used for the HI studies. However, in 2011 the instantaneous luminosity substantially increased, such that the peak rate of MB L1 triggers exceeded 6 kHz. Therefore to maintain efficiency for events containing high $p_{\text {T}}$ jets, MB events identified at L1 were passed through L2 to the EF where specialised HI jet triggers were used to select events.

The L1 MB triggers based on the total summed transverse energy in the calorimeter were used as the L1 seeds for HLT triggers which reconstruct high $p_{\text {T}}$ electrons, muons and jets. Events were transferred directly to the EF if the total transverse energy deposited in the calorimeters exceeded 10 GeV. A detailed description of the performance of various MB triggers in HI collisions can be found elsewhere [54].

Jets at the EF were reconstructed across the entire calorimeter (including the forward region) using the anti-$k_{t}$ algorithm with radius parameter $R=0.2$ from projective towers of size $\Delta \eta \times \Delta \phi = 0.1 \times 0.1$ formed from the summation of calorimeter cell energies. The small $R=0.2$ radius parameter was chosen in order to be less sensitive to fluctuations in the underlying event, the contribution of which is estimated and subtracted event-by-event from each jet at the calorimeter cell level after the jet finding. The subtraction is performed separately in each 0.1 $\eta$ region and in each calorimeter sampling layer (*i*). The background subtracted cell energies ($E_{{\mathrm T}j}^{\mathrm {sub}}$) are calculated according to:

11 $$\begin{aligned} E_{{\mathrm T}j}^{\mathrm {sub}} = E_{{\mathrm T}j} - A_{j}\rho _{i}(\eta _{j}) \end{aligned}$$

where $E_{\mathrm {T} {j}}$ is the measured cell $E_\mathrm{T}$, $\rho _{ {i}}$ is the average $E_\mathrm{T}$ density in a given $\eta$ region and layer, $A_{ {j}}$ is the cell area and *j* runs over all calorimeter cells. Cells within jet candidates are excluded from the estimate of average UE energy density $\rho$ to reduce biases. Jet candidates are required to have at least one tower with $E_{\mathrm {T}}> 3$ GeV and a ratio of maximum tower $E_\mathrm{T}$ to average tower $E_\mathrm{T}$ greater than four.

### Performance of the heavy ion triggers

The performance of the HI jet trigger is evaluated here using the Pb+Pb collision data recorded near the end of the 2011 data taking period which corresponds to an integrated luminosity of 140 $\mathrm {\mu b}^{-1}$.

**Fig. 26:**
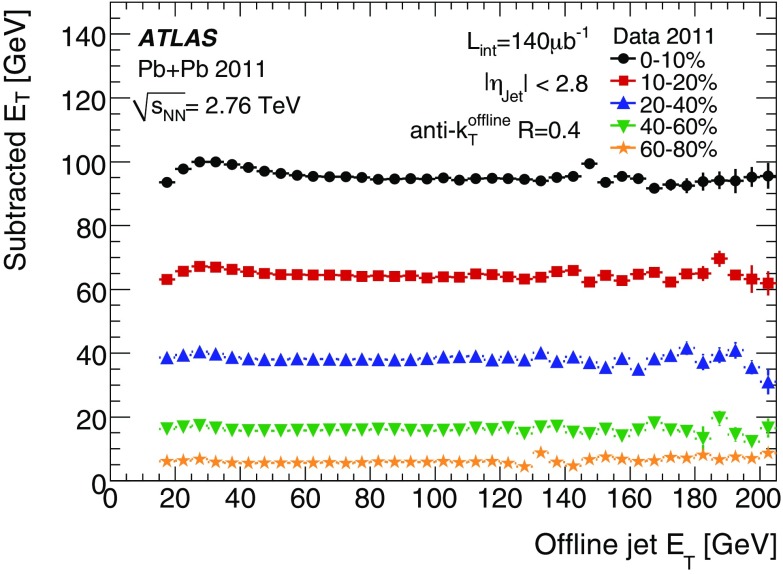
The mean transverse energy subtracted from offline jets with $R=0.4$, as a function of jet $E_\mathrm{T}$ for five centrality bins for the heavy ion data

Offline jets are reconstructed from calorimeter towers, using the anti-$k_{t}$ algorithm, with $R=0.2, 0.3, 0.4$ and 0.5 in the region $|\eta |<2.8$. Unlike the trigger jets, the offline energy is corrected for the lower hadronic response of the non-compensating ATLAS calorimeters, using calibration constants obtained from Monte Carlo simulation using Pythia [34] embedded in the Hijing [55] event generator. In the offline reconstruction for HI events, an event-by-event correction for elliptic flow, a long-range correlation originating from the azimuthal momentum anisotropy of particle emission, and a second iteration of the anti-$k_{t}$ algorithm are made to improve the performance of the UE estimation [56]. Figure 26 shows the mean estimated UE contribution to be subtracted from an $R=0.4$ offline jet as a function of the jet $E_\mathrm{T}$ for different centrality bins. A small variation of the estimated UE contribution at the lowest $E_\mathrm{T}$ is corrected for in the offline analysis. More details regarding offline jet reconstruction in HI events can be found elsewhere [57].

In HI events it is not uncommon for fluctuations in the UE to create regions with high $E_\mathrm{T}$ in the calorimeter that do not originate from hard-scattering processes but which can nevertheless be reconstructed as jets. To remove these jets it is required that offline calorimeter jets are matched to a single electromagnetic cluster with $E_\mathrm{T}$ $>7$ GeV or to at least one *track jet* - a jet formed using tracks from charged particles rather than calorimeter energy deposits. In this case, track jets are reconstructed using the anti-$k_{t}$ algorithm with $R=0.4$ applied to tracks with $p_{\text {T}} > 4$ GeV. This procedure is referred to as the fake-jet rejection (FJR). Except where noted, the offline jet studies in this section include FJR.

The efficiency of a trigger is defined as the per jet probability to satisfy the trigger requirements as a function of offline jet $E_\mathrm{T}$. Only offline jets matching trigger jets within $\Delta R< 0.4$ contribute to the efficiency. The efficiency of the triggers with thresholds at 15, 20 and 25 GeV, respectively, are studied.

**Fig. 27:**
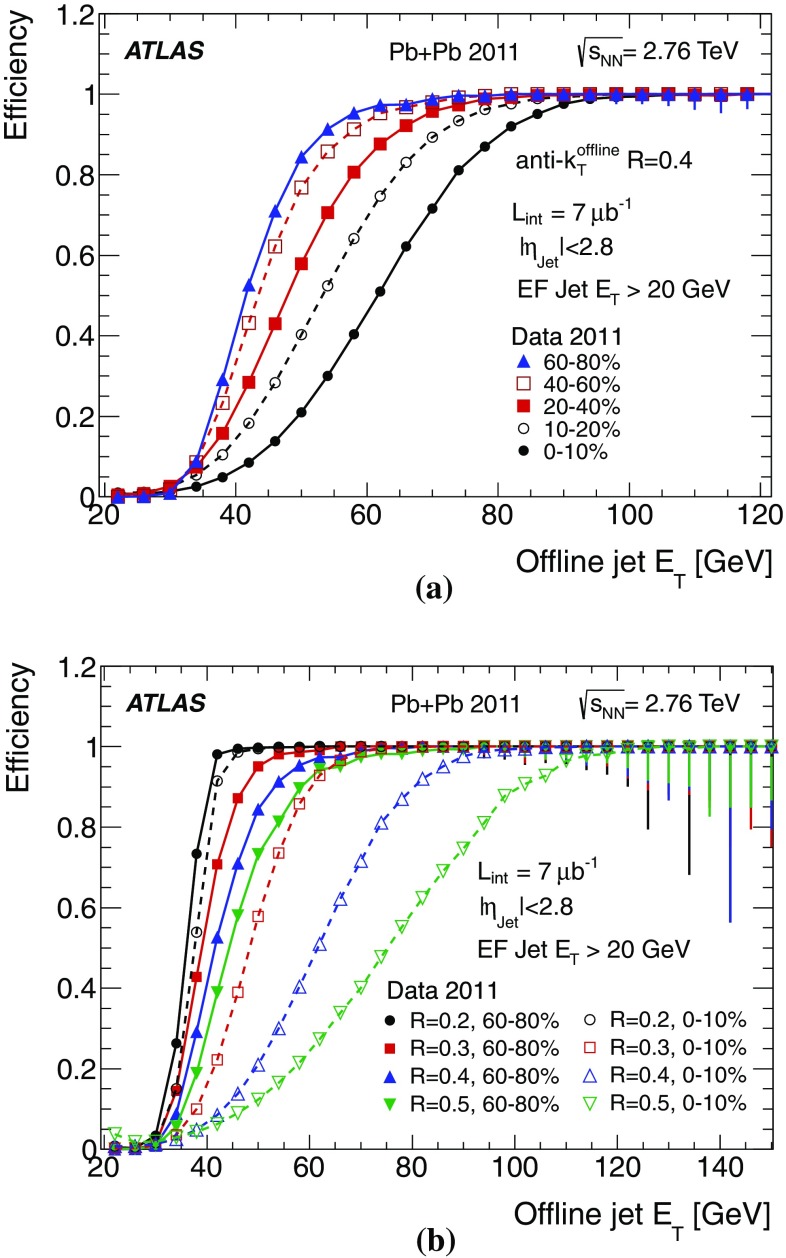
The trigger efficiency versus jet $E_\mathrm{T}$ for heavy ion events: **a** for anti-$k_{t}$ $R=0.4$ offline jets in five centrality bins for a trigger threshold of 20 GeV; **b** in central and peripheral collisions for anti-$k_{t}$ $R=0.2, 0.3, 0.4$ and 0.5 offline jets, also with a trigger threshold of 20 GeV

The performance of the jet reconstruction by the trigger over a range of centralities and radius parameters typically used in HI analyses is illustrated in Fig. 27. Figure 27a shows the trigger efficiency for $R=0.4$ offline jets for the jet trigger with $E_\mathrm{T}$ threshold of 20 GeV. The efficiency decreases with increasing centrality: the 95 % efficiency point of the trigger is reached at 60 GeV in the most peripheral collisions and at 90 GeV in the most central collisions. Full efficiency is reached around 75 GeV and 100 GeV respectively. Figure 27b compares efficiencies for the four radius parameters in the most central and in the most peripheral collisions. Here it is observed that the centrality dependence of the efficiency is more pronounced for larger radius parameters, as the sharpness of the efficiency curves degrades from peripheral to central collisions and from smaller to larger offline jets. This reduction in efficiency for wider jets is expected due to a degradation of energy and angular resolution.

**Fig. 28:**
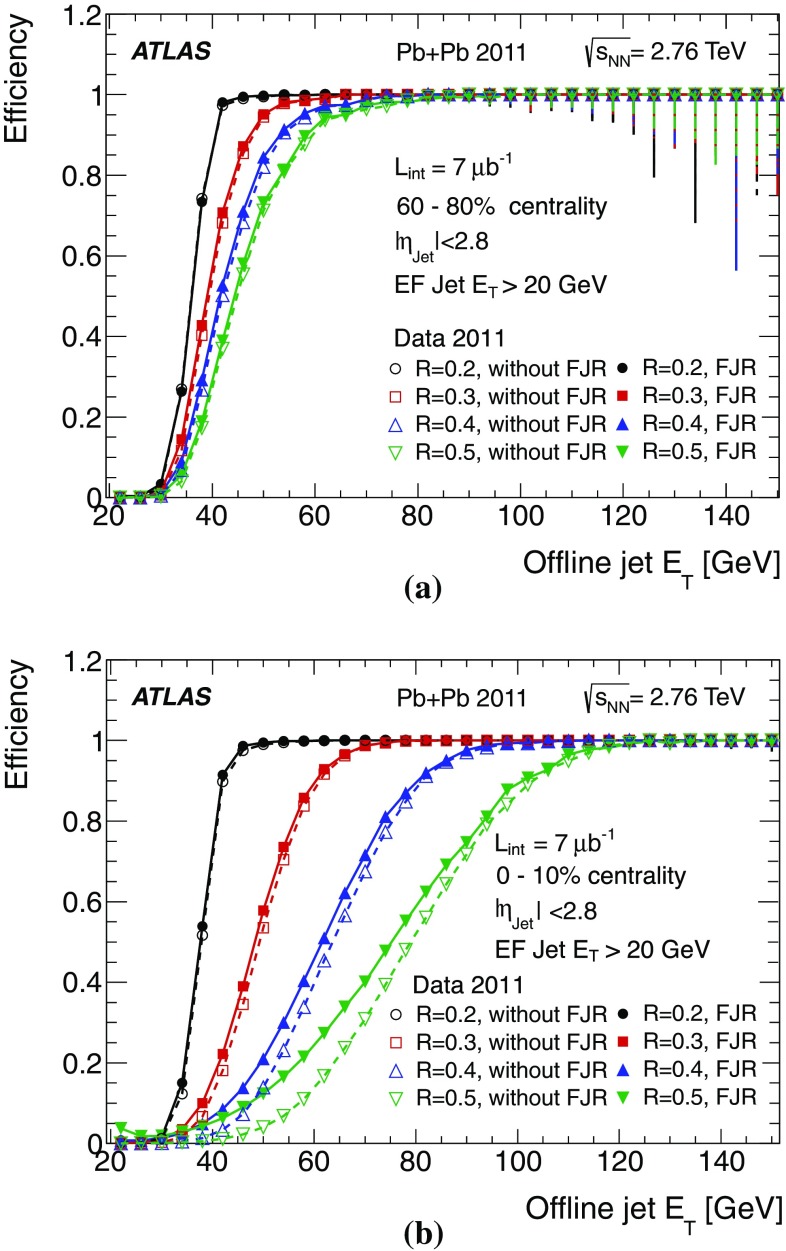
The trigger efficiency for heavy ion events with a requirement of an EF jet with $E_\mathrm{T}>20$ GeV: **a** for peripheral collisions; and **b** for central collisions. Shown are results for offline jets reconstructed with the anti-$k_{t}$ algorithm using $R=0.2$, 0.3, 0.4 and 0.5, both with (*closed points*), and without (*open points*) fake-jet rejection

Figure 28 illustrates the influence of the FJR on the efficiency in (a) peripheral and (b) central collisions. Efficiencies are shown for offline jets for different radius parameters with and without FJR being applied. The efficiency is observed to be slightly lower without FJR. This difference is more marked for central collisions, and increases with the increasing size of the offline jet. This behaviour is caused by two effects: firstly by the increased UE particle multiplicity in central collisions, leading to a greater number of jets being reconstructed from underlying-event fluctuations, and secondly by the increased sensitivity of the trigger jets with larger radius parameter to these UE fluctuations.

**Fig. 29:**
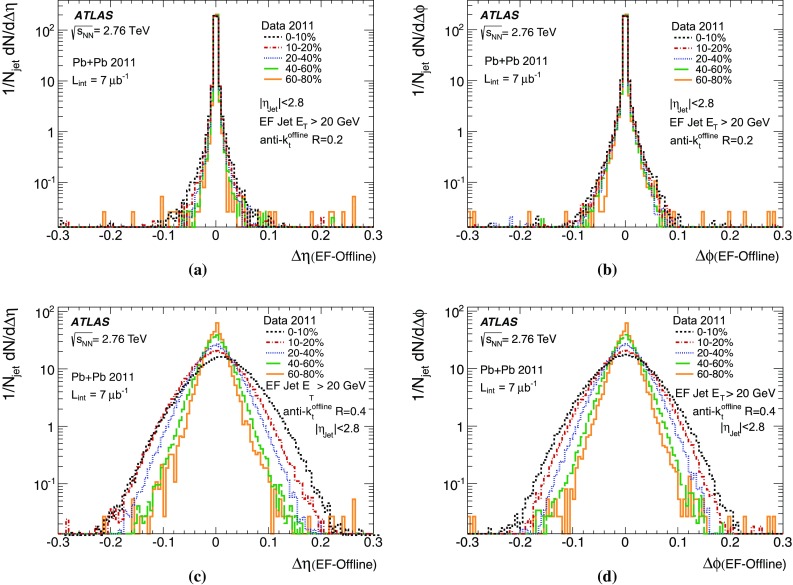
The angular resolution for $R=0.2$ jets with $E_\mathrm{T}>20$ GeV reconstructed in the EF, with respect to $R=0.2$, and $R=0.4$ offline jets, for different centralities in the heavy ion data; the residuals in **a** $\eta$ and **b** $\phi$ between trigger jets and offline $R=0.2$ jets for different centrality bins; the residuals in **c** $\eta$ and **d** $\phi$ between trigger jets and offline $R=0.4$ jets for different centrality bins

The angular resolution of trigger jets with respect to offline jets with $R=0.2$ and $R=0.4$ is shown in Fig. 29 for different centrality intervals. The angular resolution with respect to $R=0.2$ jets shows very weak centrality dependence. However, the angular resolution with respect to $R=0.4$ jets degrades with increasing centrality. This is due to the smearing of the jet direction from the larger underlying-event activity.

The heavy ion programme at the LHC will provide crucial information about the formation of a hot evanescent medium at the highest temperatures and densities ever created in the laboratory. The ATLAS jet trigger algorithm performs well in the HI environment, using the same anti-$k_{t}$ algorithm used for *pp* physics. A small efficiency degradation with increasing centrality is observed, which is less pronounced for smaller radius parameters. The angular resolution is good, but shows some centrality dependence for larger radius parameters.

## Summary

The ATLAS jet trigger has been designed to provide an online reconstruction of jets matching as closely as possible those from the offline reconstruction. For this reason, while the RoI approach is mandatory for reasons of bandwidth limitation at L1 and L2, during Run 1 the jet trigger for the EF processed events using the full calorimeter data and using the same anti-$k_{t}$ algorithm as used offline.

The time required for the complete processing of the full ATLAS jet trigger menu per event in the HLT during 2011 had a mean of below 300 ms, well within the required budget for HLT processing.

For the L1 jet trigger, the lowest threshold deployed during 2011 data taking was 10 GeV at the electromagnetic scale. This trigger was fully efficient for offline jets above 45 GeV. The lowest threshold HLT chain which included a L1 jet seed, selected jets reconstructed in the HLT with transverse energy greater than 25 and 30 GeV at L2 and the EF, respectively. These triggers were fully efficient for offline jets above approximately 60 GeV.

For unbiased trigger selection of jets with lower $E_\mathrm{T}$, chains seeded by a random trigger at L1 with a large prescale and passing through L2 – so not requiring a jet seed at either L1 or L2 – were deployed. After accounting for the large prescale, these randomly seeded EF triggers were fully efficient for jets with offline $E_\mathrm{T}$ greater then 25 GeV.

For offline jets with $E_\mathrm{T}>60$ GeV the jets are reconstructed at the EF in the barrel region with a resolution in $E_\mathrm{T}$ with respect to offline jets, of better than 4 % and better than 2.5 % in the endcaps. The performance in terms of offset and resolution of the jet trigger in data is reasonably well modelled by the Monte Carlo detector simulation to better than 1 %, but slightly worse in the crack regions between the barrel and endcap calorimeters. However, the steeply falling jet $p_{\text {T}}$ spectrum means that small differences in the offset between data and the simulation results in differences in the positions of the rising edges of the jet trigger when comparing simulation with data. Physics analyses typically use data only for which the appropriate jet trigger has reached maximal efficiency in order to ameliorate the effect of these differences.

More specialised triggers, intended specifically for searches for signatures of new physics, or for measuring the hadronic decay products of highly boosted massive objects, were operational in 2011 and are seen to perform well. The jet trigger for heavy ion physics was also seen to perform well, benefiting significantly from the full scan approach of the Event Filter to reduce the processing time that would have been required by a purely RoI based approach in such a high occupancy environment.

## Footnotes

1 ATLAS uses a right-handed coordinate system with its origin at the nominal interaction point (IP) in the centre of the detector and the *z*-axis along the beam pipe. The *x*-axis points from the IP to the centre of the LHC ring, and the *y*-axis points upwards. Cylindrical coordinates $(r,\phi )$ are used in the transverse plane, $\phi$ being the azimuthal angle around the *z*-axis. The pseudorapidity is defined in terms of the polar angle $\theta$ as $\eta = -\ln \tan (\theta /2)$.

2 The full calibration was not applied to the trigger jets since it was not available at the time of data taking, but only after the offline reconstruction of the data.
